# Supplementary material for: 3α,7-Dihydroxy-14(13→12)abeo-5β,12α(H),13β(H)-cholan-24-oic Acids Display Neuroprotective Properties in Common Forms of Parkinson’s Disease
Source: Biomolecules. 2022 Dec 30;13(1):76. doi: 10.3390/biom13010076 (PMC9855844; doi:10.3390/biom13010076)

SUPPORTING INFORMATION

3α,7-Dihydroxy-14(13→12)*abeo*-5β,12α(H),13β(H)-cholan-24-oic Acids Display Neuroprotective Properties in Common Forms of Parkinson's Disease

Andreas Luxenburger[1,*], Hannah Clemmens[2], Christopher Hastings[2], Lawrence D. Harris[1], Elizabeth M. Ure[1], Scott A. Cameron[1], Jan Aasly[3,†], Oliver Bandmann[2], Alex Weymouth-Wilson[4], Richard H. Furneaux[1], Heather Mortiboys[2,*]

[1] Ferrier Research Institute, Victoria University of Wellington, 69 Gracefield Rd, Lower Hutt 5040, New Zealand
[2] Sheffield Institute for Translational Neuroscience (SITraN), University of Sheffield, Sheffield, United Kingdom
[3] Department of Neurology, St Olav's Hospital, Trondheim, Norway
[4] ICE Pharma, 68 Weld Street, RD2, Palmerston North 4472, New Zealand
[†] deceased
[*] Andreas.Luxenburger@vuw.ac.nz, h.mortiboys@sheffield.ac.uk

Table of Contents

1. Additional Table S1………..…………………..…………………..…..…...S2
2. X-Ray diffraction data and ORTEP representation of compound **18**….…….S3
3. NMR and HRMS spectra...……………………...………………………..S5
4. HPLC chromatograms……………………...…………...……..……....…...S69

1. Additional Table S1.

Compound	sPD		LRRK2	
	ATP	MMP	ATP	MMP
**3**	170	105	138	136
**4**	120	105	148	118
**7**	130	107	136	116
**24**	140	105	130	105
**25**	97	98	94	98
**29**	95	94	98	96
**UDCA**	146	120	125	125

Table S1. Primary screening data from sPD and LRRK2 mutant patient fibroblasts at one concentration of 100 nM. Data shown are % of DMSO vehicle. Activity shown by compounds **3**, **4**, **7** and **24**. Compounds **25** and **29** were not active.

2. X-Ray Diffraction Data of compound 18

The dataset was collected on an Agilent SuperNova diffractometer fitted with an EOS S2 detector at 120 K and using CuKα radiation (λ = 1.54184 Å). The ORTEP plot is with 30% probability ellipsoids. Minor disordered components and/or solvent molecules have been omitted for clarity.

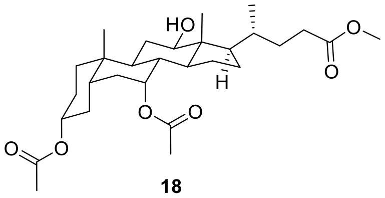

18

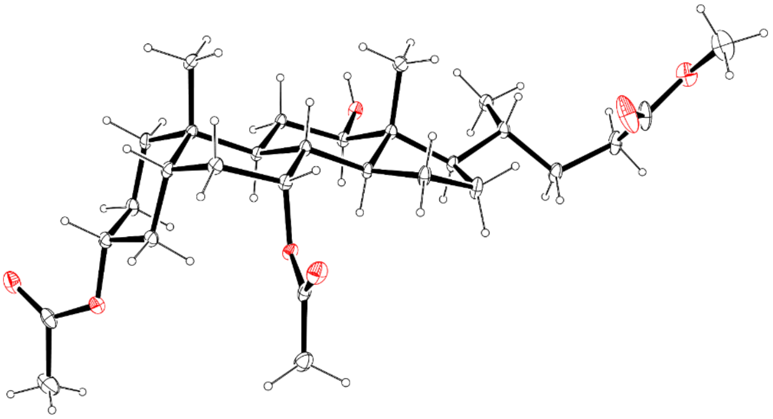

Figure S1. ORTEP diagram of **18** (CCDC 2203402) with 30% probability ellipsoids.

Table S2. Crystal data and structure refinement for compound **18**.

Identification code	**18**
CCDC identifier	2203402
Empirical formula	$C_{29}H_{46}O_7$
Formula weight (g/mol)	506.68
Crystal system	Monoclinic
Space group	$P2_1$
Unit cell dimensions	
a (Å)	6.08670(10)
b (Å)	16.0750(3)
c (Å)	14.0971(2)
α (°)	90
β (°)	99.0140(10)
γ (°)	90
Volume (Å^3)	1362.28(4)
Z	2
Calculated density (Mg/m^3)	1.235
Absorption coefficient (mm^{-1})	0.700
F (000)	552
Crystal size (mm^3)	0.680 × 0.190 × 0.077
Theta range for data collection	4.201 to 71.623°
Index ranges	$-7 \leq h \leq 7$<br>$-17 \leq k \leq 19$<br>$-17 \leq l \leq 17$
Reflections collected	14904
Independent reflections	4853<br>[R(int) = 0.0358]
Completeness to theta	99.9% to 67.684°
Data / restraints / parameters	4853 / 1 / 345
Goodness-of-fit on F^2	1.038
Final R indices [I>2sigma(I)]	R1 = 0.0349<br>wR2 = 0.0905
R indices (all data)	R1 = 0.0358<br>wR2 = 0.0919
Absolute structure parameter	0.06(10)
Largest diff. peak and hole (e.Å^{-3})	0.176 and -0.184

3. NMR Spectra

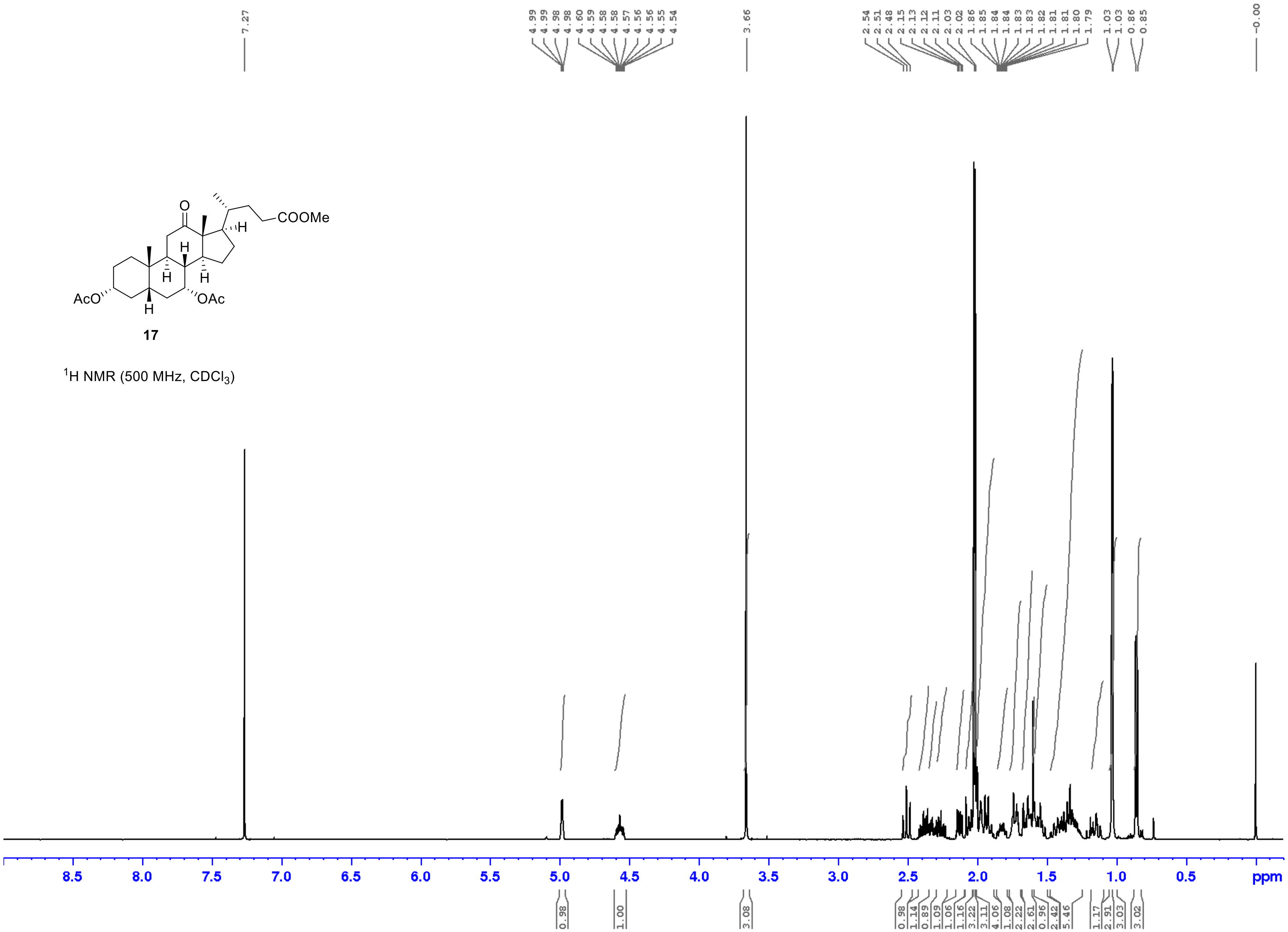

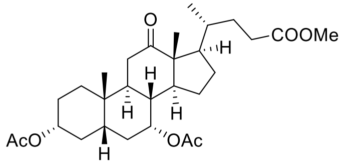

17

^{1}H NMR (500 MHz, $CDCl_3$)

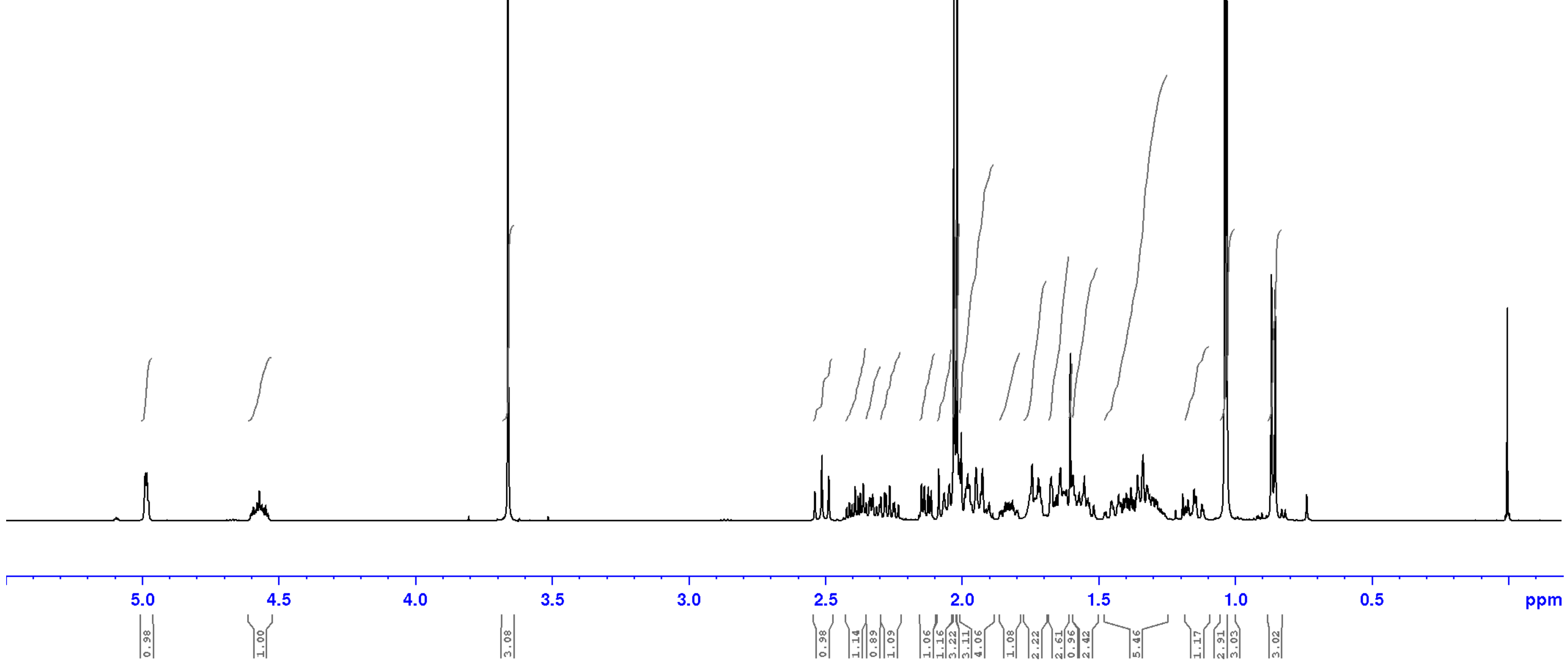

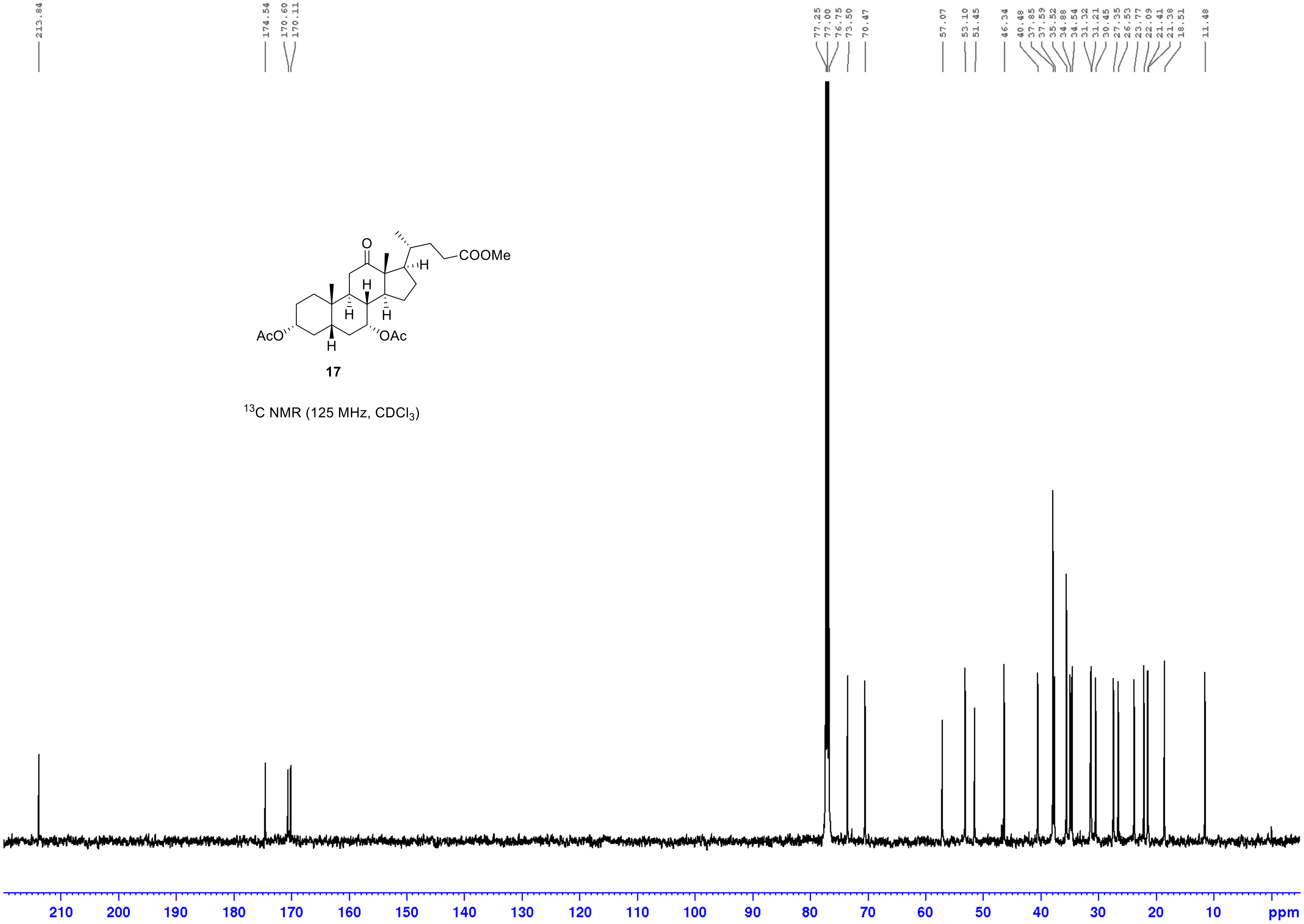
213.84
174.54
170.60
170.11
77.25
77.00
76.75
73.50
70.47
57.07
53.10
51.45
46.34
40.48
37.85
37.59
35.52
34.88
34.54
31.32
31.21
30.45
27.35
26.53
23.77
22.09
21.41
21.38
18.51
11.48
O
COOMe
H
AcO
OAc
17
^{13}C NMR (125 MHz, $CDCl_3$)
210
200
190
180
170
160
150
140
130
120
110
100
90
80
70
60
50
40
30
20
10
ppm

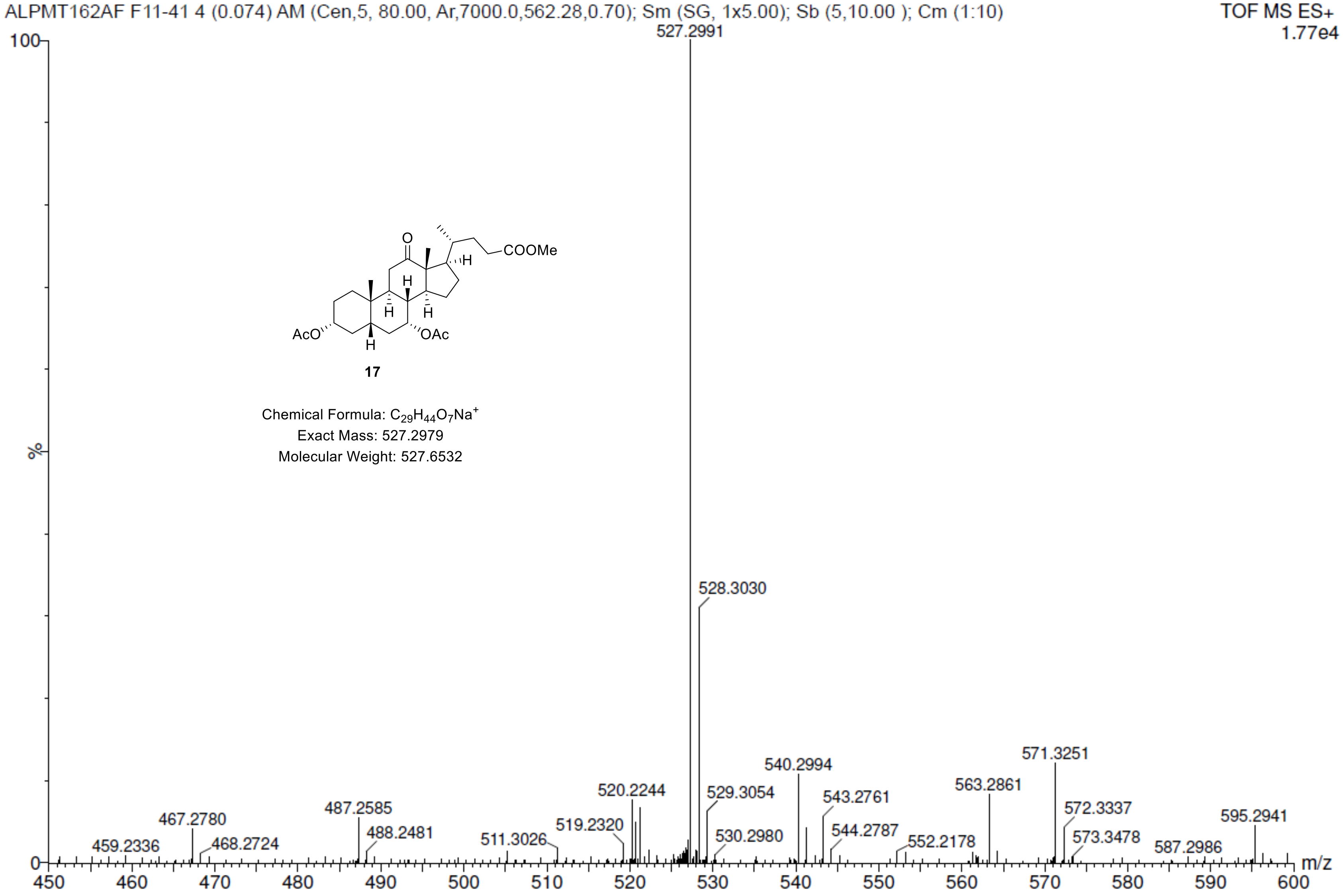
ALPMT162AF F11-41 4 (0.074) AM (Cen,5, 80.00, Ar,7000.0,562.28,0.70); Sm (SG, 1x5.00); Sb (5,10.00); Cm (1:10)
TOF MS ES+
1.77e4
100
%
0
527.2991
528.3030
459.2336
467.2780
468.2724
487.2585
488.2481
511.3026
519.2320
520.2244
529.3054
530.2980
540.2994
543.2761
544.2787
552.2178
563.2861
571.3251
572.3337
573.3478
587.2986
595.2941
450
460
470
480
490
500
510
520
530
540
550
560
570
580
590
600
m/z
O
COOMe
H
AcO
OAc
17
Chemical Formula: $C_{29}H_{44}O_7Na^+$
Exact Mass: 527.2979
Molecular Weight: 527.6532

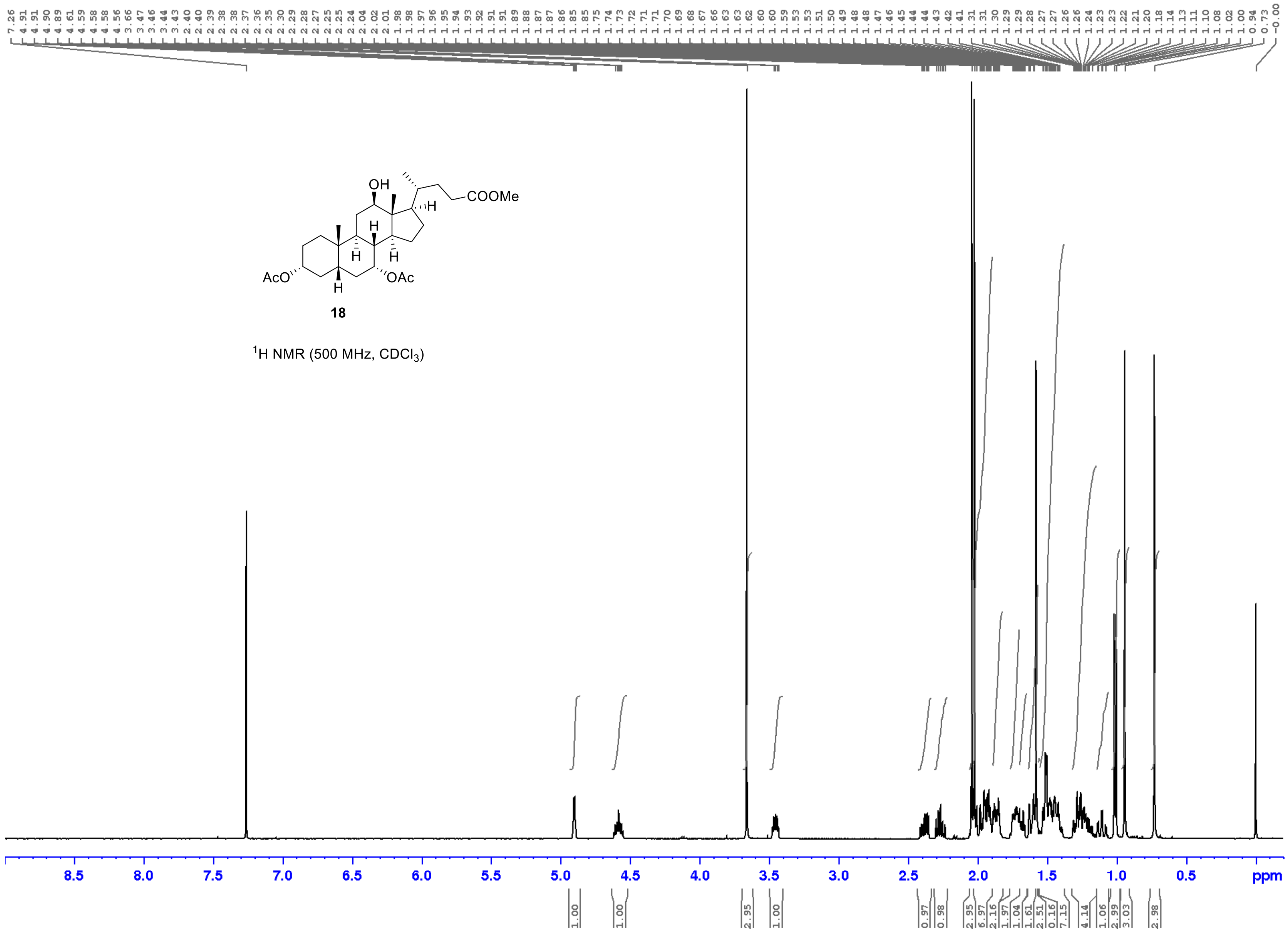

18

^{1}H NMR (500 MHz, $CDCl_3$)

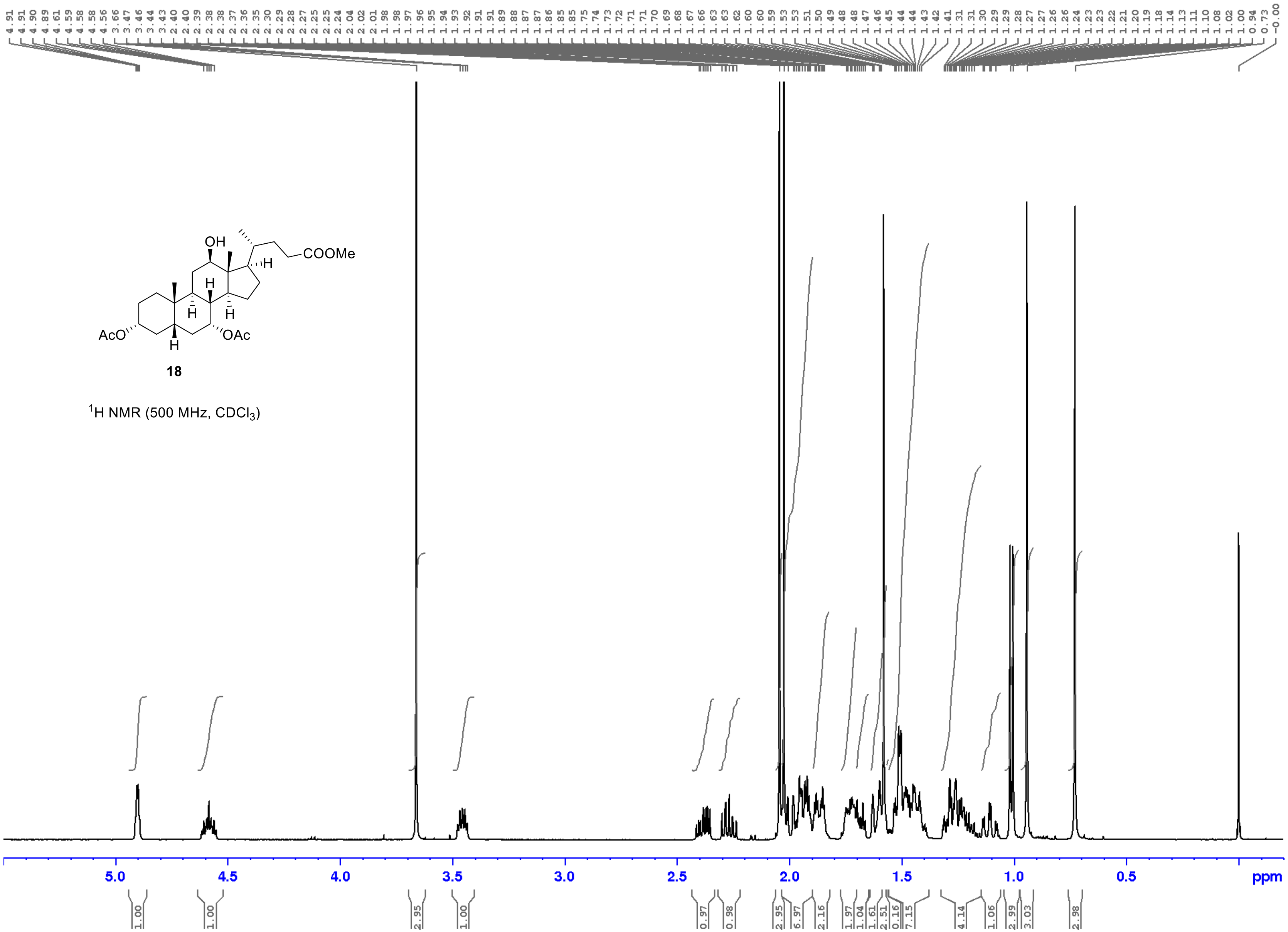
OH
COOMe
H
AcO
OAc
18
¹H NMR (500 MHz, CDCl3)
5.0
4.5
4.0
3.5
3.0
2.5
2.0
1.5
1.0
0.5
ppm

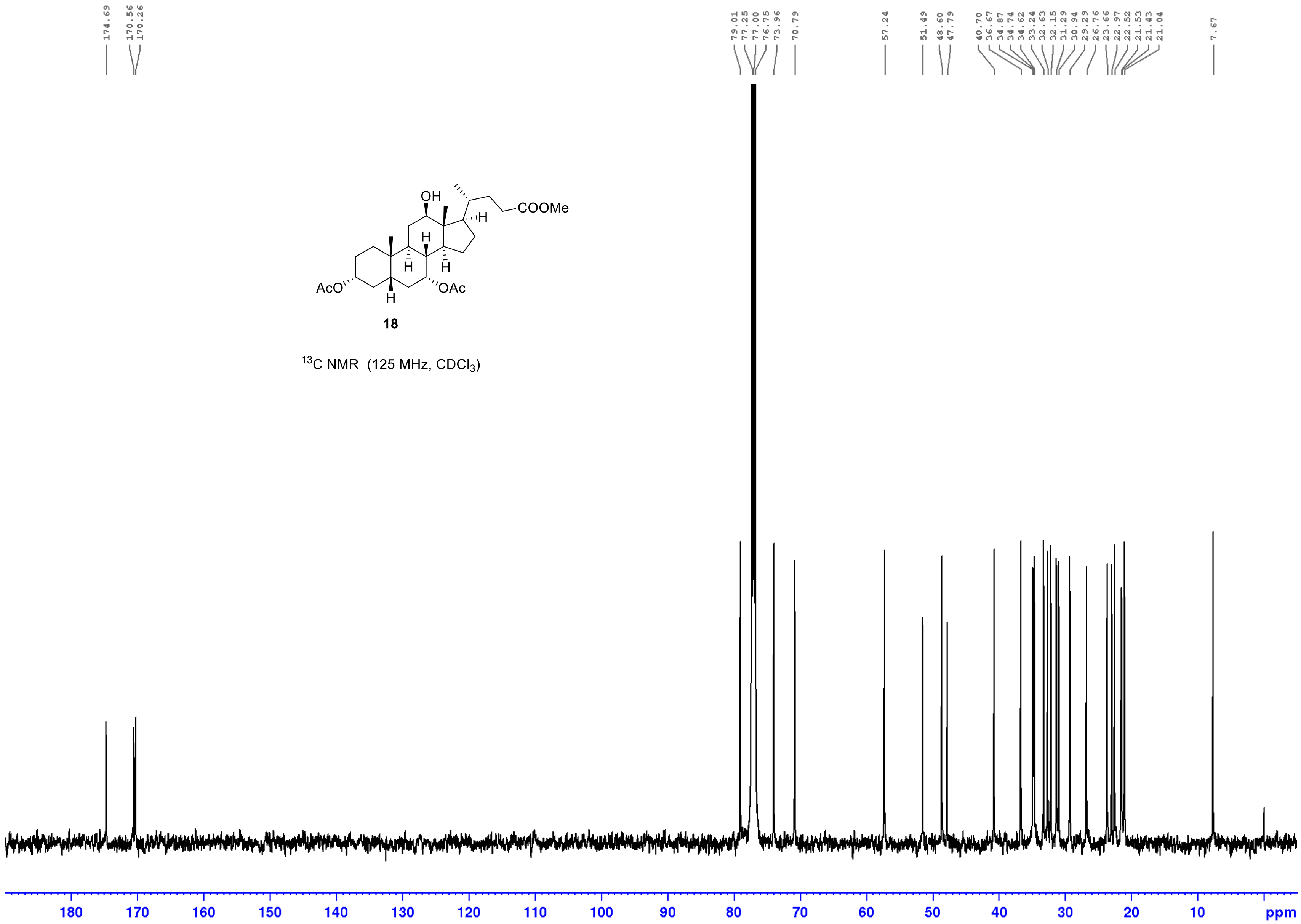

174.69
170.56
170.26
79.01
77.25
77.00
76.75
73.96
70.79
57.24
51.49
48.60
47.79
40.70
36.67
34.87
34.74
34.62
33.24
32.63
32.15
31.29
30.94
29.29
26.76
23.66
22.97
22.52
21.53
21.43
21.04
7.67
OH
COOMe
H
AcO
OAc
18
^{13}C NMR (125 MHz, $CDCl_3$)
180
170
160
150
140
130
120
110
100
90
80
70
60
50
40
30
20
10
ppm

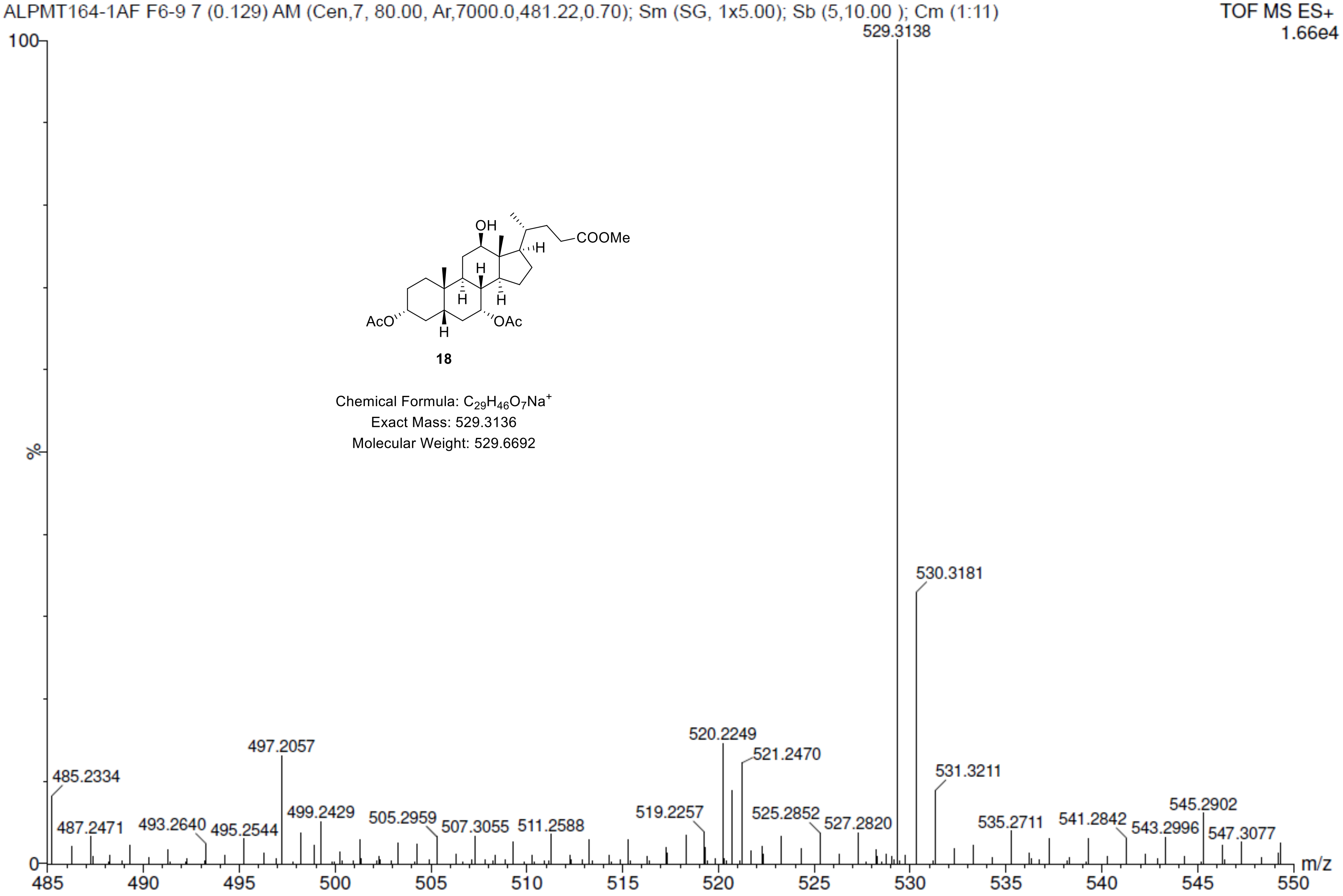
ALPMT164-1AF F6-9 7 (0.129) AM (Cen,7, 80.00, Ar,7000.0,481.22,0.70); Sm (SG, 1x5.00); Sb (5,10.00); Cm (1:11)
TOF MS ES+
1.66e4
100
%
0
529.3138
530.3181
531.3211
520.2249
521.2470
497.2057
485.2334
487.2471
493.2640
495.2544
499.2429
505.2959
507.3055
511.2588
519.2257
525.2852
527.2820
535.2711
541.2842
543.2996
545.2902
547.3077
OH
COOMe
H
AcO
OAc
18
Chemical Formula: $C_{29}H_{46}O_7Na^+$
Exact Mass: 529.3136
Molecular Weight: 529.6692
485
490
495
500
505
510
515
520
525
530
535
540
545
550
m/z

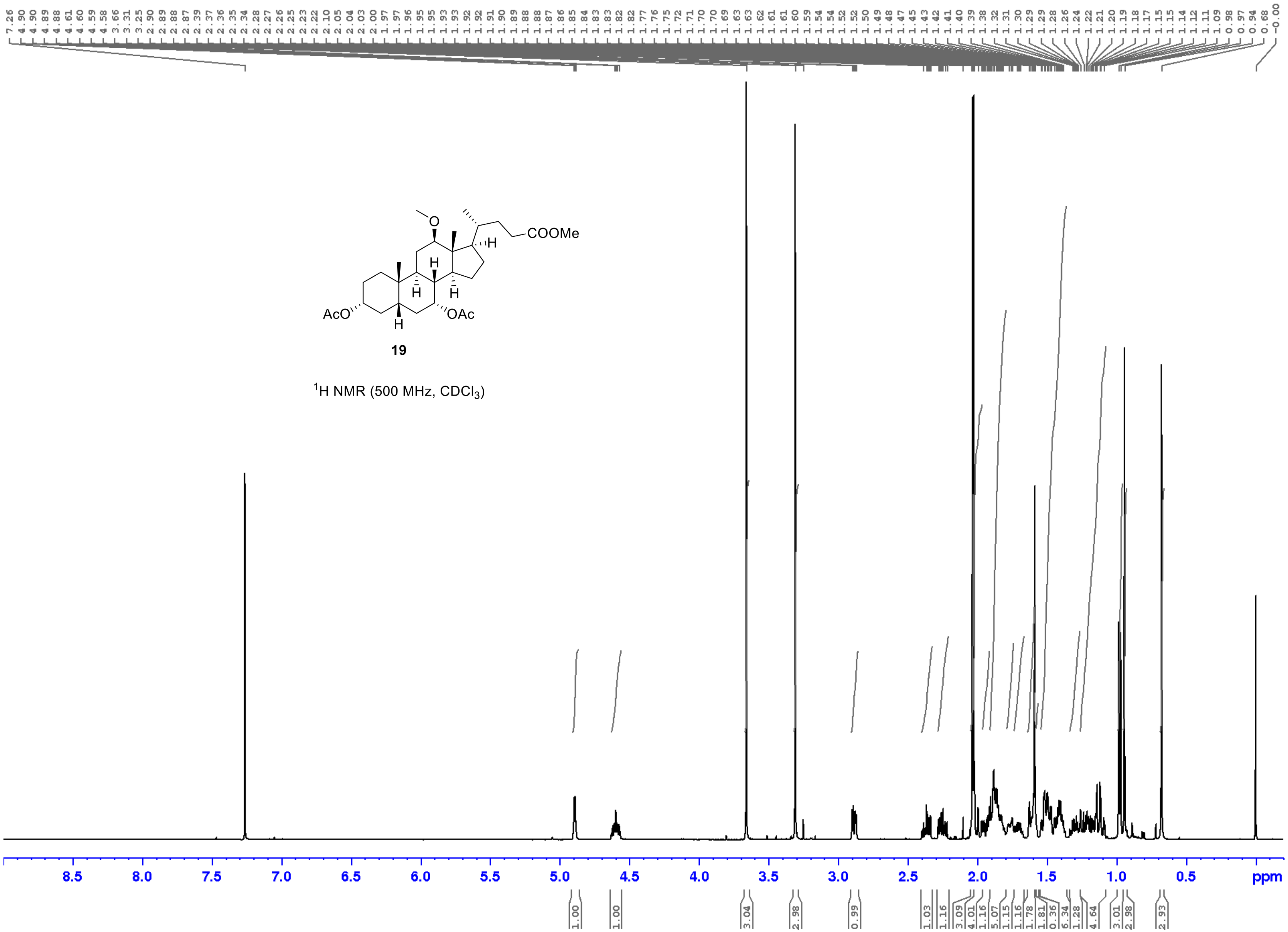

19

^{1}H NMR (500 MHz, $CDCl_3$)

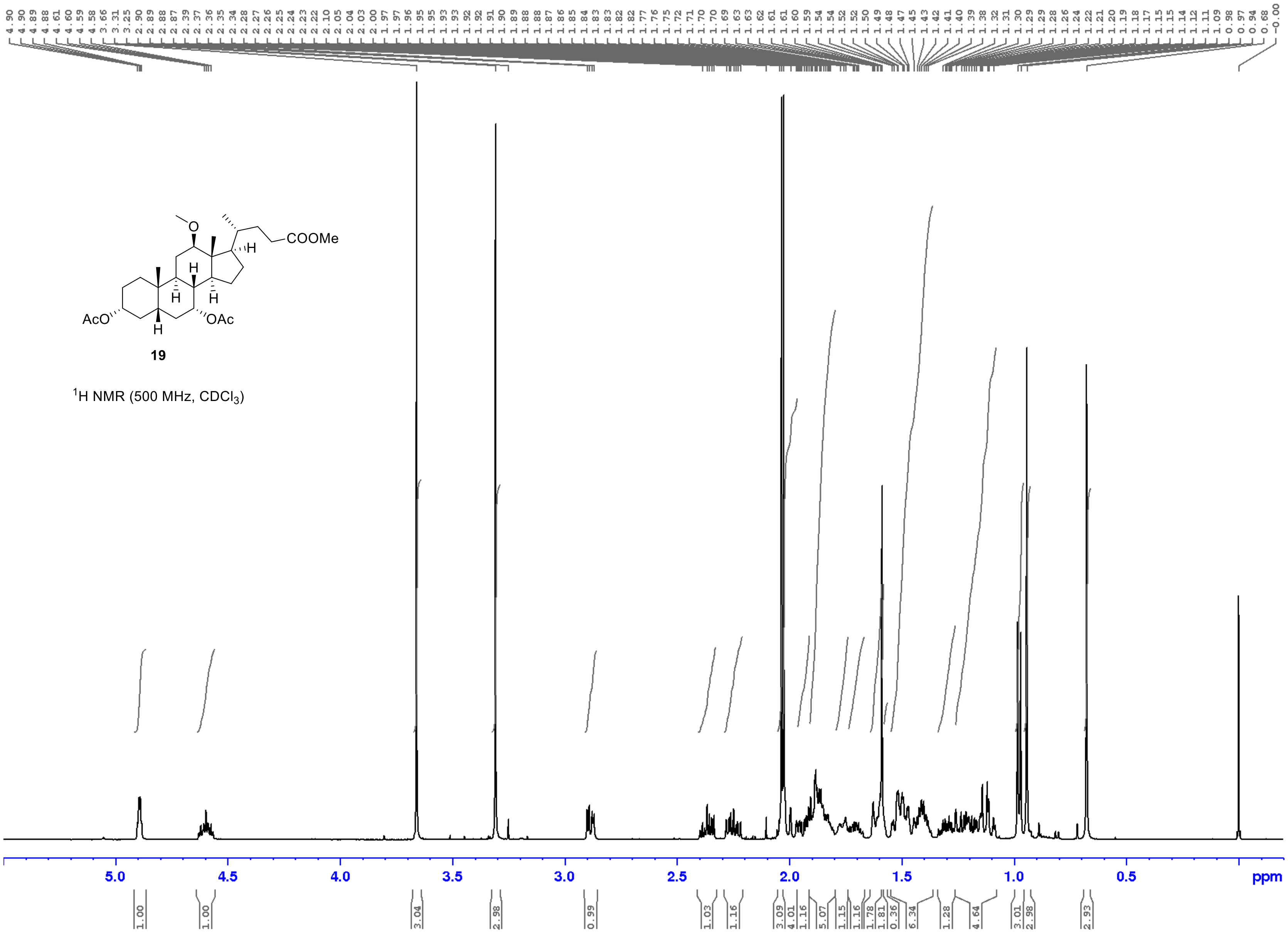
COOMe
AcO
OAc
19
¹H NMR (500 MHz, CDCl₃)
ppm

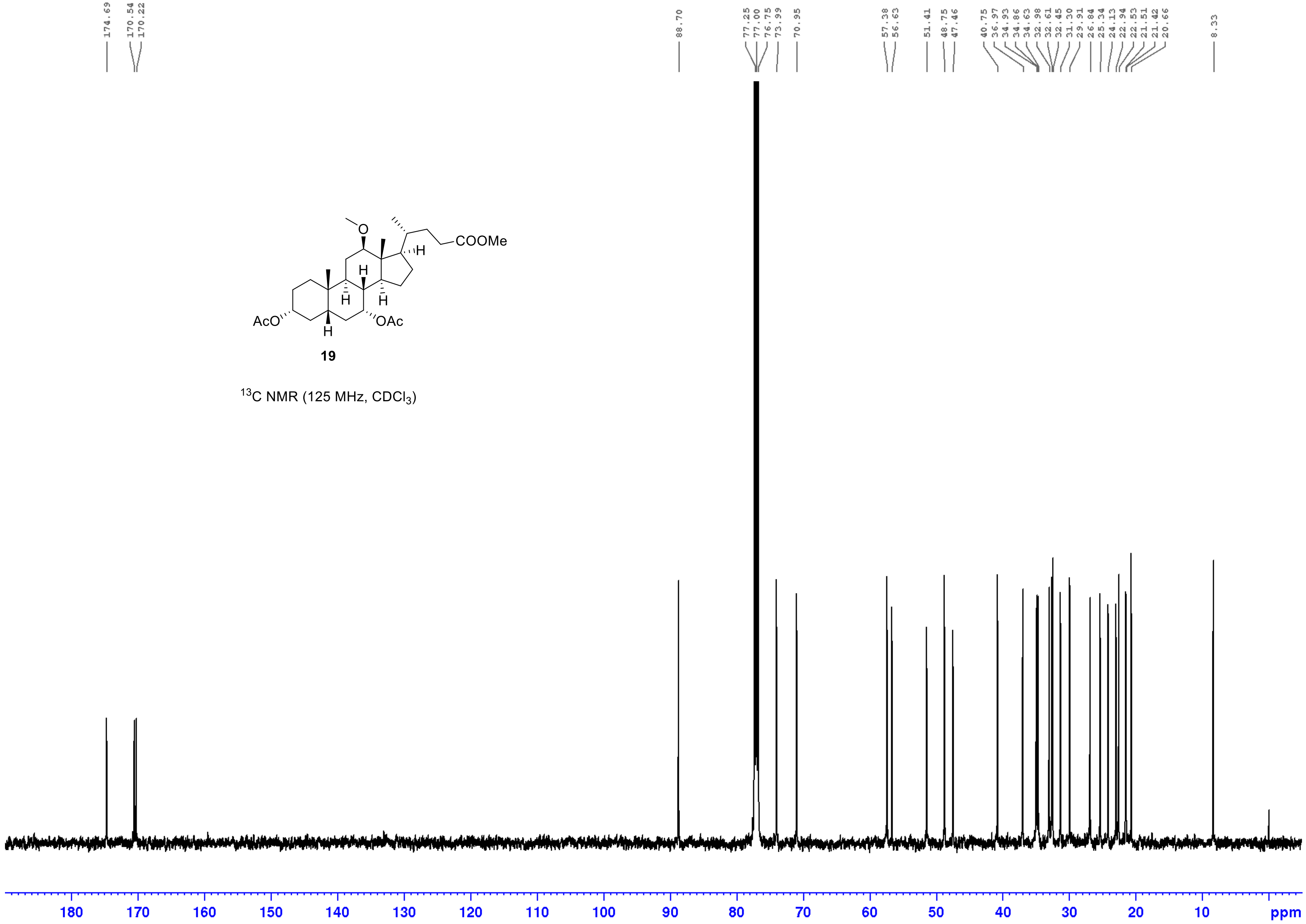

19

^{13}C NMR (125 MHz, $CDCl_3$)

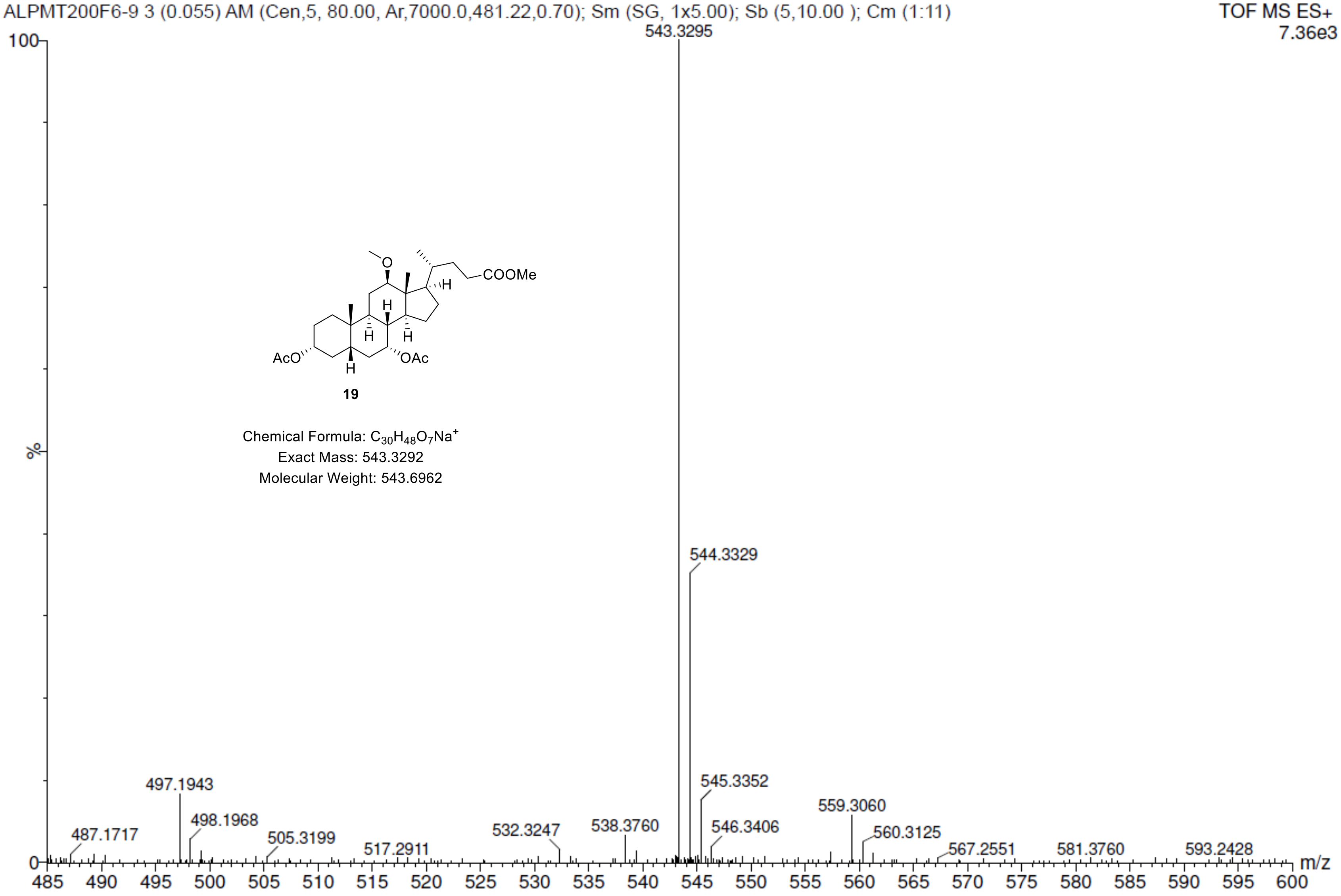
ALPMT200F6-9 3 (0.055) AM (Cen,5, 80.00, Ar,7000.0,481.22,0.70); Sm (SG, 1x5.00); Sb (5,10.00); Cm (1:11)
TOF MS ES+
7.36e3
COOMe
AcO
OAc
19
Chemical Formula: $C_{30}H_{48}O_7Na^+$
Exact Mass: 543.3292
Molecular Weight: 543.6962
543.3295
544.3329
545.3352
546.3406
497.1943
498.1968
487.1717
505.3199
517.2911
532.3247
538.3760
559.3060
560.3125
567.2551
581.3760
593.2428
%
m/z

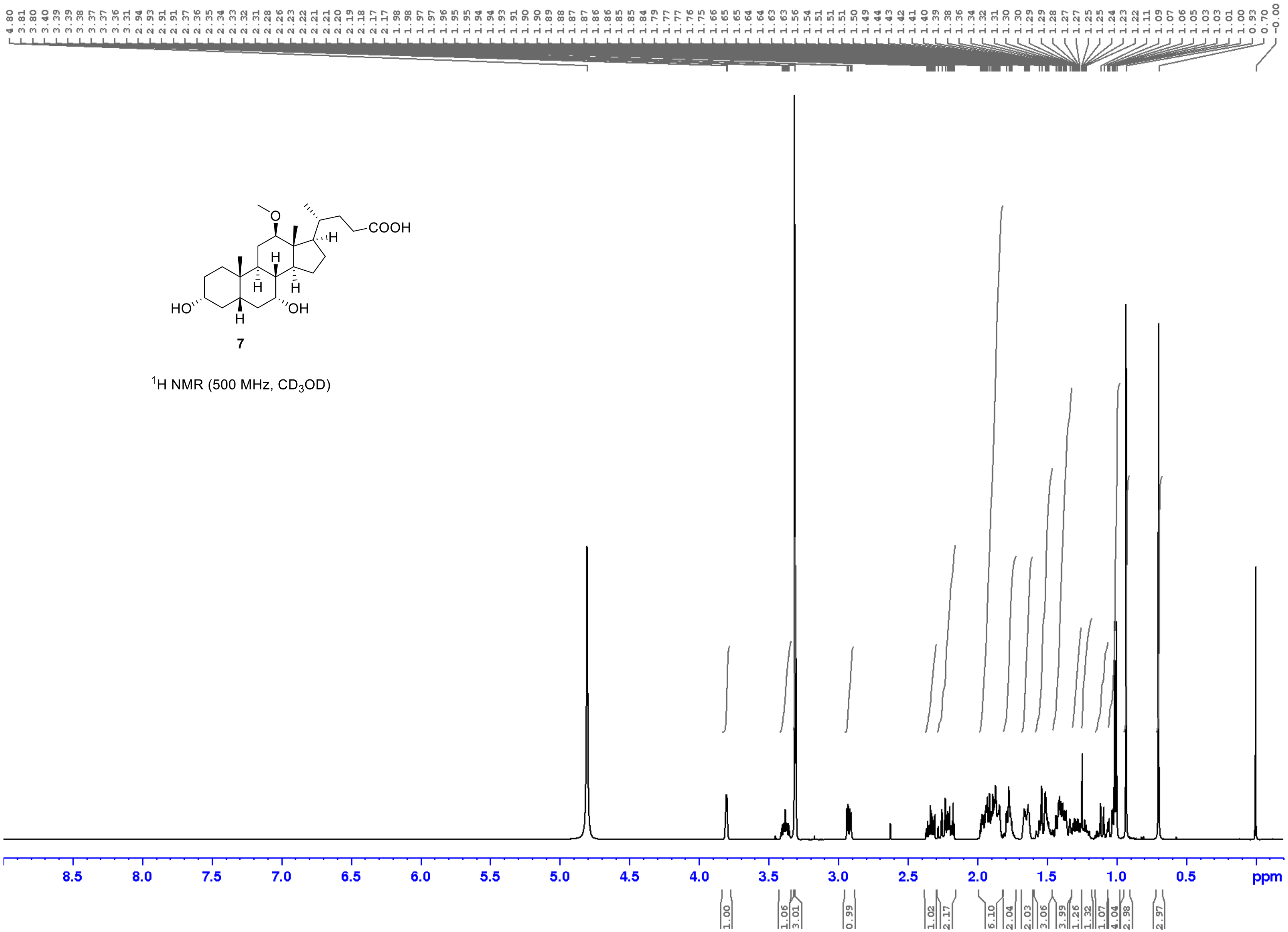
4.80 3.81 3.80 3.40 3.39 3.39 3.38 3.37 3.37 3.36 3.31 2.94 2.93 2.91 2.91 2.37 2.36 2.35 2.34 2.33 2.32 2.31 2.28 2.26 2.23 2.22 2.21 2.21 2.20 2.19 2.18 2.17 2.17 1.98 1.98 1.97 1.97 1.96 1.95 1.94 1.94 1.93 1.91 1.90 1.90 1.89 1.88 1.87 1.87 1.86 1.86 1.85 1.85 1.84 1.79 1.77 1.77 1.76 1.75 1.66 1.65 1.65 1.64 1.63 1.63 1.56 1.54 1.51 1.51 1.51 1.50 1.49 1.44 1.43 1.42 1.41 1.40 1.39 1.38 1.36 1.34 1.32 1.31 1.30 1.30 1.29 1.29 1.28 1.27 1.27 1.25 1.25 1.24 1.23 1.22 1.11 1.09 1.07 1.06 1.05 1.03 1.03 1.01 1.00 0.93 0.70 -0.00
COOH
HO
OH
O
H
7
1H NMR (500 MHz, CD3OD)
8.5 8.0 7.5 7.0 6.5 6.0 5.5 5.0 4.5 4.0 3.5 3.0 2.5 2.0 1.5 1.0 0.5 ppm
1.00 1.06 3.01 0.99 1.02 2.17 6.10 2.04 2.03 3.06 3.99 1.26 1.32 1.07 4.04 2.98 2.97

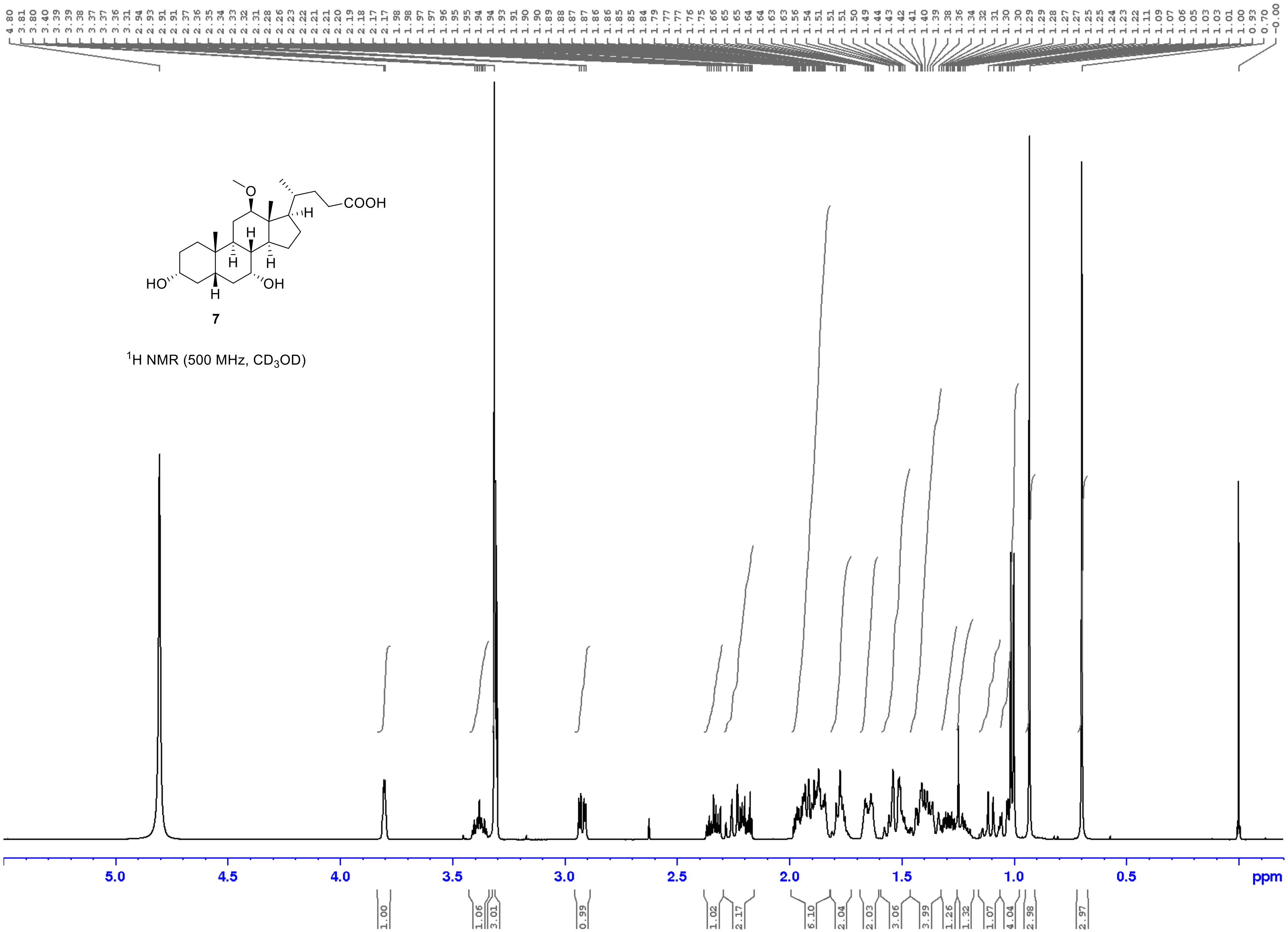
COOH
HO
OH
7
1H NMR (500 MHz, CD3OD)
5.0
4.5
4.0
3.5
3.0
2.5
2.0
1.5
1.0
0.5
ppm

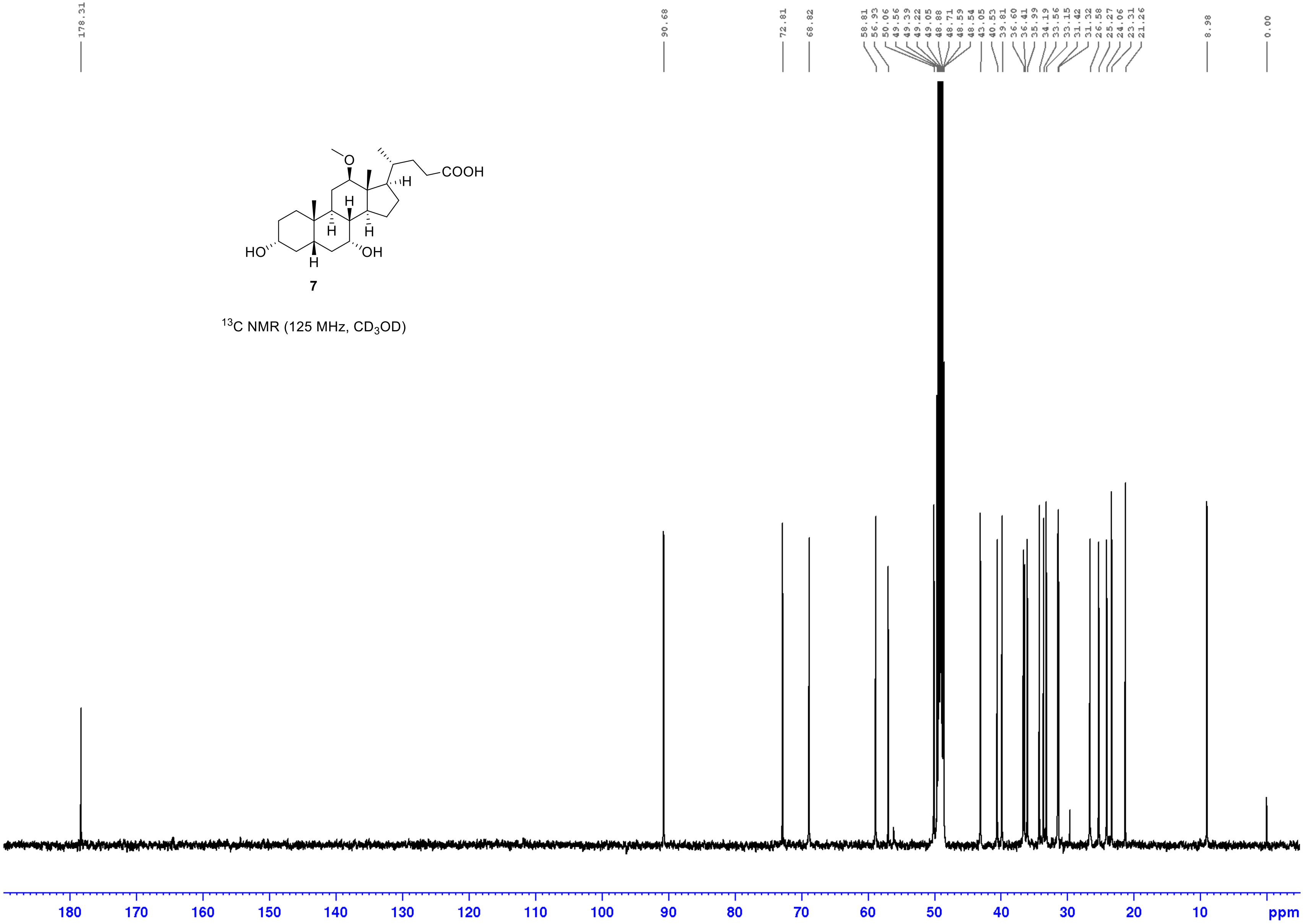

^{13}C NMR (125 MHz, CD_3OD)

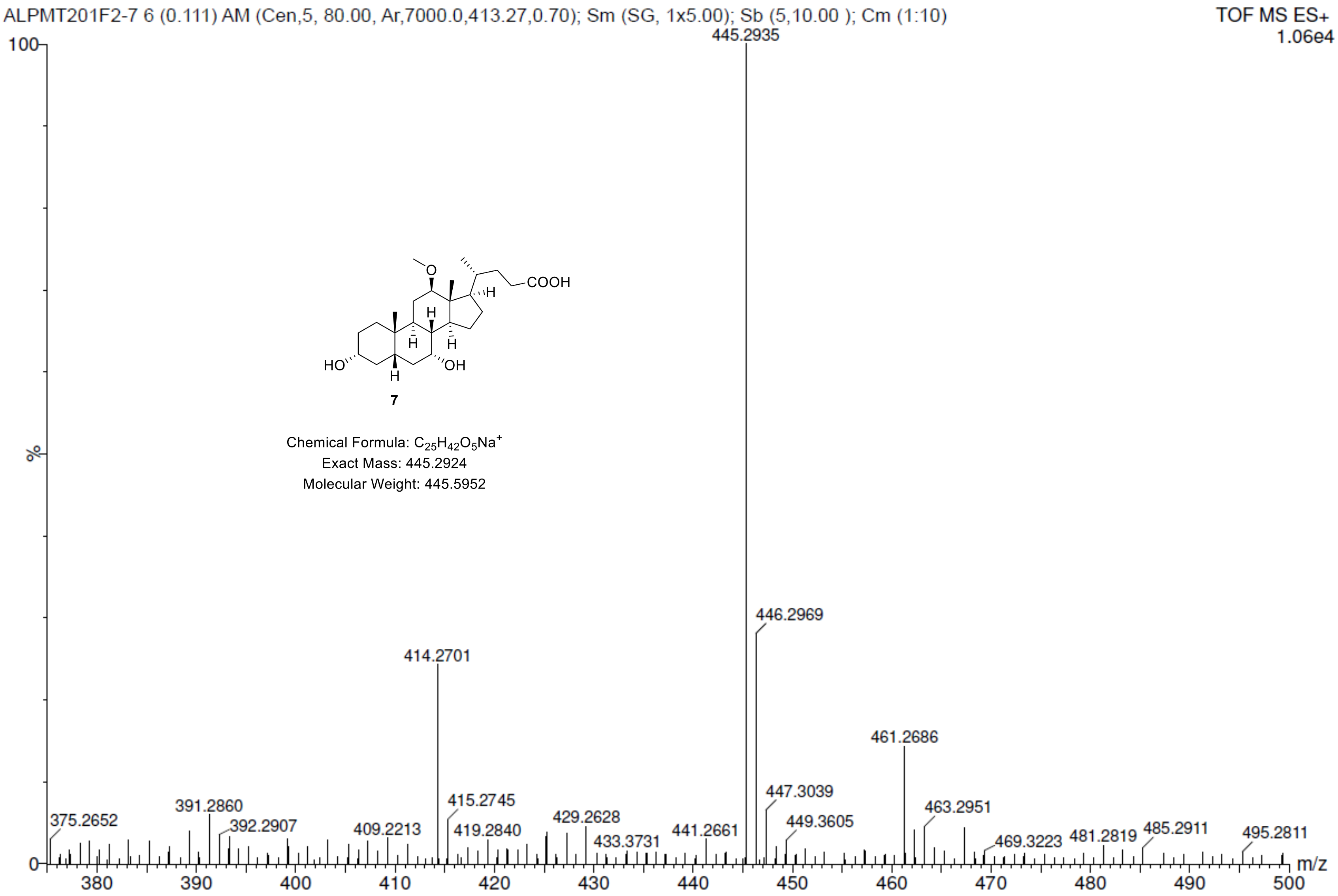

ALPMT201F2-7 6 (0.111) AM (Cen,5, 80.00, Ar,7000.0,413.27,0.70); Sm (SG, 1x5.00); Sb (5,10.00); Cm (1:10)
TOF MS ES+
1.06e4
445.2935
446.2969
414.2701
461.2686
447.3039
463.2951
415.2745
375.2652
391.2860
392.2907
409.2213
419.2840
429.2628
433.3731
441.2661
449.3605
469.3223
481.2819
485.2911
495.2811
%
m/z
COOH
HO
OH
7
Chemical Formula: $C_{25}H_{42}O_5Na^+$
Exact Mass: 445.2924
Molecular Weight: 445.5952

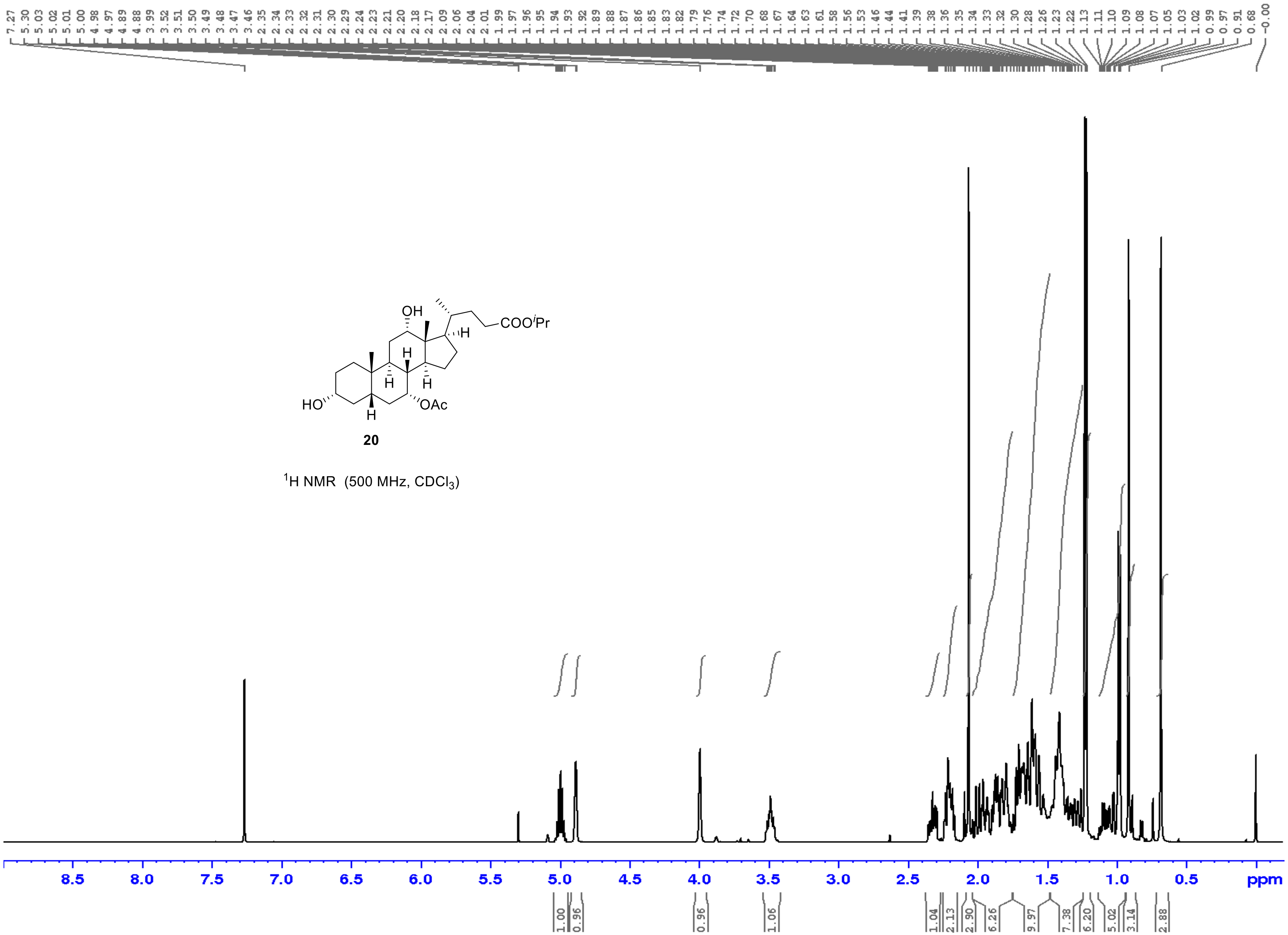
7.27 5.30 5.03 5.02 5.01 5.00 4.98 4.97 4.89 4.88 3.99 3.52 3.51 3.50 3.49 3.48 3.47 3.46 2.35 2.34 2.33 2.32 2.31 2.30 2.29 2.24 2.23 2.21 2.20 2.18 2.17 2.09 2.06 2.04 2.01 1.99 1.97 1.96 1.95 1.94 1.93 1.92 1.89 1.88 1.87 1.86 1.85 1.83 1.82 1.79 1.76 1.74 1.72 1.70 1.68 1.67 1.64 1.63 1.61 1.58 1.56 1.53 1.46 1.44 1.41 1.39 1.38 1.36 1.35 1.34 1.33 1.32 1.30 1.28 1.26 1.23 1.22 1.13 1.11 1.10 1.09 1.08 1.07 1.05 1.03 1.02 0.99 0.97 0.91 0.68 -0.00
OH
COO^{i}Pr
H
HO
OAc
20
^{1}H NMR (500 MHz, $CDCl_3$)
8.5 8.0 7.5 7.0 6.5 6.0 5.5 5.0 4.5 4.0 3.5 3.0 2.5 2.0 1.5 1.0 0.5 ppm
1.00 0.96 0.96 1.06 1.04 2.13 2.90 6.26 9.97 7.38 6.20 5.02 3.14 2.88

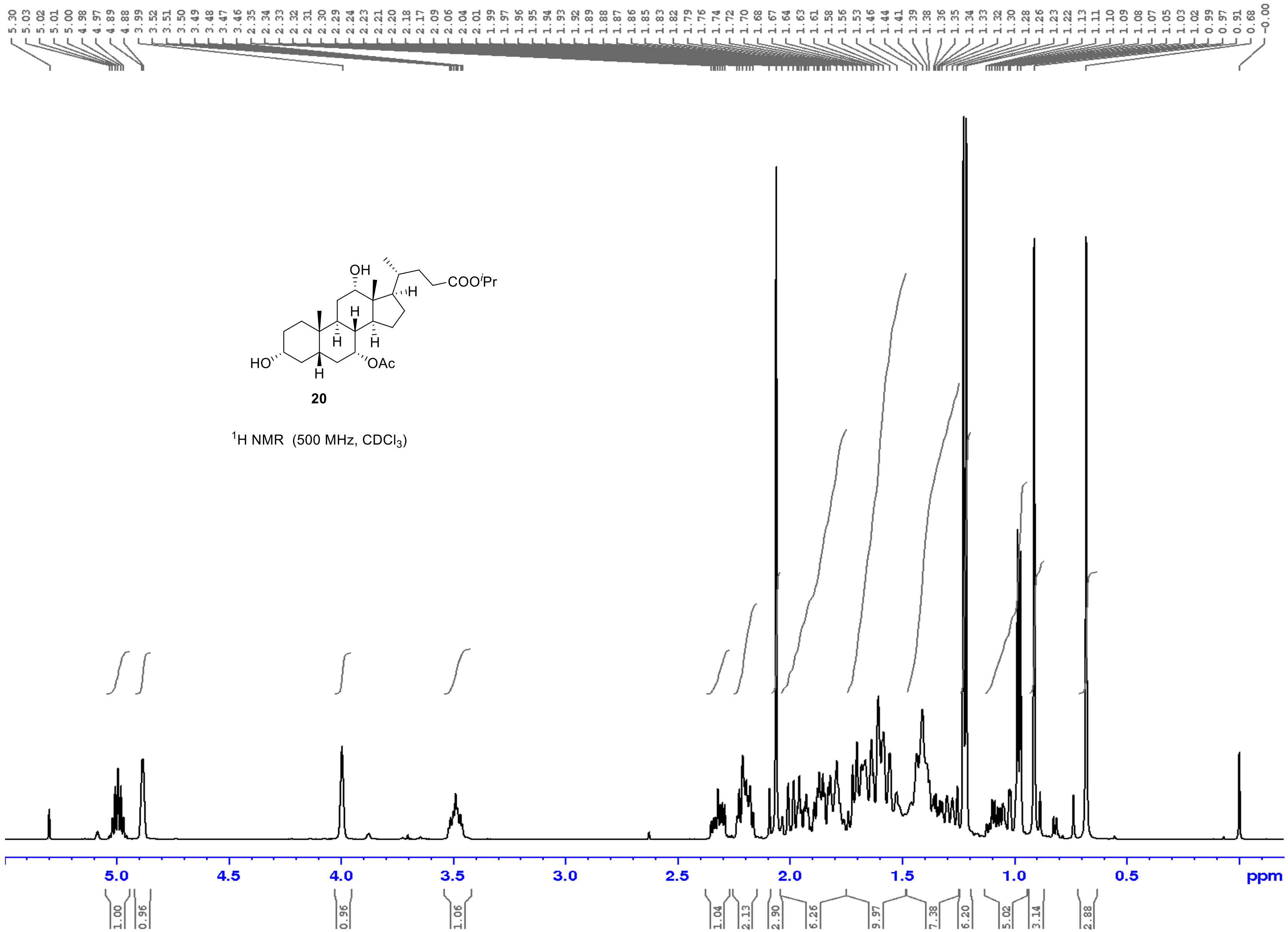
20
^1H NMR (500 MHz, $CDCl_3$)

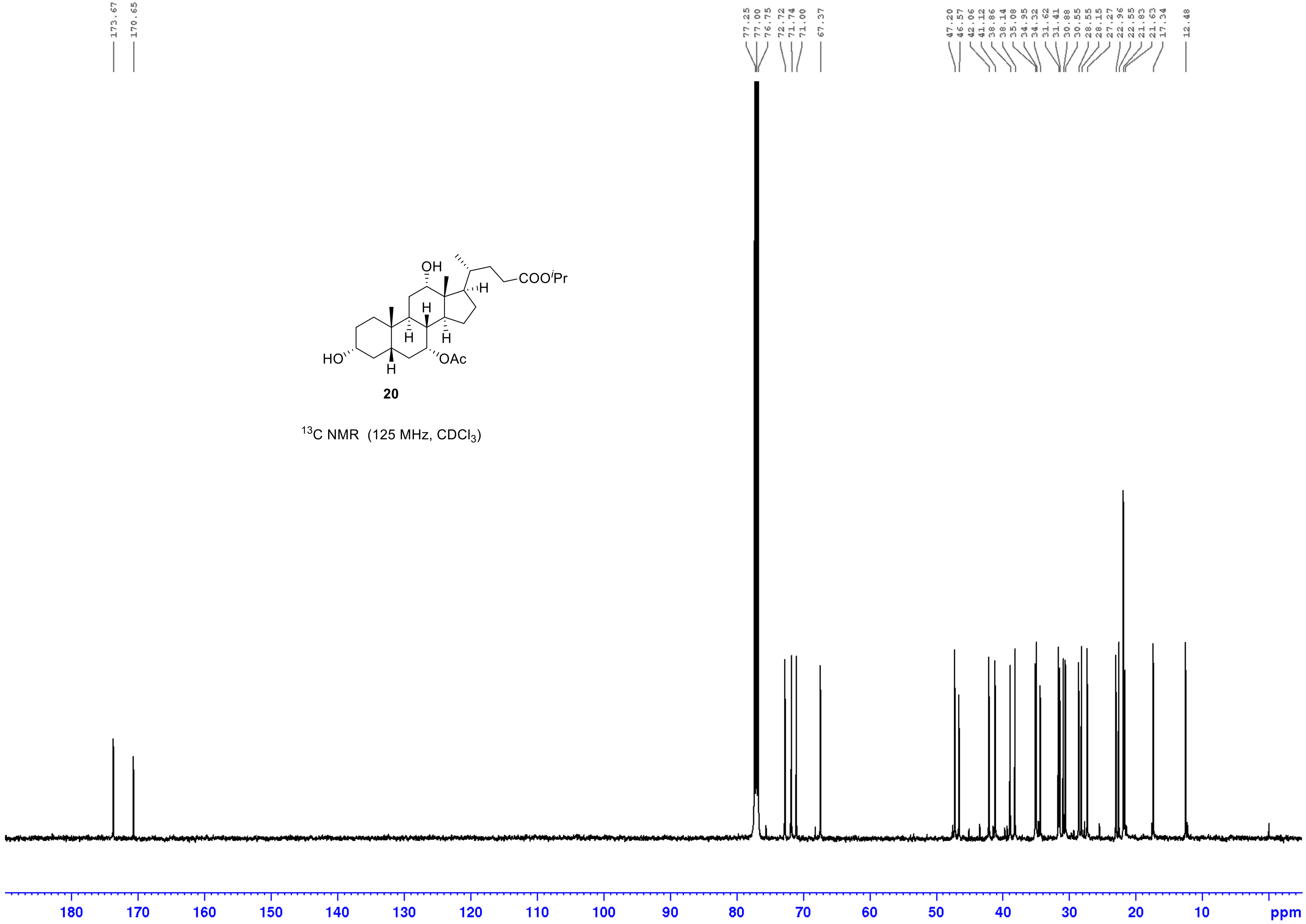

OH
COO^{i}Pr
H
HO
OAc
20
^{13}C NMR (125 MHz, $CDCl_3$)
173.67
170.65
77.25
77.00
76.75
72.72
71.74
71.00
67.37
47.20
46.57
42.06
41.12
38.86
38.14
35.08
34.95
34.32
31.62
31.41
30.88
30.55
28.55
28.15
27.27
22.96
22.55
21.83
21.63
17.34
12.48
180
170
160
150
140
130
120
110
100
90
80
70
60
50
40
30
20
10
ppm

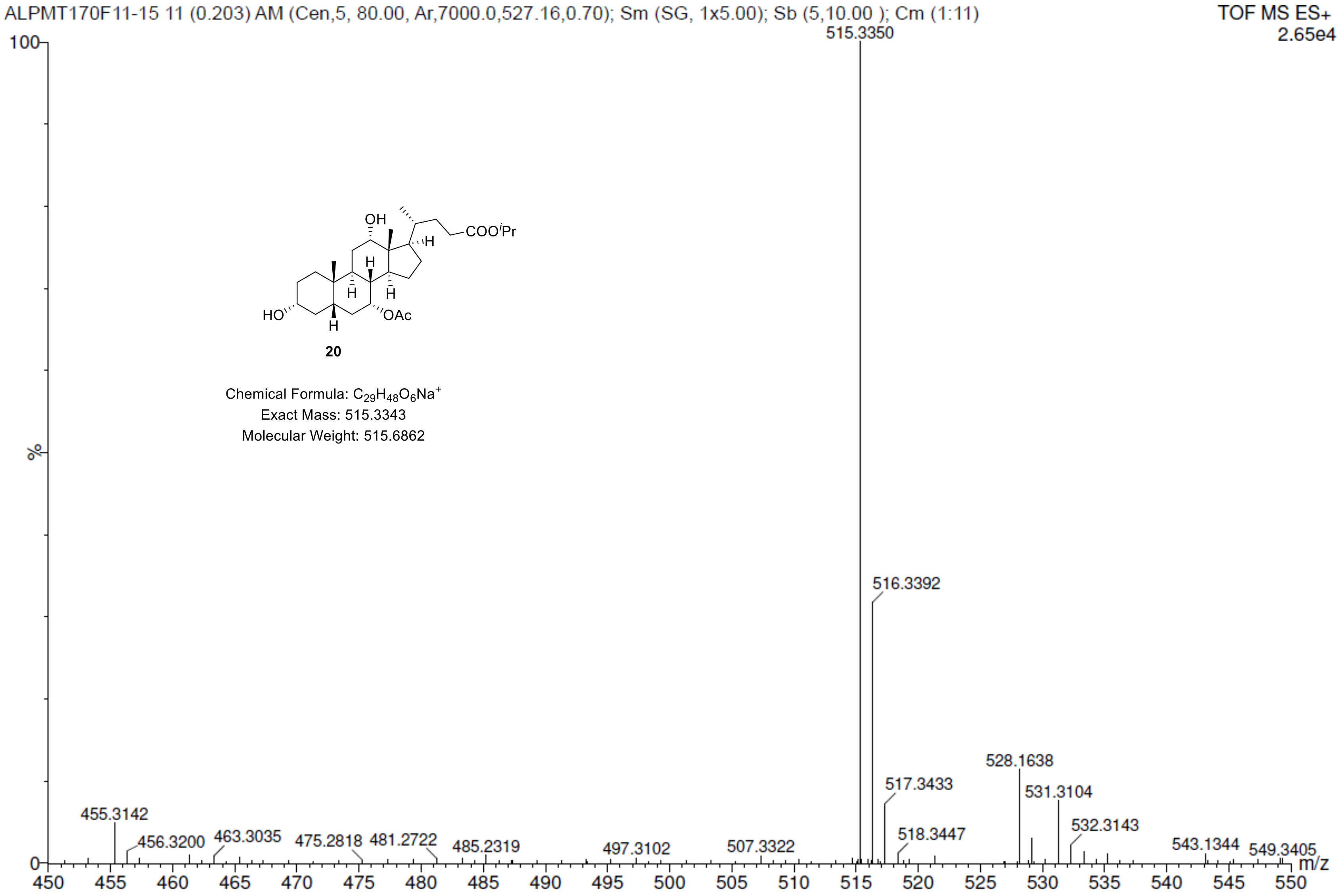
ALPMT170F11-15 11 (0.203) AM (Cen,5, 80.00, Ar,7000.0,527.16,0.70); Sm (SG, 1x5.00); Sb (5,10.00); Cm (1:11)
TOF MS ES+
2.65e4
515.3350
516.3392
517.3433
518.3447
528.1638
531.3104
532.3143
543.1344
549.3405
455.3142
456.3200
463.3035
475.2818
481.2722
485.2319
497.3102
507.3322
%
m/z
OH
COO^{i}Pr
HO
OAc
20
Chemical Formula: $C_{29}H_{48}O_6Na^+$
Exact Mass: 515.3343
Molecular Weight: 515.6862

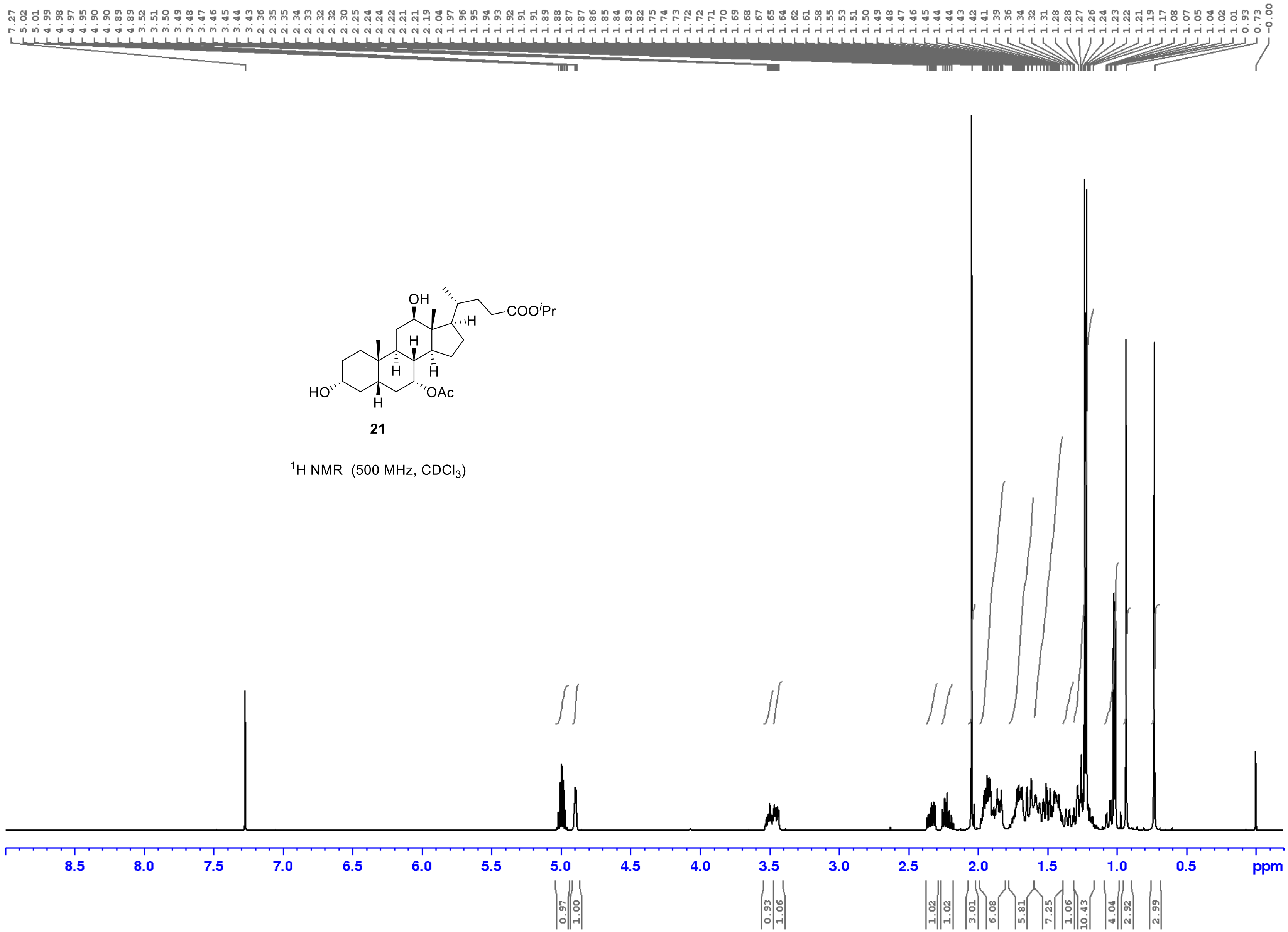

21

^{1}H NMR (500 MHz, $CDCl_3$)

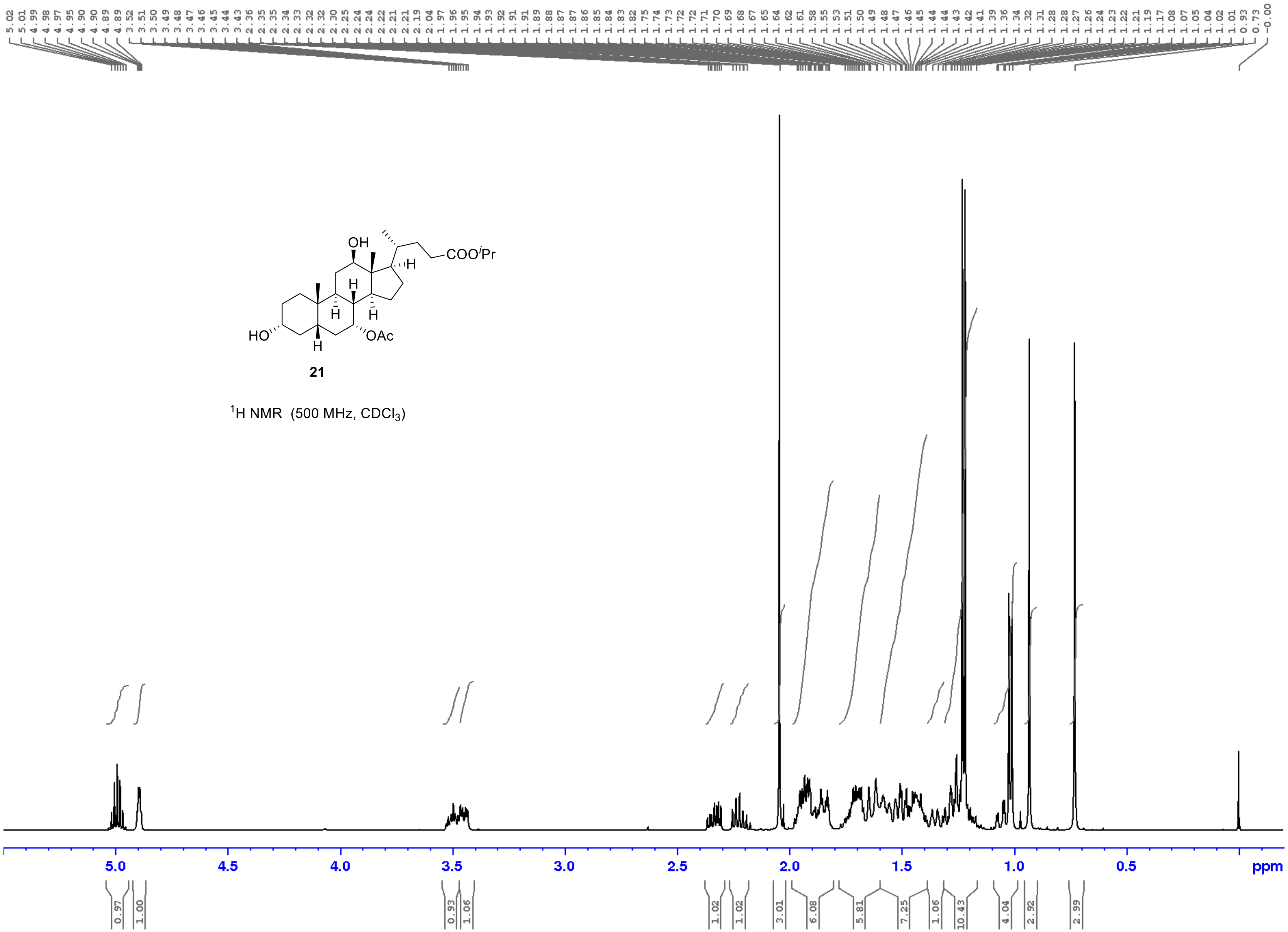
21
^{1}H NMR (500 MHz, CDCl$_3$)
COO^{i}Pr
OH
HO
OAc
H
5.0
4.5
4.0
3.5
3.0
2.5
2.0
1.5
1.0
0.5
ppm
0.97
1.00
0.93
1.06
1.02
1.02
3.01
6.08
5.81
7.25
1.06
10.43
4.04
2.92
2.99

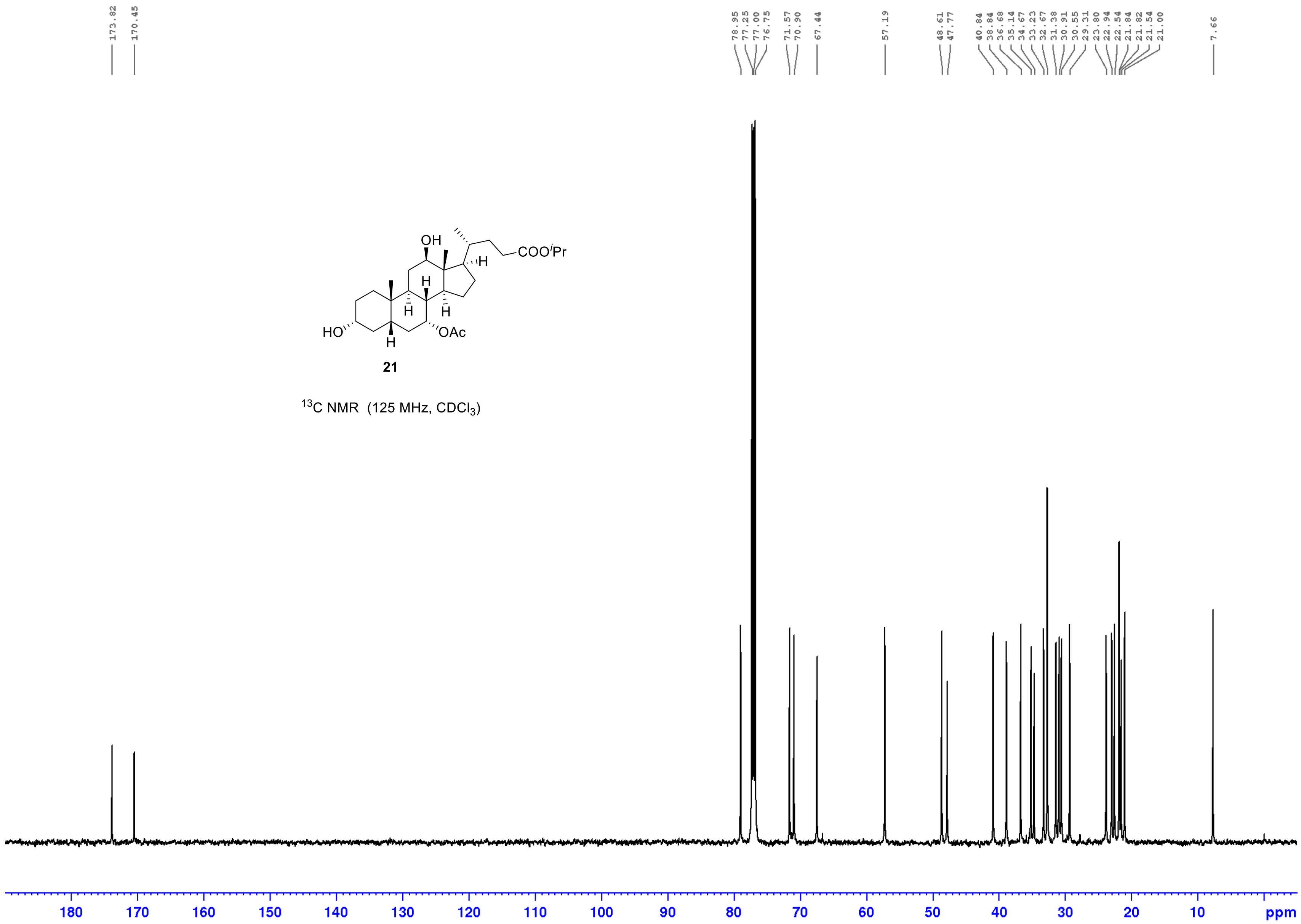
173.82
170.45
78.95
77.25
77.00
76.75
71.57
70.90
67.44
57.19
48.61
47.77
40.84
38.84
36.68
35.14
34.67
33.23
32.67
31.38
30.91
30.55
29.31
23.80
22.94
22.54
21.84
21.82
21.54
21.00
7.66
OH
COO^{i}Pr
H
HO
OAc
21
^{13}C NMR (125 MHz, $CDCl_3$)
180
170
160
150
140
130
120
110
100
90
80
70
60
50
40
30
20
10
ppm

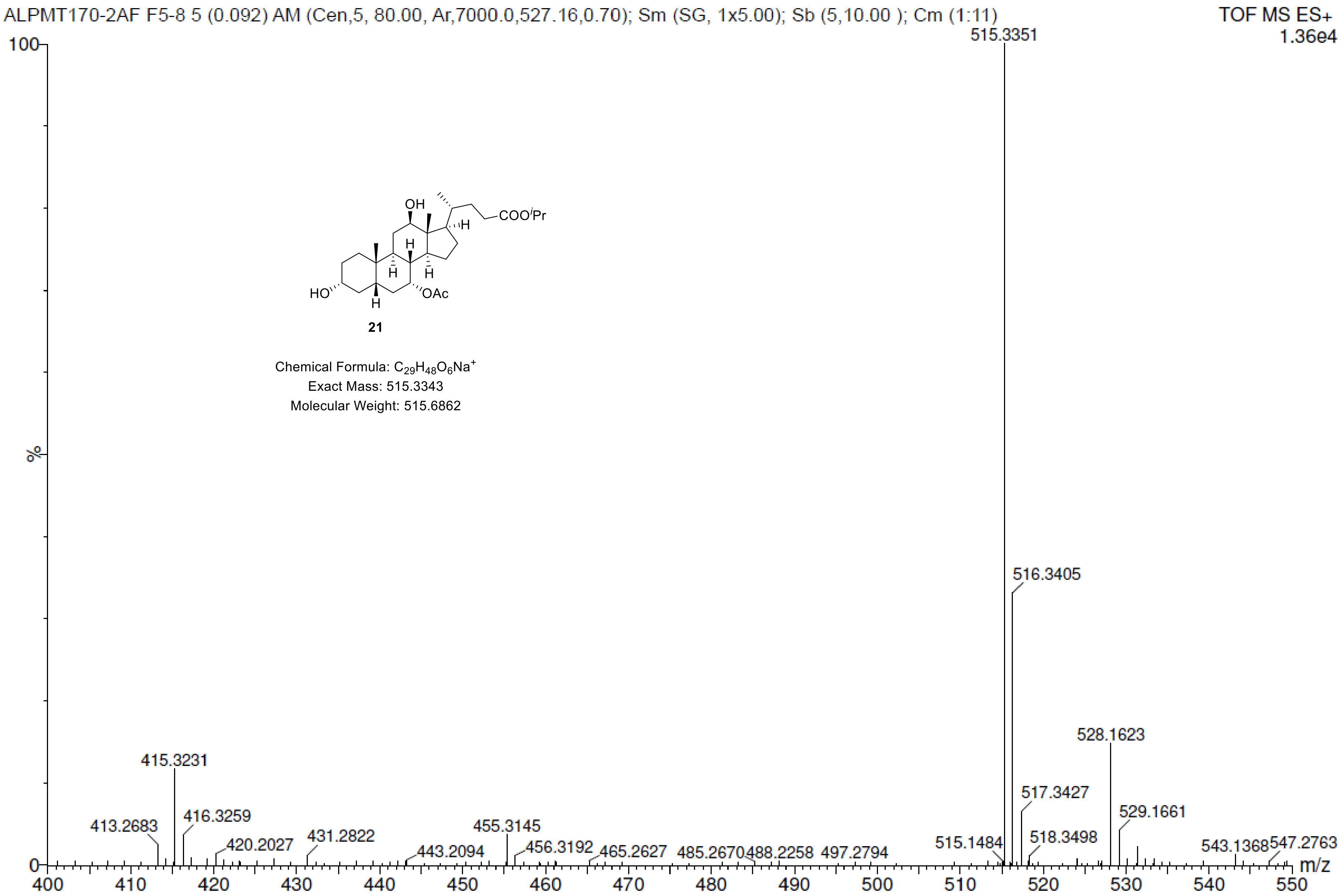
ALPMT170-2AF F5-8 5 (0.092) AM (Cen,5, 80.00, Ar,7000.0,527.16,0.70); Sm (SG, 1x5.00); Sb (5,10.00); Cm (1:11)
TOF MS ES+
1.36e4
100
%
0
OH
COO^{i}Pr
H
HO
OAc
21
Chemical Formula: $C_{29}H_{48}O_6Na^+$
Exact Mass: 515.3343
Molecular Weight: 515.6862
515.3351
516.3405
528.1623
517.3427
529.1661
518.3498
515.1484
543.1368
547.2763
415.3231
413.2683
416.3259
420.2027
431.2822
455.3145
443.2094
456.3192
465.2627
485.2670
488.2258
497.2794
400
410
420
430
440
450
460
470
480
490
500
510
520
530
540
550
m/z

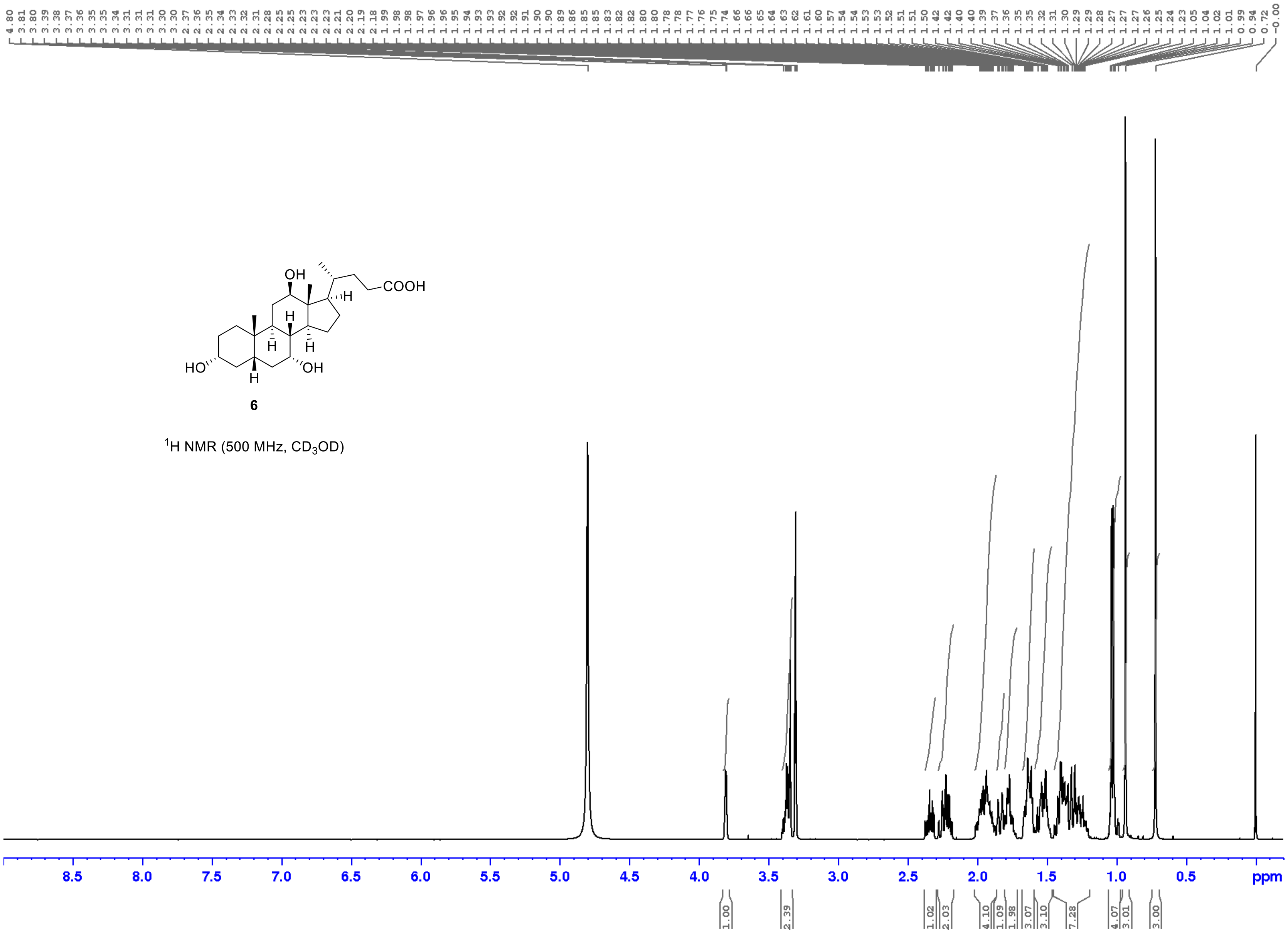

6

^{1}H NMR (500 MHz, CD_3OD)

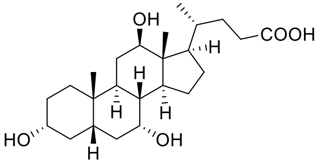

6

^{1}H NMR (500 MHz, CD_3OD)

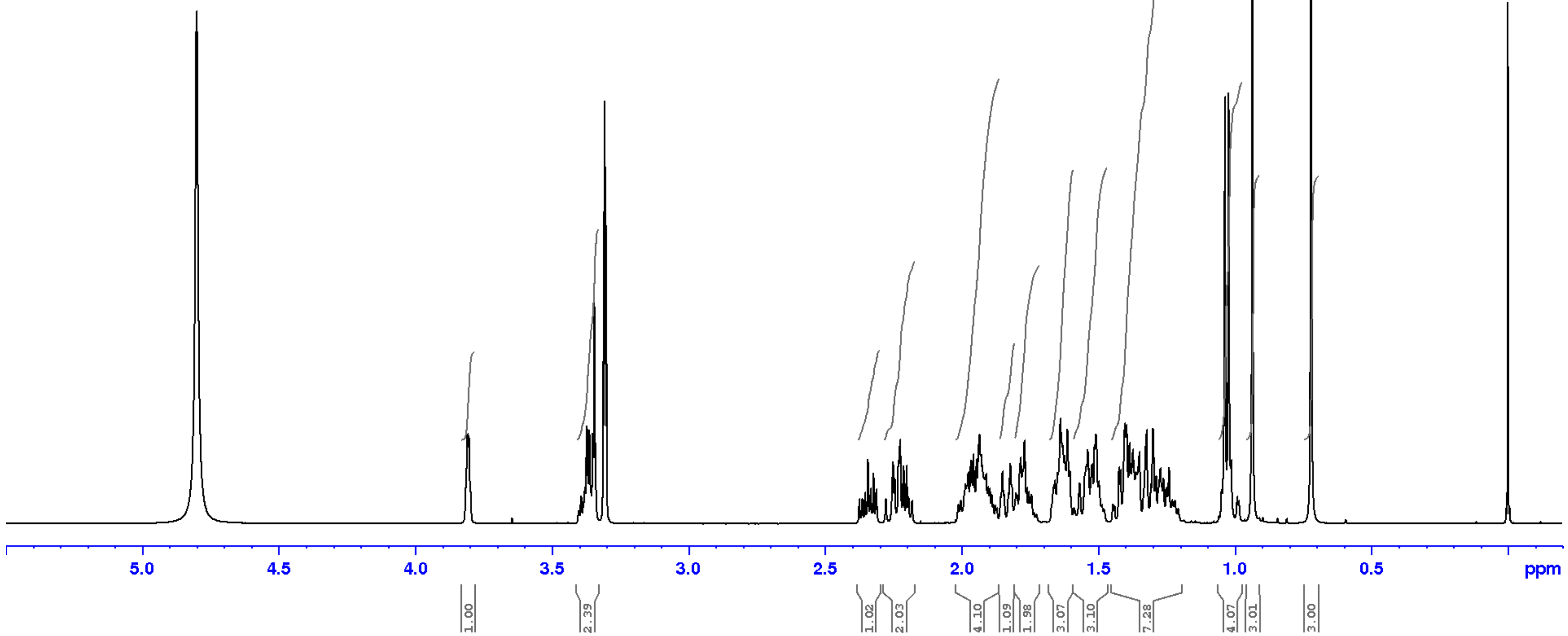

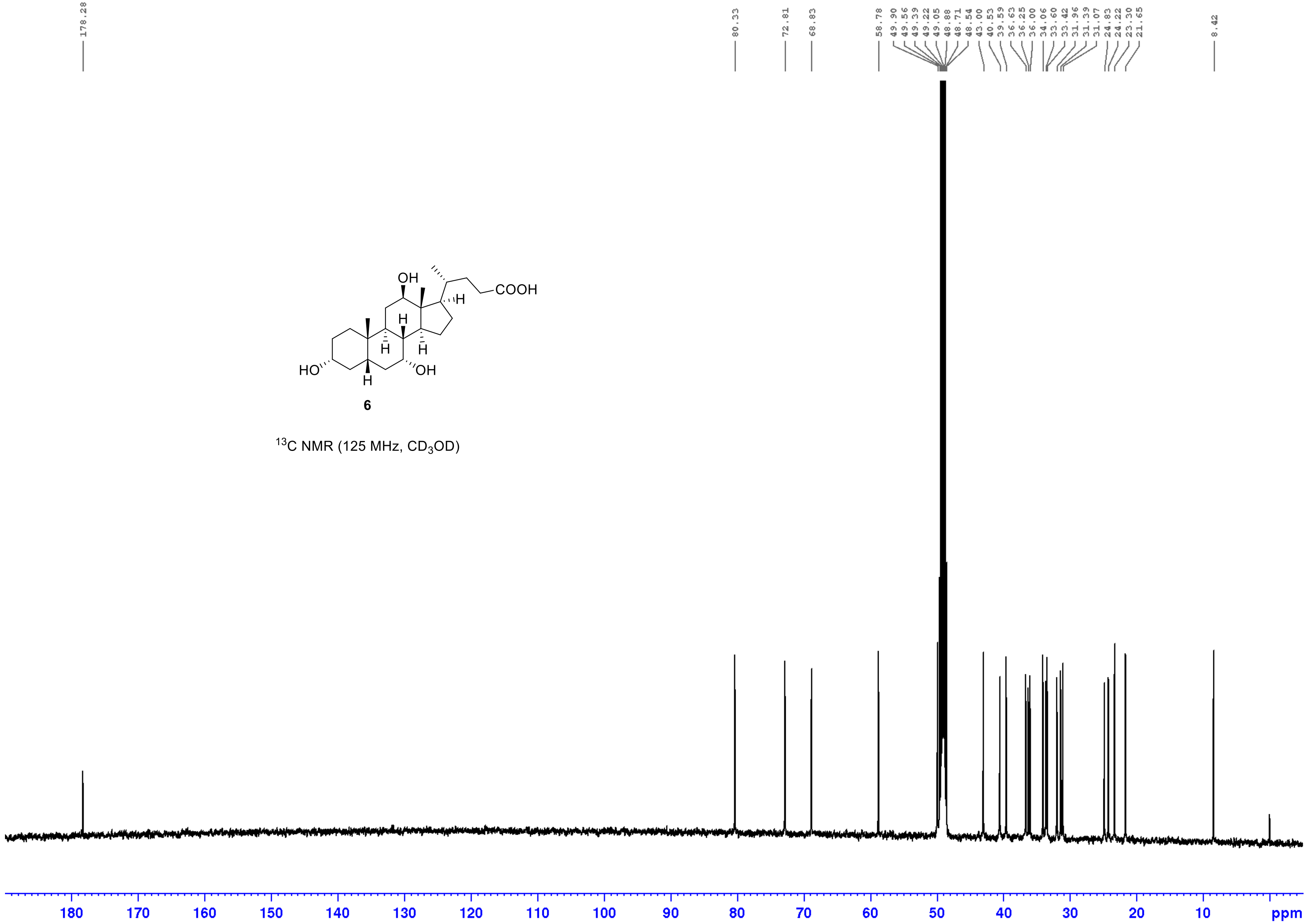

6

^{13}C NMR (125 MHz, CD_3OD)

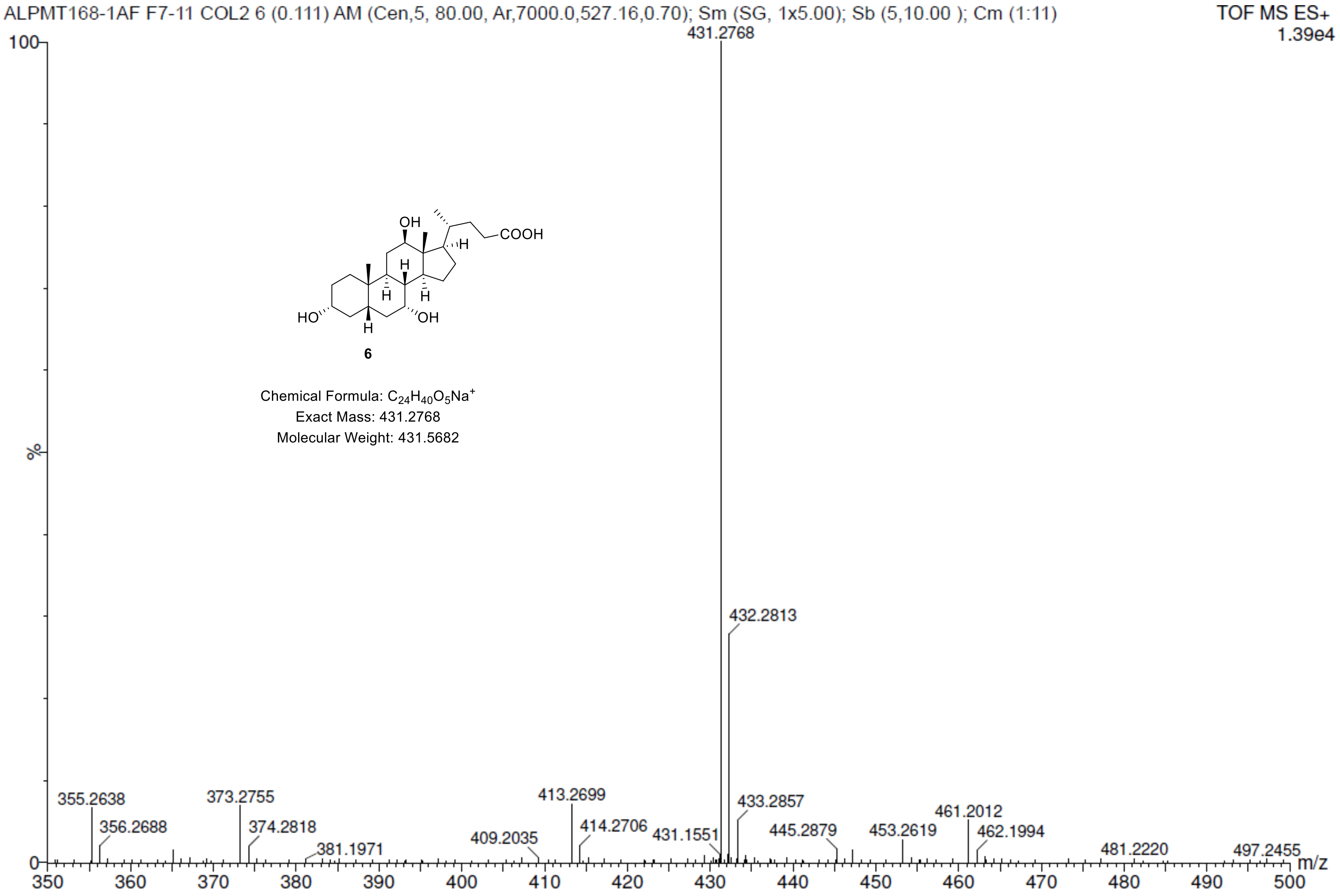
ALPMT168-1AF F7-11 COL2 6 (0.111) AM (Cen,5, 80.00, Ar,7000.0,527.16,0.70); Sm (SG, 1x5.00); Sb (5,10.00); Cm (1:11)
TOF MS ES+
1.39e4
100
%
0
431.2768
432.2813
433.2857
355.2638
356.2688
373.2755
374.2818
381.1971
409.2035
413.2699
414.2706
431.1551
445.2879
453.2619
461.2012
462.1994
481.2220
497.2455
m/z
350
360
370
380
390
400
410
420
430
440
450
460
470
480
490
500
OH
COOH
H
HO
OH
6
Chemical Formula: $C_{24}H_{40}O_5Na^+$
Exact Mass: 431.2768
Molecular Weight: 431.5682

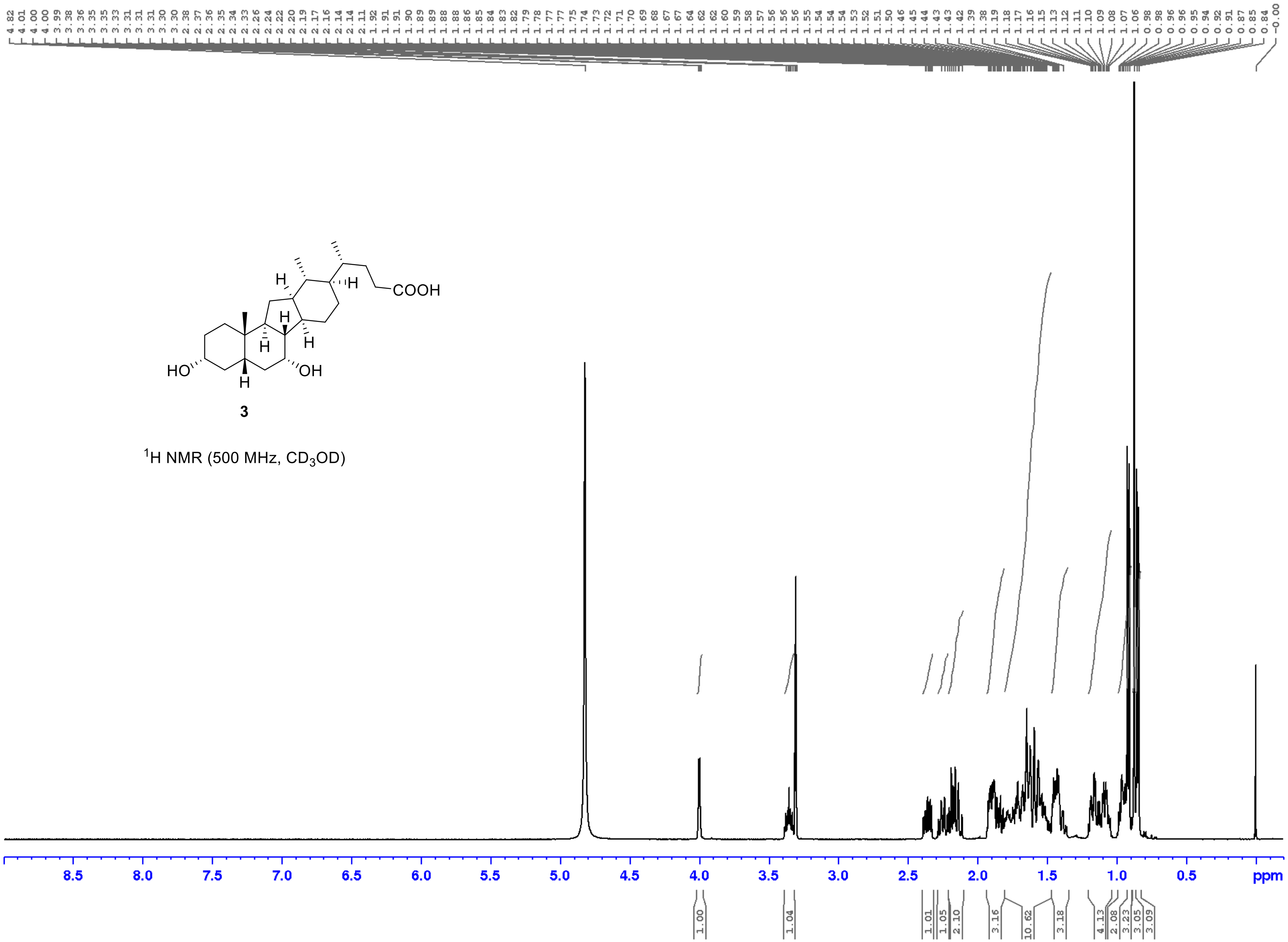
3
^{1}H NMR (500 MHz, CD$_3$OD)
ppm

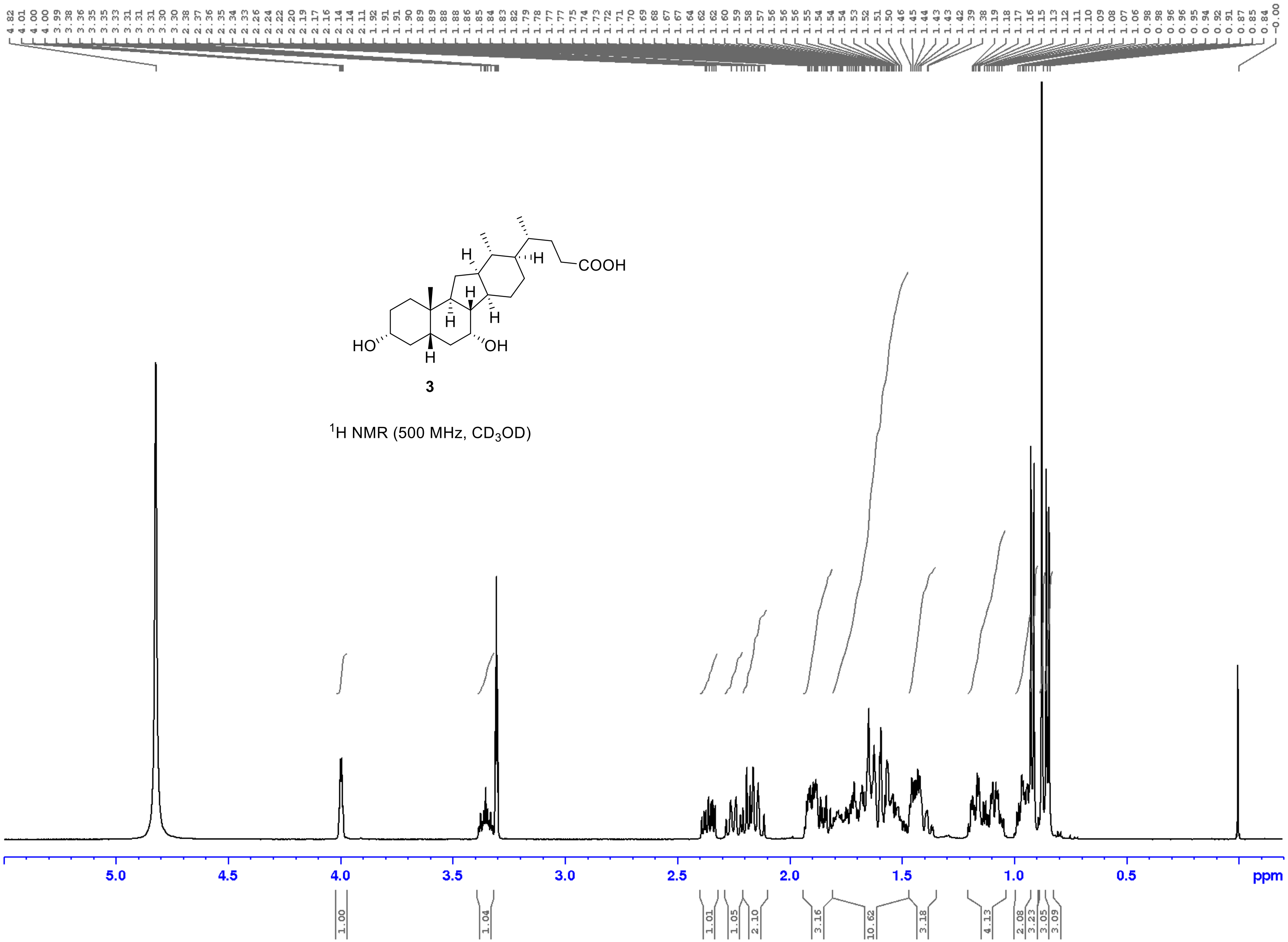
COOH
HO
OH
H
3
^{1}H NMR (500 MHz, CD$_3$OD)
5.0
4.5
4.0
3.5
3.0
2.5
2.0
1.5
1.0
0.5
ppm
1.00
1.04
1.01
1.05
2.10
3.16
10.62
3.18
4.13
2.08
3.23
3.05
3.09

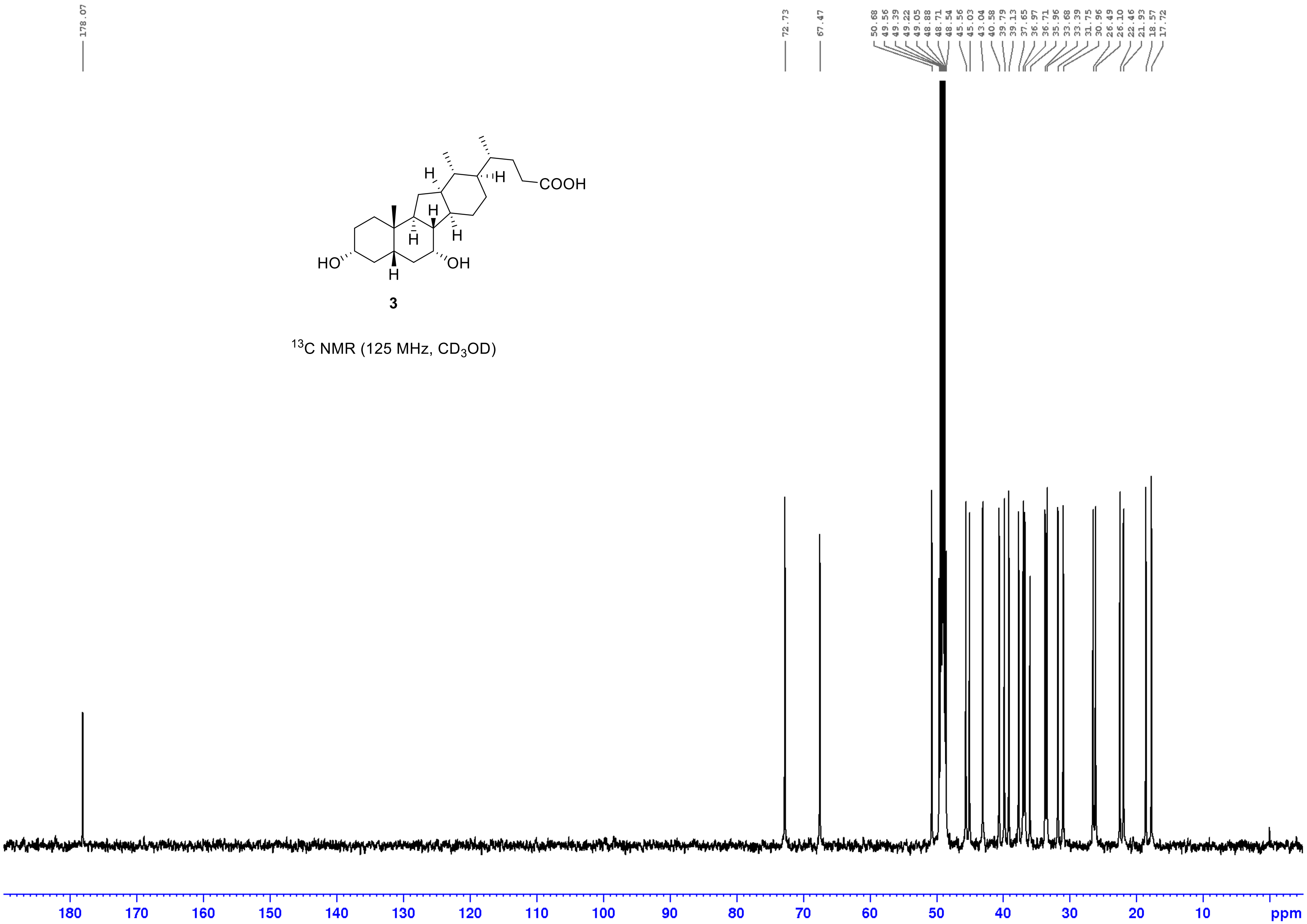

3

^{13}C NMR (125 MHz, CD_3OD)

ALPMT174AF COL2 7 (0.129) AM (Cen,5, 80.00, Ar,7000.0,527.16,0.70); Sm (SG, 1x5.00); Sb (5,10.00); Cm (1:11) TOF MS ES+ 1.18e4

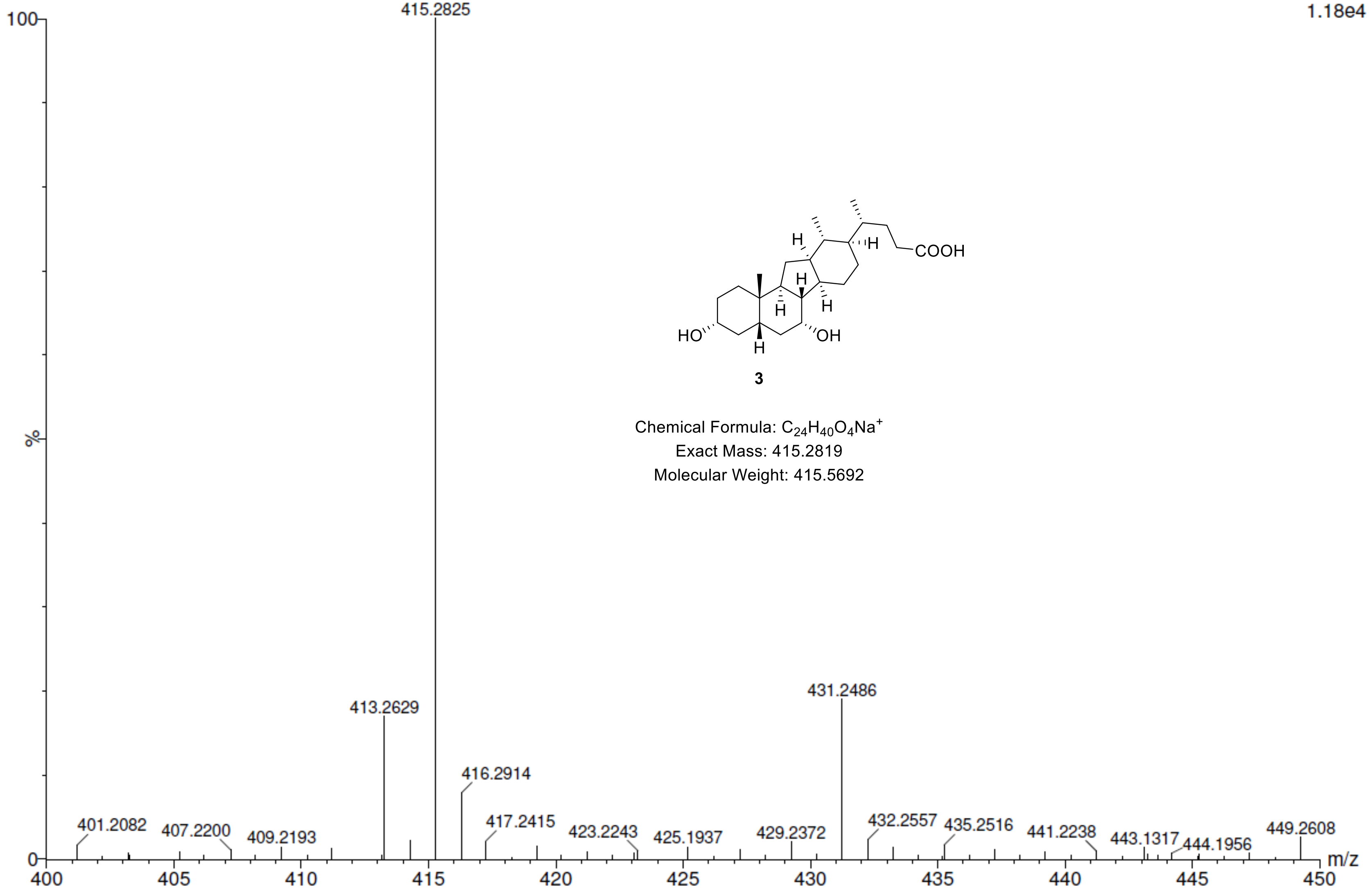

3

Chemical Formula: $C_{24}H_{40}O_4Na^+$
Exact Mass: 415.2819
Molecular Weight: 415.5692

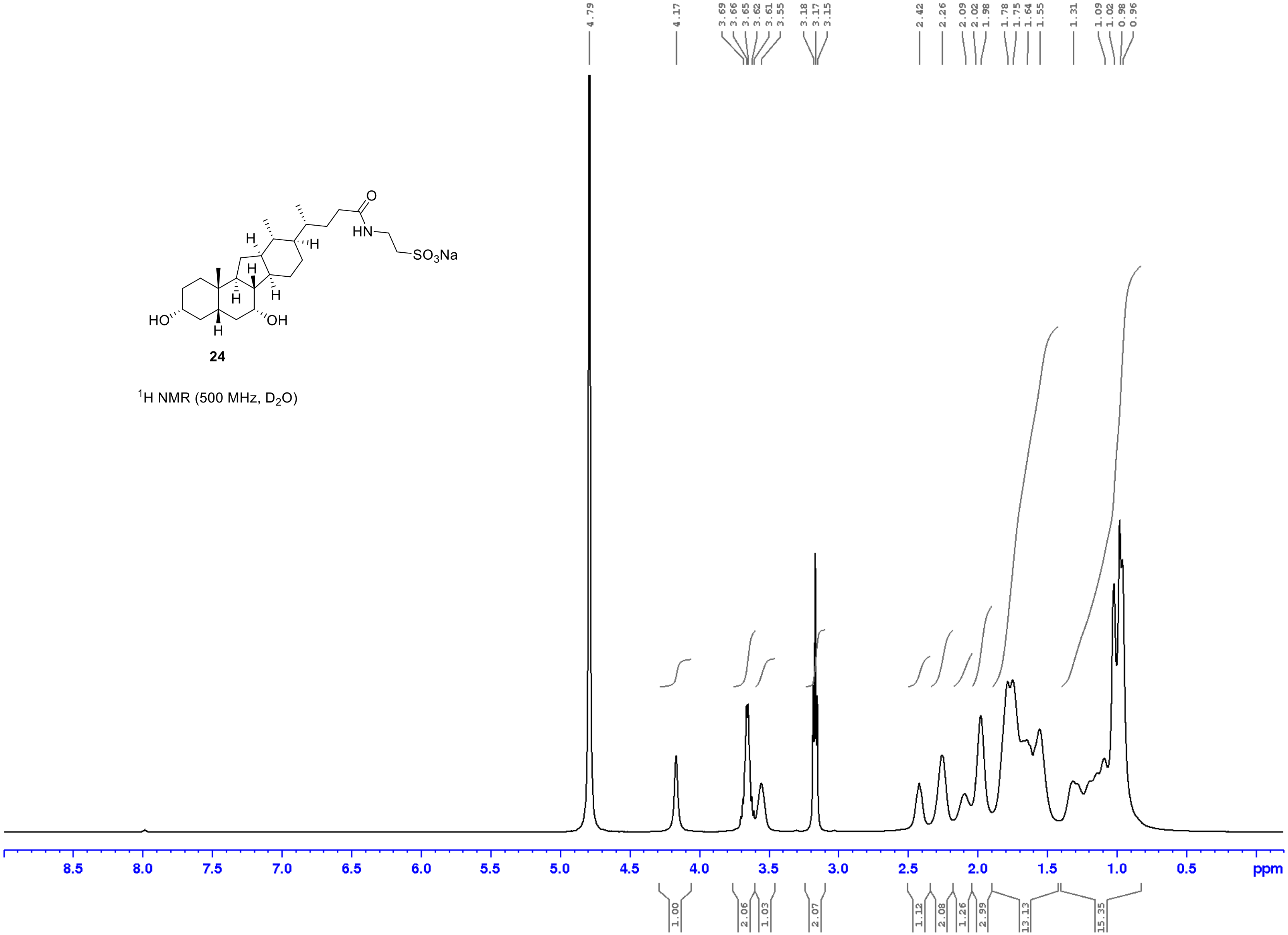

24

^{1}H NMR (500 MHz, D_2O)

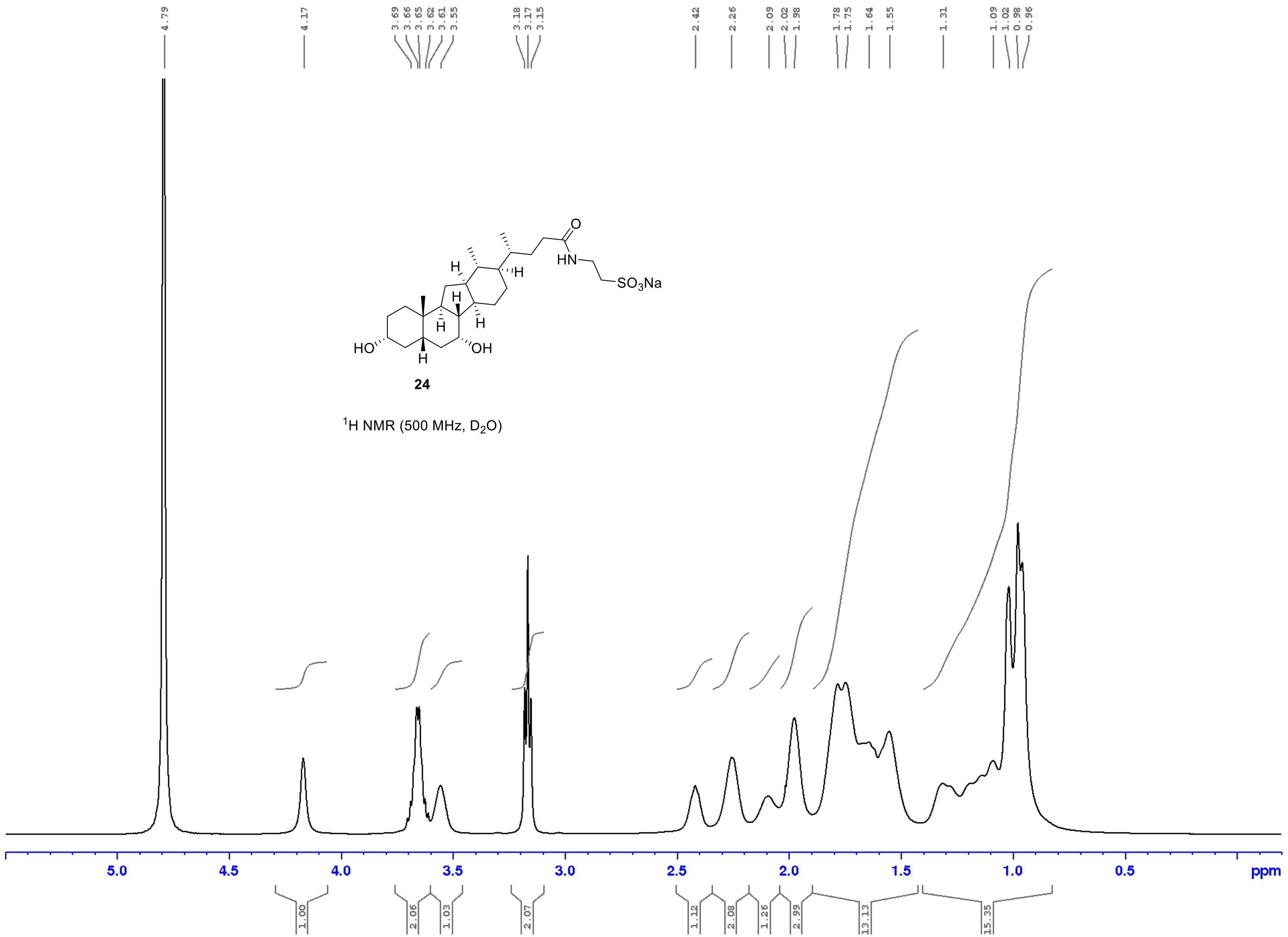
4.79
4.17
3.69
3.66
3.65
3.62
3.61
3.55
3.18
3.17
3.15
2.42
2.26
2.09
2.02
1.98
1.78
1.75
1.64
1.55
1.31
1.09
1.02
0.98
0.96
HO
OH
H
HN
O
SO_3Na
24
^{1}H NMR (500 MHz, D_2O)
5.0
4.5
4.0
3.5
3.0
2.5
2.0
1.5
1.0
0.5
ppm
1.00
2.06
1.03
2.07
1.12
2.08
1.26
2.99
13.13
15.35

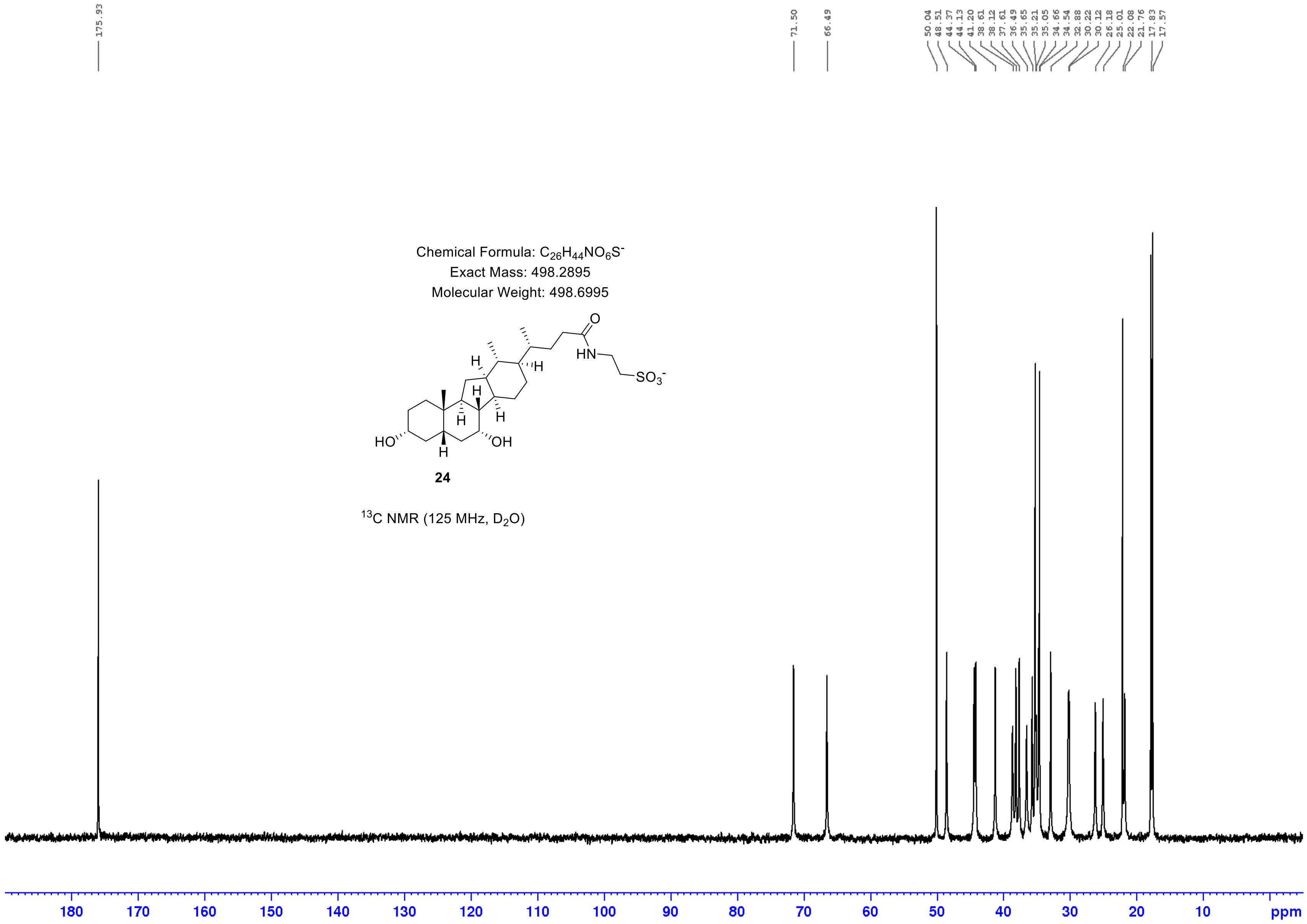
175.93
71.50
66.49
50.04
48.51
44.37
44.13
41.20
38.61
38.12
37.61
36.49
35.65
35.21
35.05
34.66
34.54
32.88
30.22
30.12
26.18
25.01
22.08
21.76
17.83
17.57
Chemical Formula: $C_{26}H_{44}NO_6S^-$
Exact Mass: 498.2895
Molecular Weight: 498.6995
O
HN
SO_3^-
H
H
H
H
H
HO
OH
H
24
^{13}C NMR (125 MHz, D_2O)
180
170
160
150
140
130
120
110
100
90
80
70
60
50
40
30
20
10
ppm

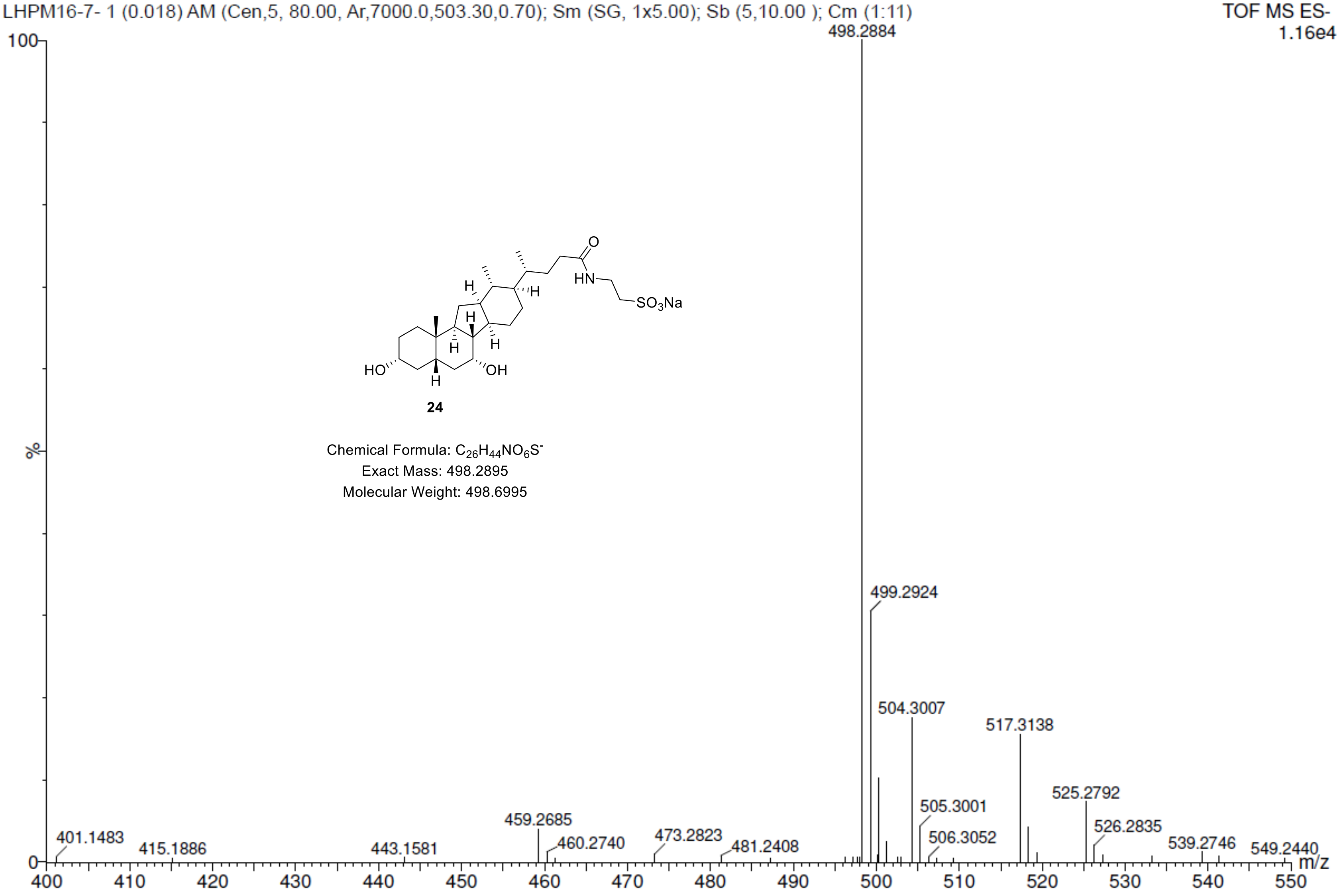
LHPM16-7- 1 (0.018) AM (Cen,5, 80.00, Ar,7000.0,503.30,0.70); Sm (SG, 1x5.00); Sb (5,10.00); Cm (1:11)
TOF MS ES-
1.16e4
100
%
0
498.2884
499.2924
504.3007
517.3138
525.2792
505.3001
526.2835
506.3052
401.1483
415.1886
443.1581
459.2685
460.2740
473.2823
481.2408
539.2746
549.2440
m/z
400
410
420
430
440
450
460
470
480
490
500
510
520
530
540
550
O
HN
SO_3Na
H
H
H
H
H
HO
OH
H
24
Chemical Formula: $C_{26}H_{44}NO_6S^-$
Exact Mass: 498.2895
Molecular Weight: 498.6995

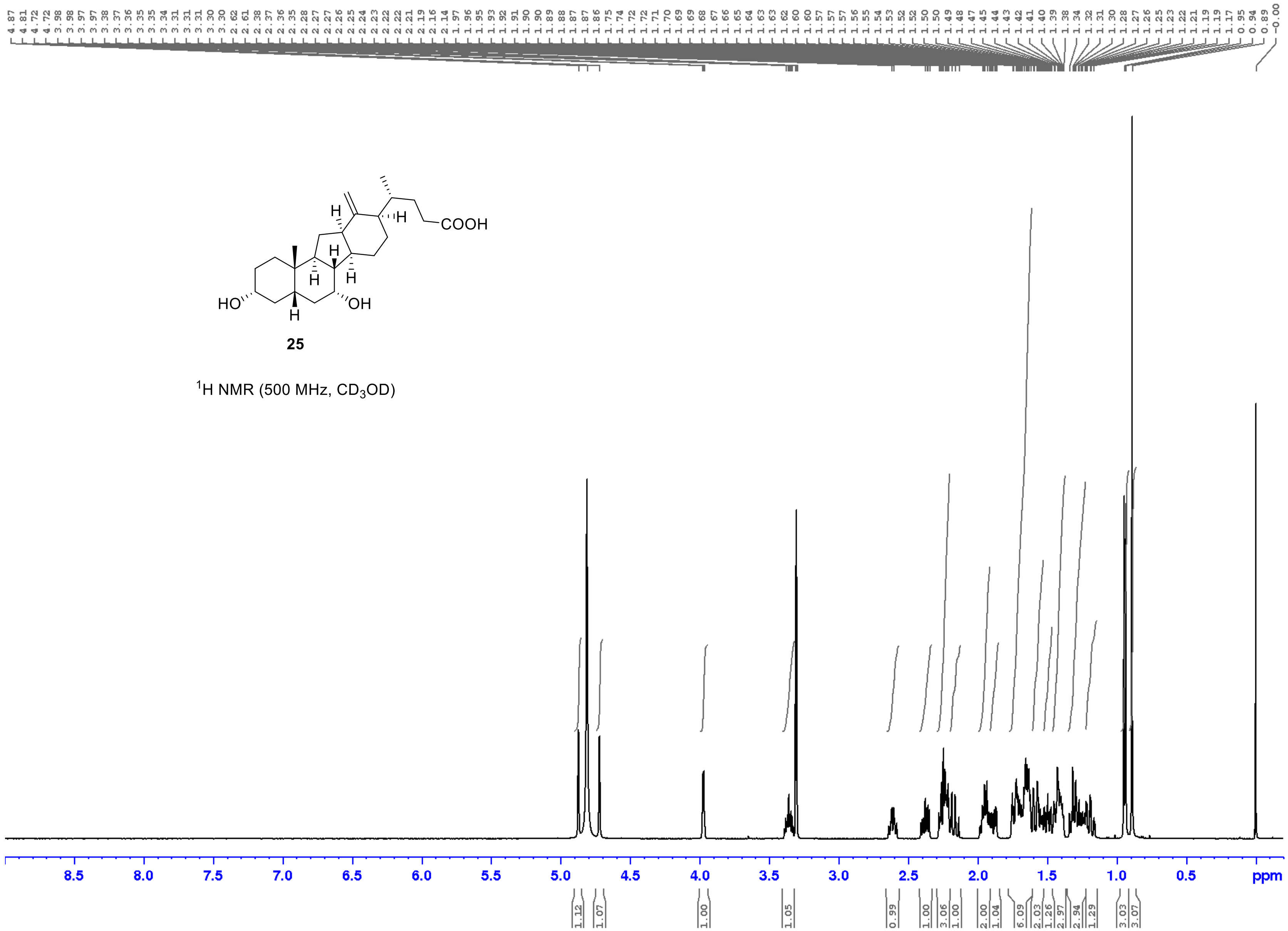

25

^1H NMR (500 MHz, CD_3OD)

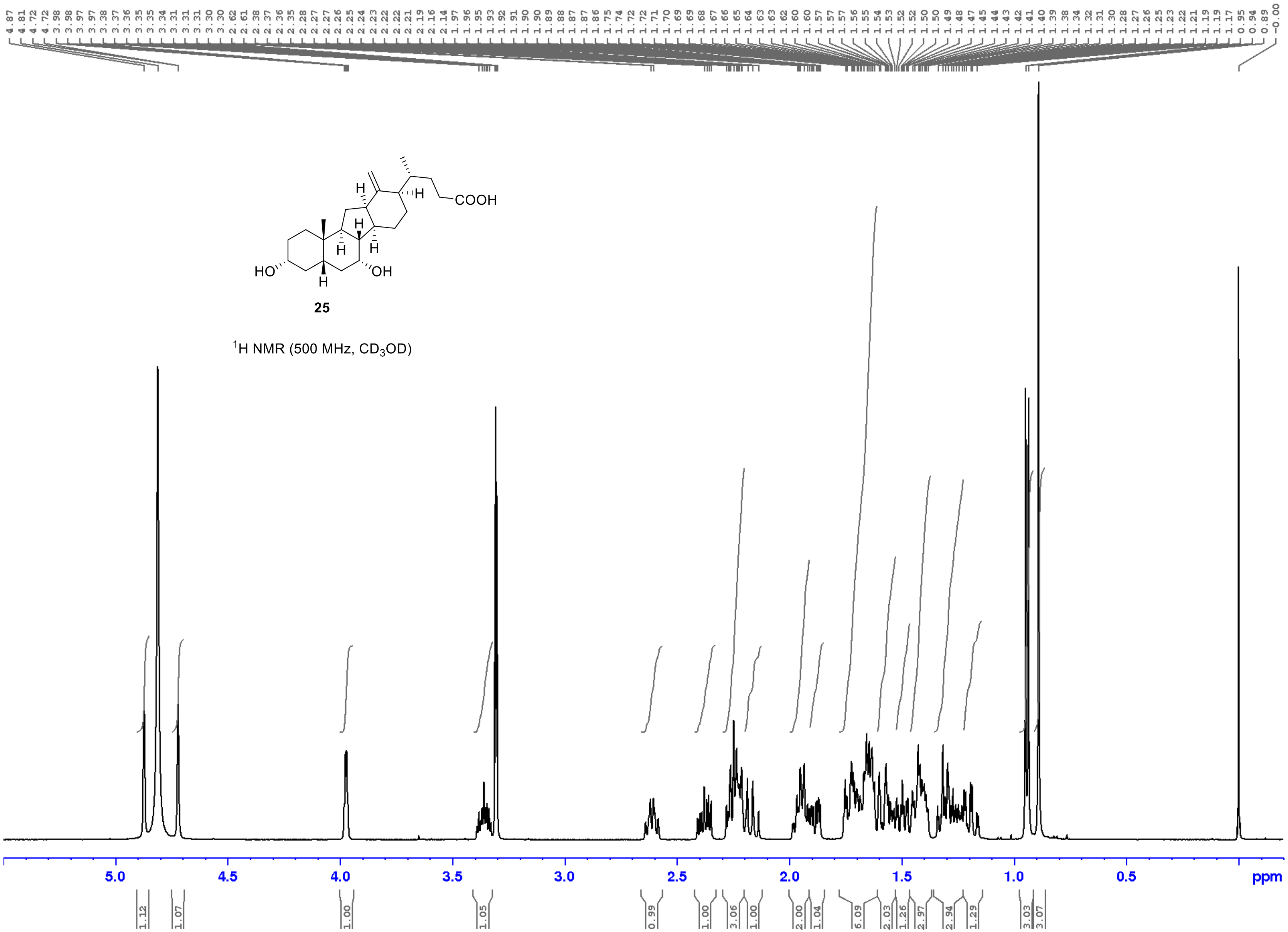
COOH
HO
OH
25
^{1}H NMR (500 MHz, CD_3OD)
5.0
4.5
4.0
3.5
3.0
2.5
2.0
1.5
1.0
0.5
ppm

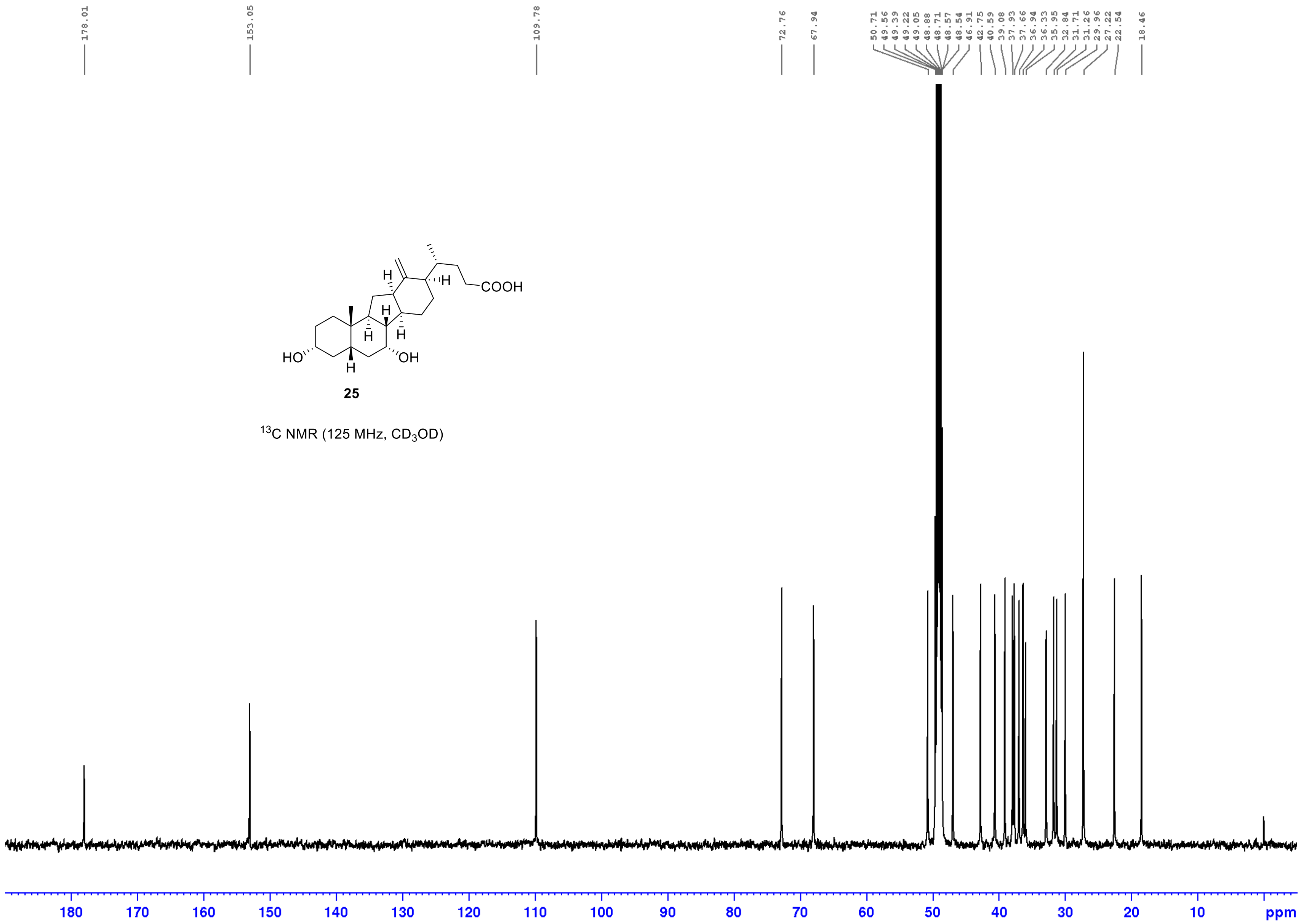

178.01
153.05
109.78
72.76
67.94
50.71
49.56
49.39
49.22
49.05
48.88
48.71
48.57
48.54
46.91
42.75
40.59
39.08
37.93
37.66
36.94
36.33
35.95
32.84
31.71
31.26
29.96
27.22
22.54
18.46
COOH
HO
OH
25
^{13}C NMR (125 MHz, CD_3OD)
180
170
160
150
140
130
120
110
100
90
80
70
60
50
40
30
20
10
ppm

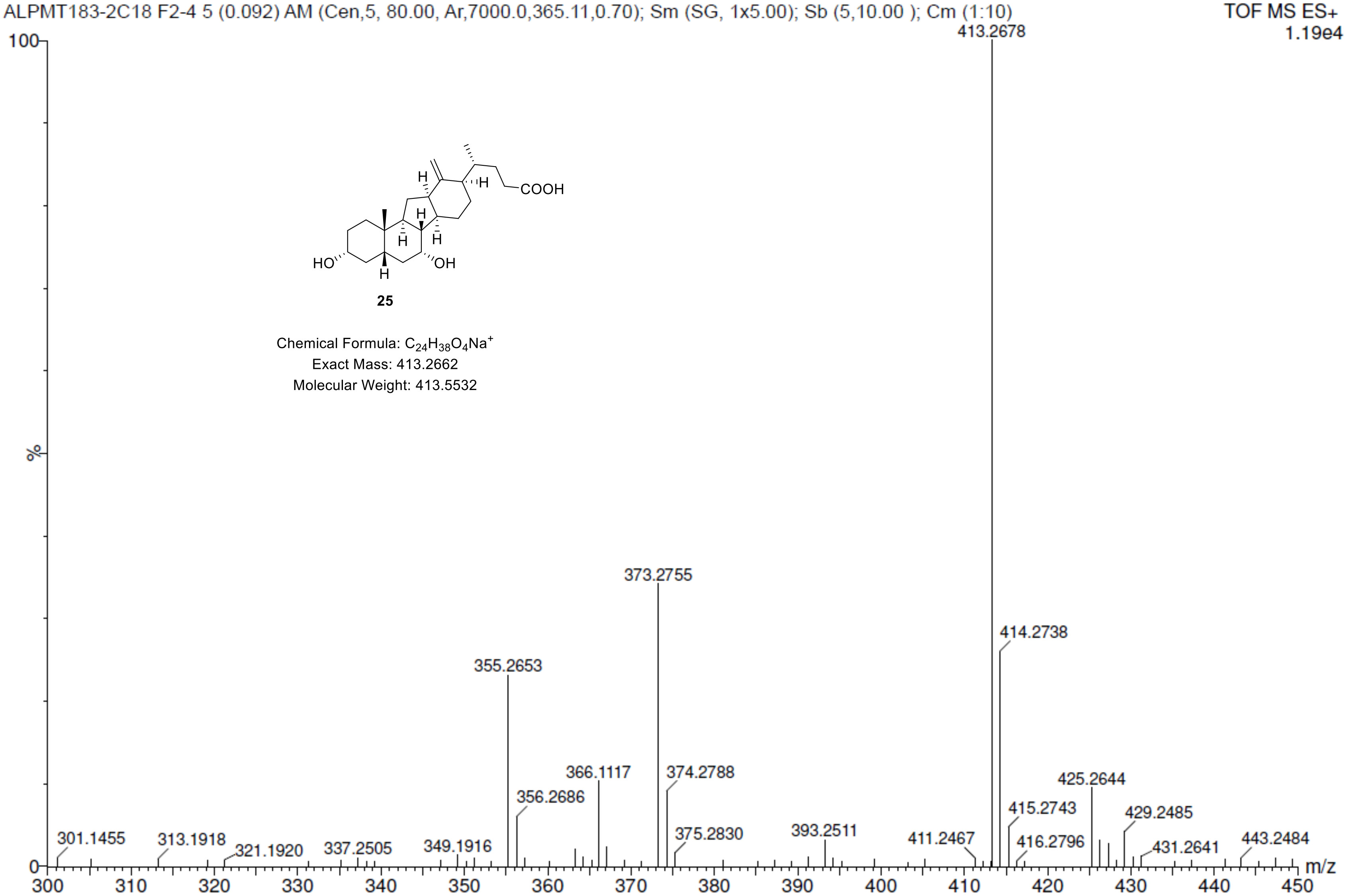
ALPMT183-2C18 F2-4 5 (0.092) AM (Cen,5, 80.00, Ar,7000.0,365.11,0.70); Sm (SG, 1x5.00); Sb (5,10.00); Cm (1:10)
TOF MS ES+
1.19e4
100
%
0
413.2678
414.2738
373.2755
355.2653
366.1117
374.2788
356.2686
425.2644
415.2743
429.2485
375.2830
393.2511
411.2467
416.2796
431.2641
443.2484
301.1455
313.1918
321.1920
337.2505
349.1916
300
310
320
330
340
350
360
370
380
390
400
410
420
430
440
450
m/z
H
H
COOH
H
H
H
HO
OH
H
25
Chemical Formula: C24H38O4Na+
Exact Mass: 413.2662
Molecular Weight: 413.5532

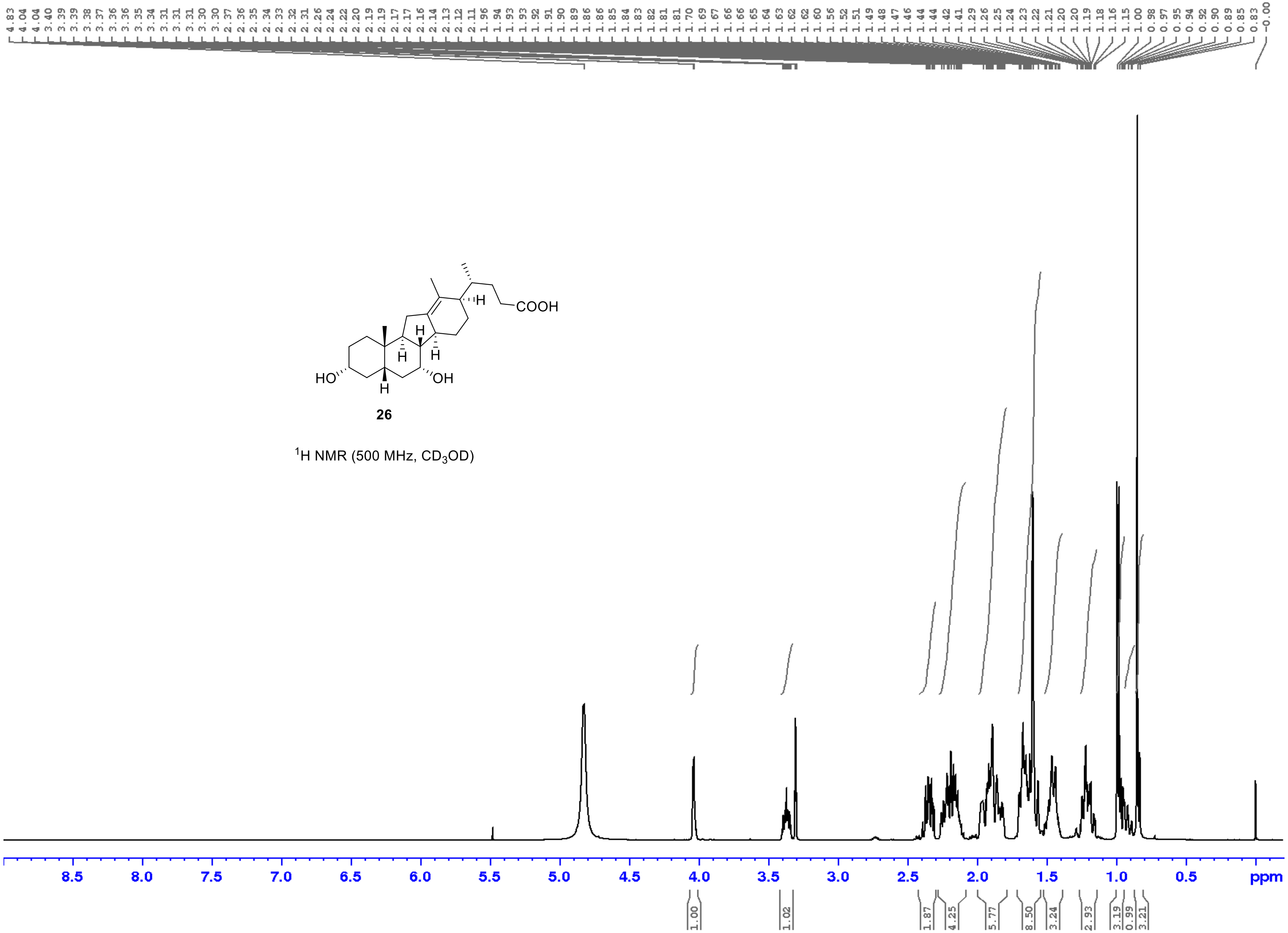

26

^{1}H NMR (500 MHz, CD_3OD)

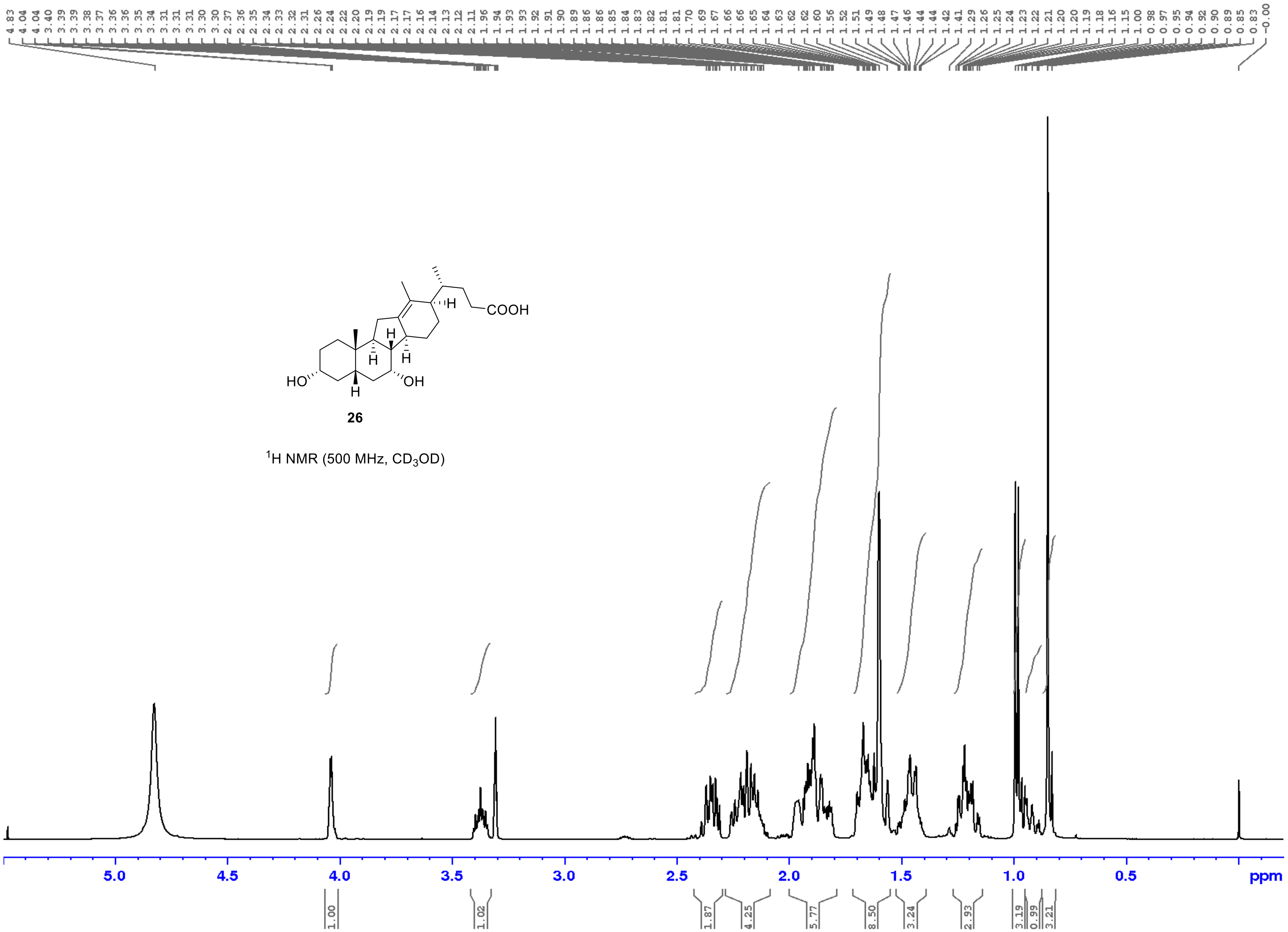
4.83 4.04 4.04 3.40 3.39 3.39 3.38 3.37 3.36 3.36 3.35 3.34 3.31 3.31 3.31 3.30 3.30 2.37 2.36 2.35 2.34 2.33 2.32 2.31 2.26 2.24 2.22 2.20 2.19 2.19 2.17 2.17 2.16 2.14 2.13 2.12 2.11 1.96 1.94 1.93 1.93 1.92 1.91 1.90 1.89 1.86 1.86 1.85 1.84 1.83 1.82 1.81 1.81 1.70 1.69 1.67 1.66 1.65 1.64 1.63 1.62 1.62 1.60 1.56 1.52 1.51 1.49 1.48 1.47 1.46 1.44 1.44 1.42 1.41 1.29 1.26 1.25 1.24 1.23 1.22 1.21 1.21 1.20 1.20 1.19 1.18 1.16 1.15 1.00 0.98 0.97 0.95 0.94 0.92 0.90 0.89 0.85 0.83 -0.00
COOH
H
HO
OH
26
1H NMR (500 MHz, CD3OD)
5.0
4.5
4.0
3.5
3.0
2.5
2.0
1.5
1.0
0.5
ppm
1.00
1.02
1.87
4.25
5.77
8.50
3.24
2.93
3.19
0.99
3.21

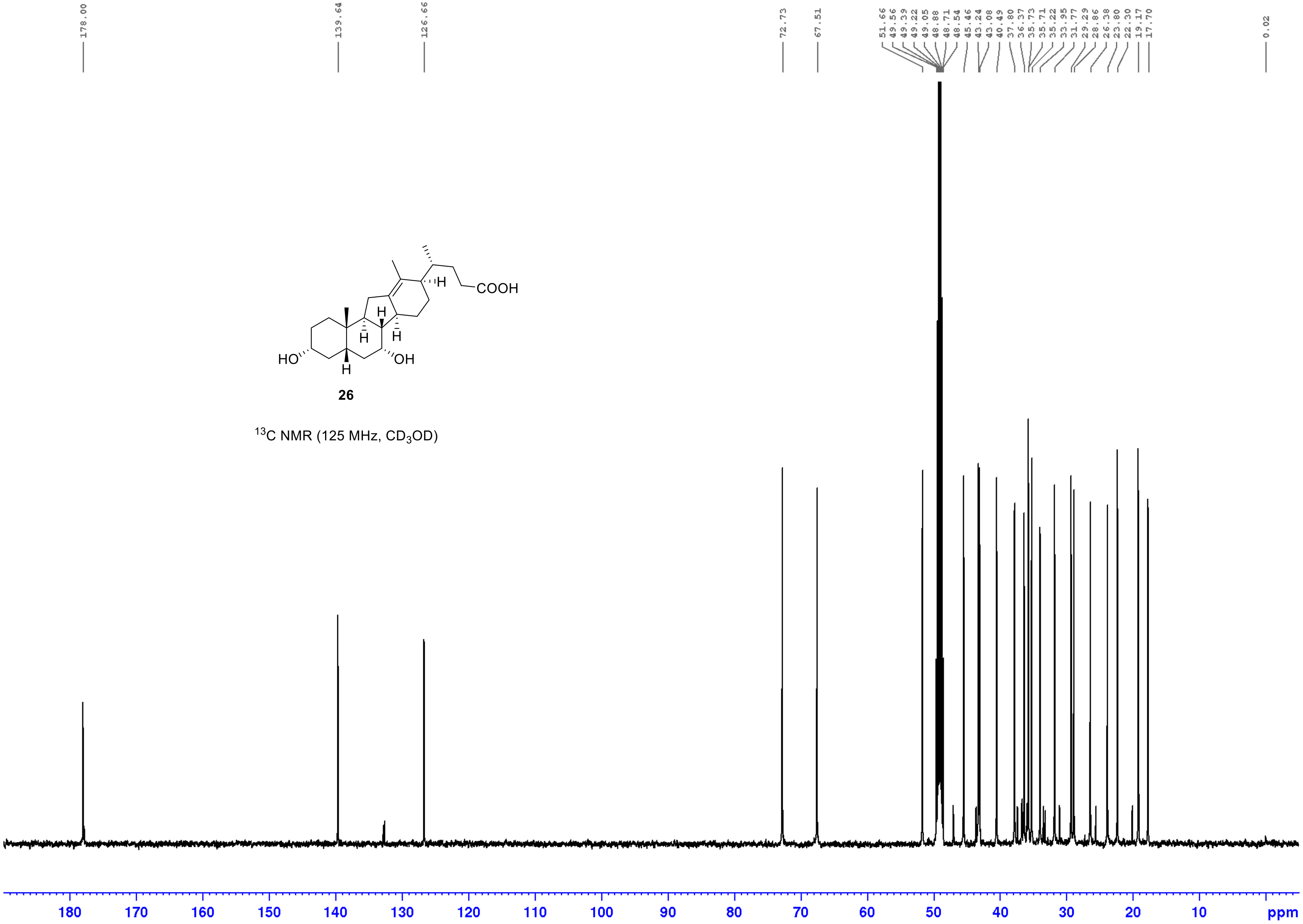
178.00
139.64
126.66
72.73
67.51
51.66
49.56
49.39
49.22
49.05
48.88
48.71
48.54
45.46
43.24
43.08
40.49
37.80
36.37
35.73
35.71
35.22
33.95
31.77
29.29
28.86
26.38
23.80
22.30
19.17
17.70
0.02
H
COOH
H
H
H
HO
H
OH
26
^{13}C NMR (125 MHz, CD_3OD)
180
170
160
150
140
130
120
110
100
90
80
70
60
50
40
30
20
10
ppm

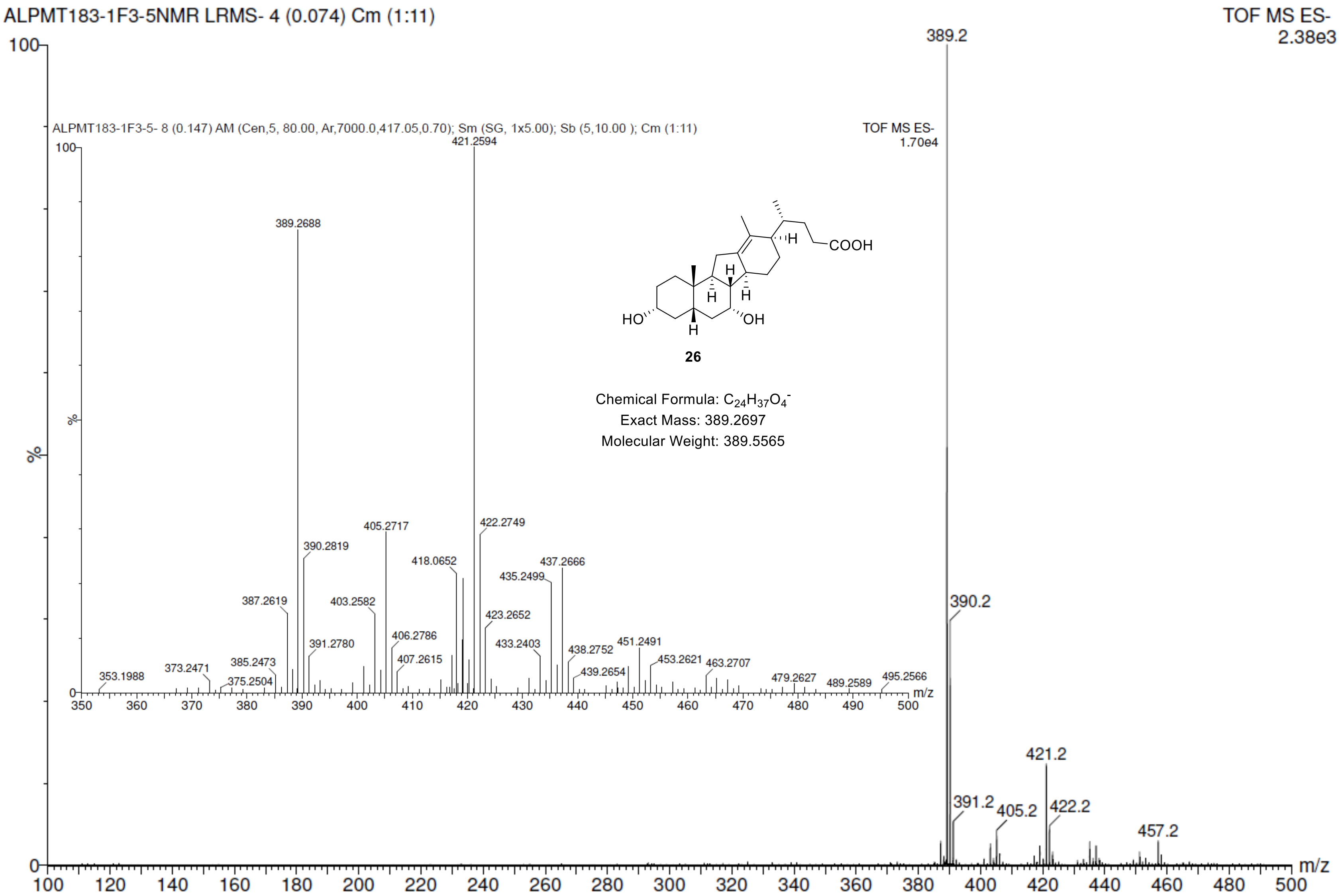

ALPMT183-1F3-5NMR LRMS- 4 (0.074) Cm (1:11)
TOF MS ES-
2.38e3
389.2
390.2
391.2
405.2
421.2
422.2
457.2
ALPMT183-1F3-5- 8 (0.147) AM (Cen,5, 80.00, Ar,7000.0,417.05,0.70); Sm (SG, 1x5.00); Sb (5,10.00); Cm (1:11)
TOF MS ES-
1.70e4
421.2594
389.2688
405.2717
422.2749
390.2819
418.0652
437.2666
435.2499
387.2619
403.2582
423.2652
406.2786
391.2780
433.2403
451.2491
438.2752
407.2615
453.2621
463.2707
385.2473
373.2471
439.2654
353.1988
375.2504
479.2627
489.2589
495.2566
HO
OH
H
COOH
26
Chemical Formula: $C_{24}H_{37}O_4^-$
Exact Mass: 389.2697
Molecular Weight: 389.5565
%
m/z

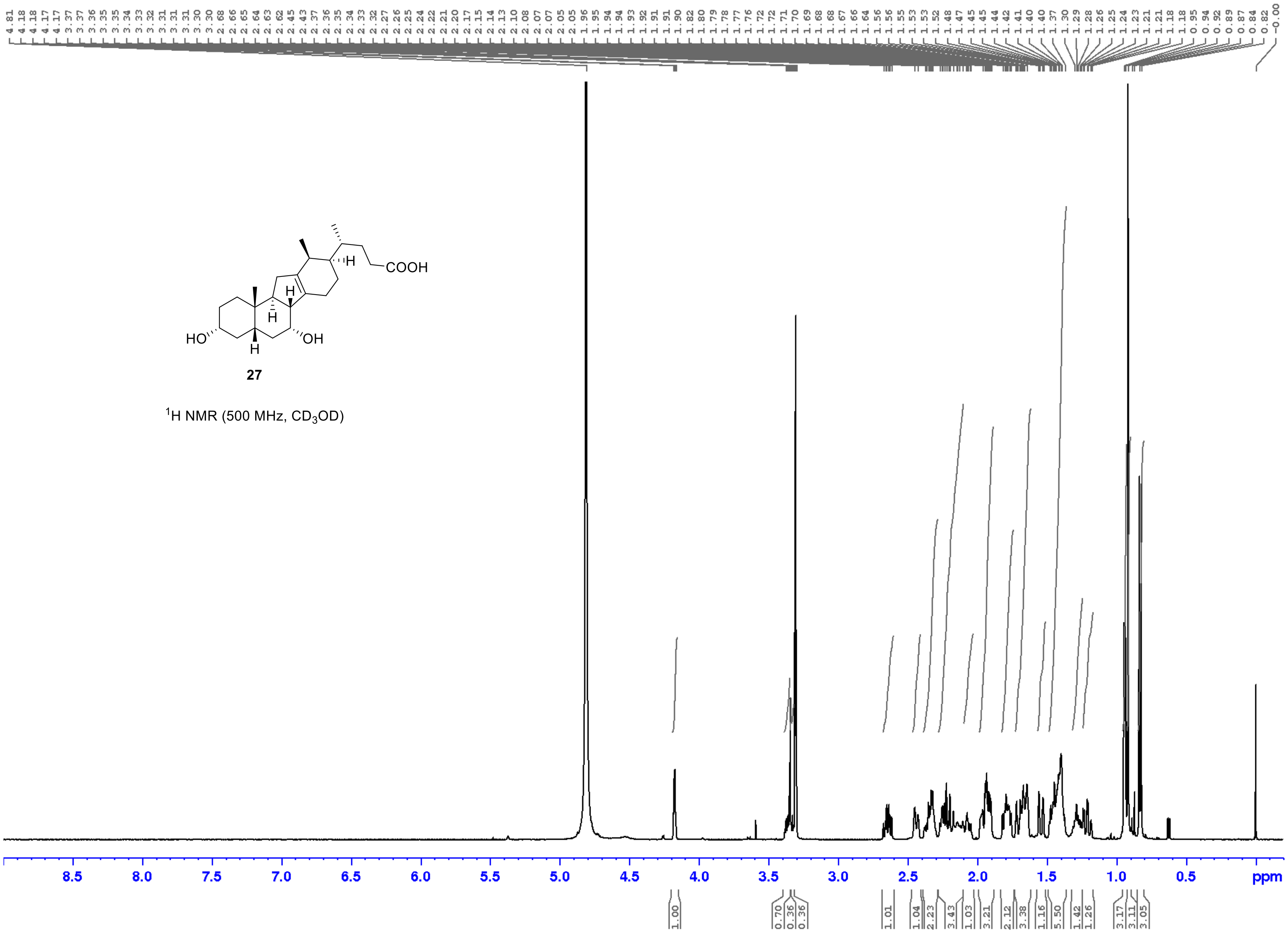
COOH
HO
OH
H
27
^{1}H NMR (500 MHz, CD$_3$OD)
ppm

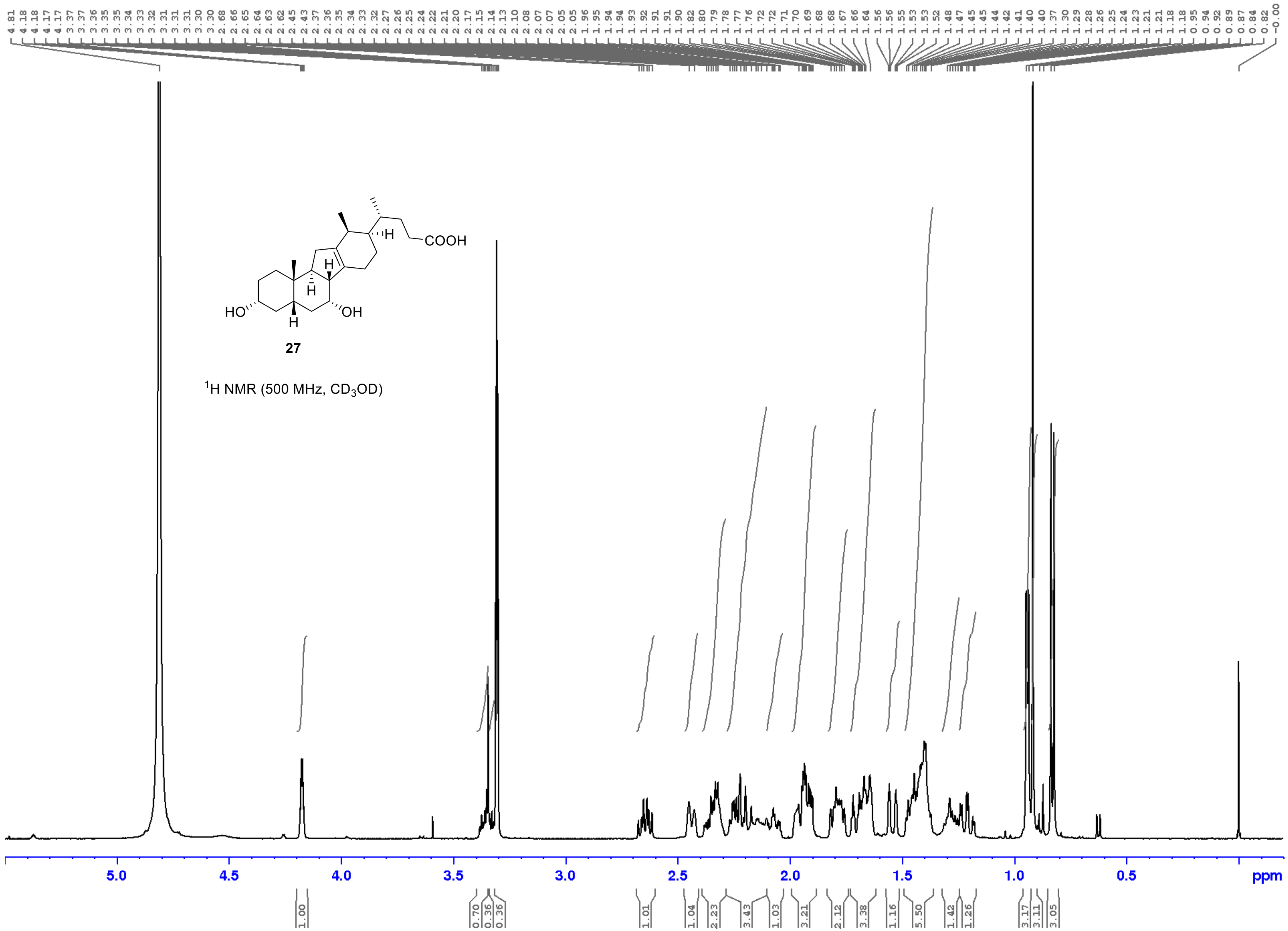

27

^{1}H NMR (500 MHz, CD_3OD)

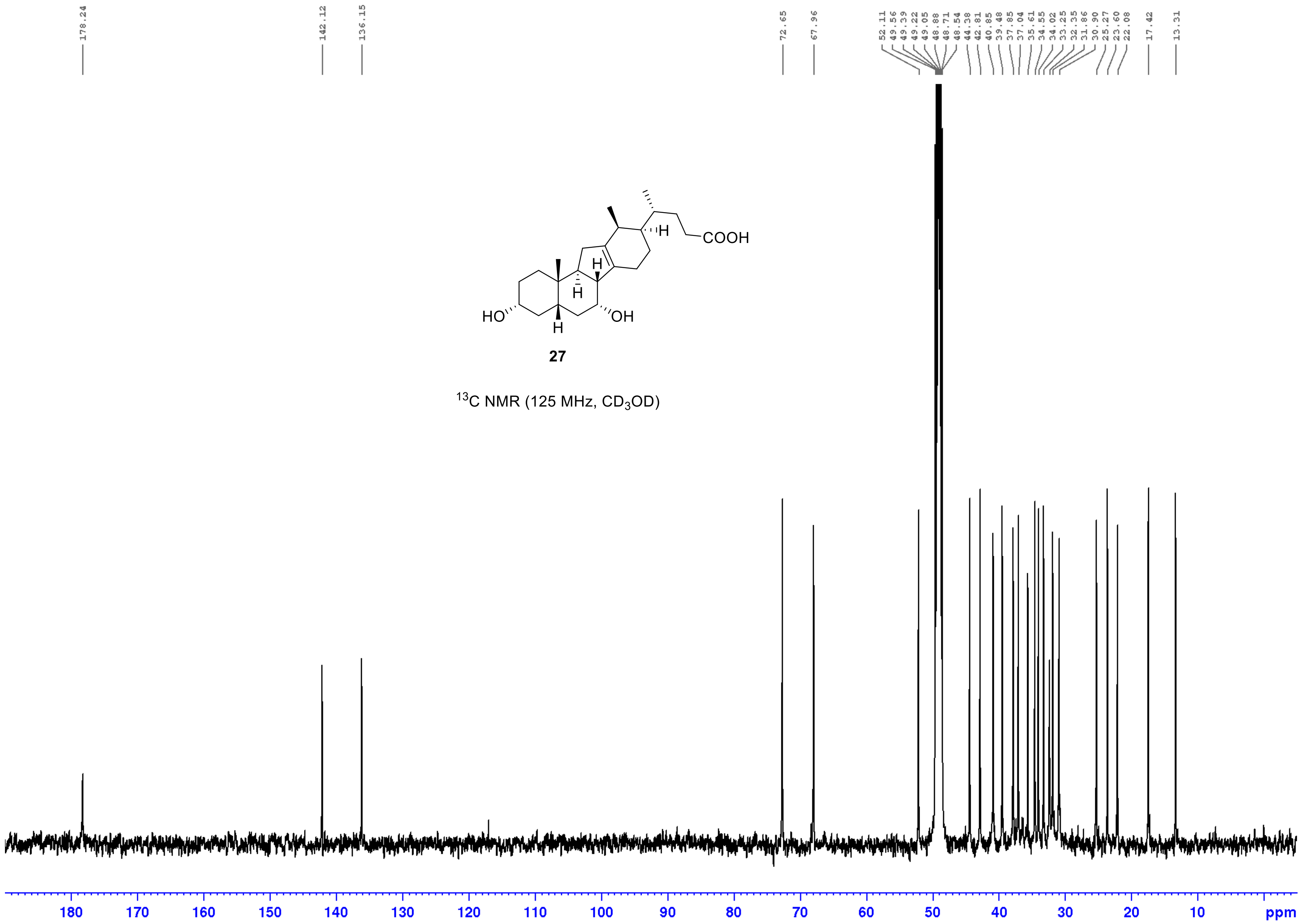
178.24
142.12
136.15
72.65
67.96
52.11
49.56
49.39
49.22
49.05
48.88
48.71
48.54
44.38
42.81
40.85
39.48
37.85
37.04
35.61
34.55
34.02
33.25
32.35
31.86
30.90
25.27
23.60
22.08
17.42
13.31
COOH
H
HO
OH
27
^{13}C NMR (125 MHz, CD_3OD)
180
170
160
150
140
130
120
110
100
90
80
70
60
50
40
30
20
10
ppm

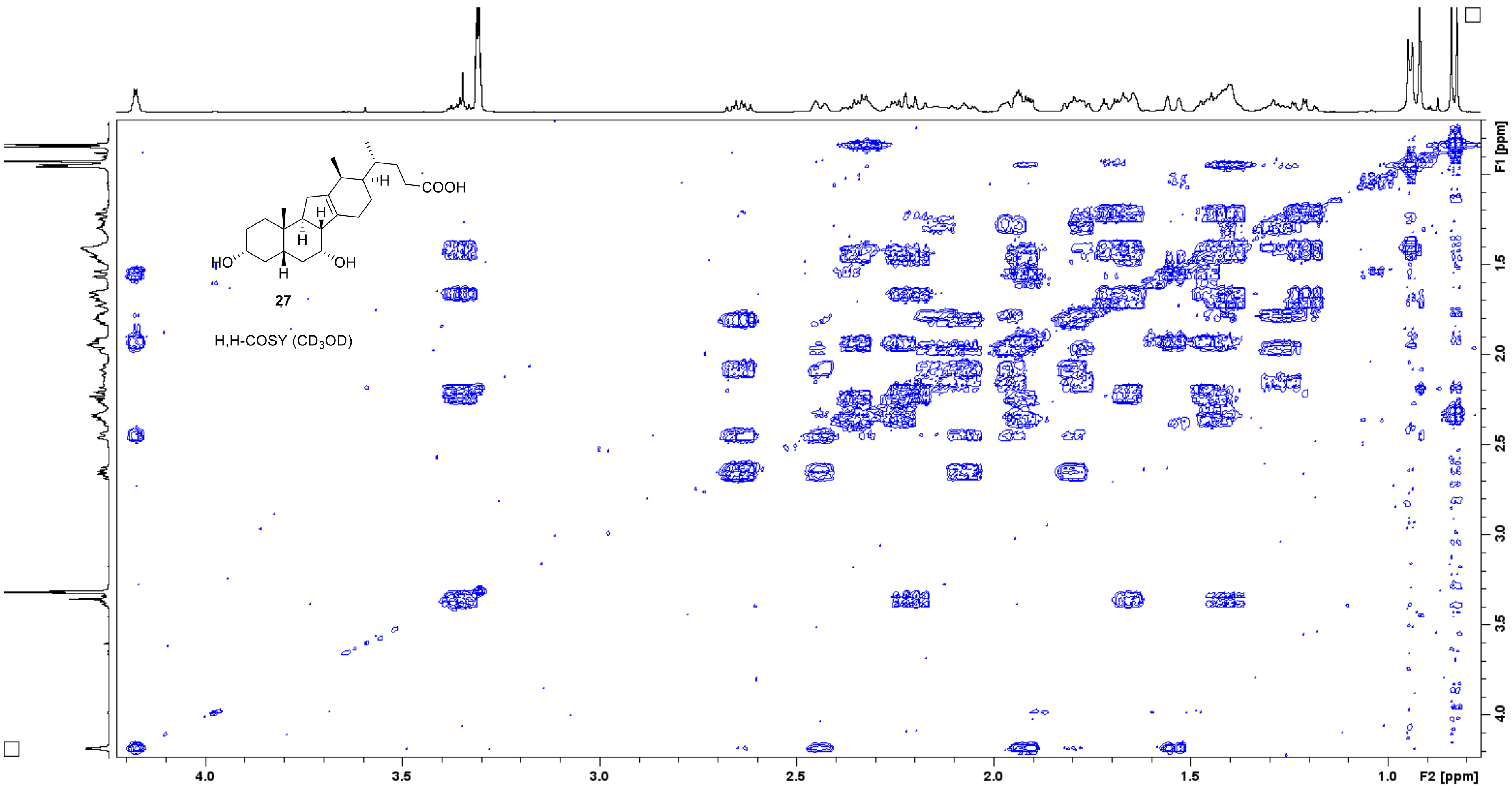
HO
OH
H
H
H
H
COOH
27
H,H-COSY (CD3OD)
F1 [ppm]
F2 [ppm]
4.0
3.5
3.0
2.5
2.0
1.5
1.0

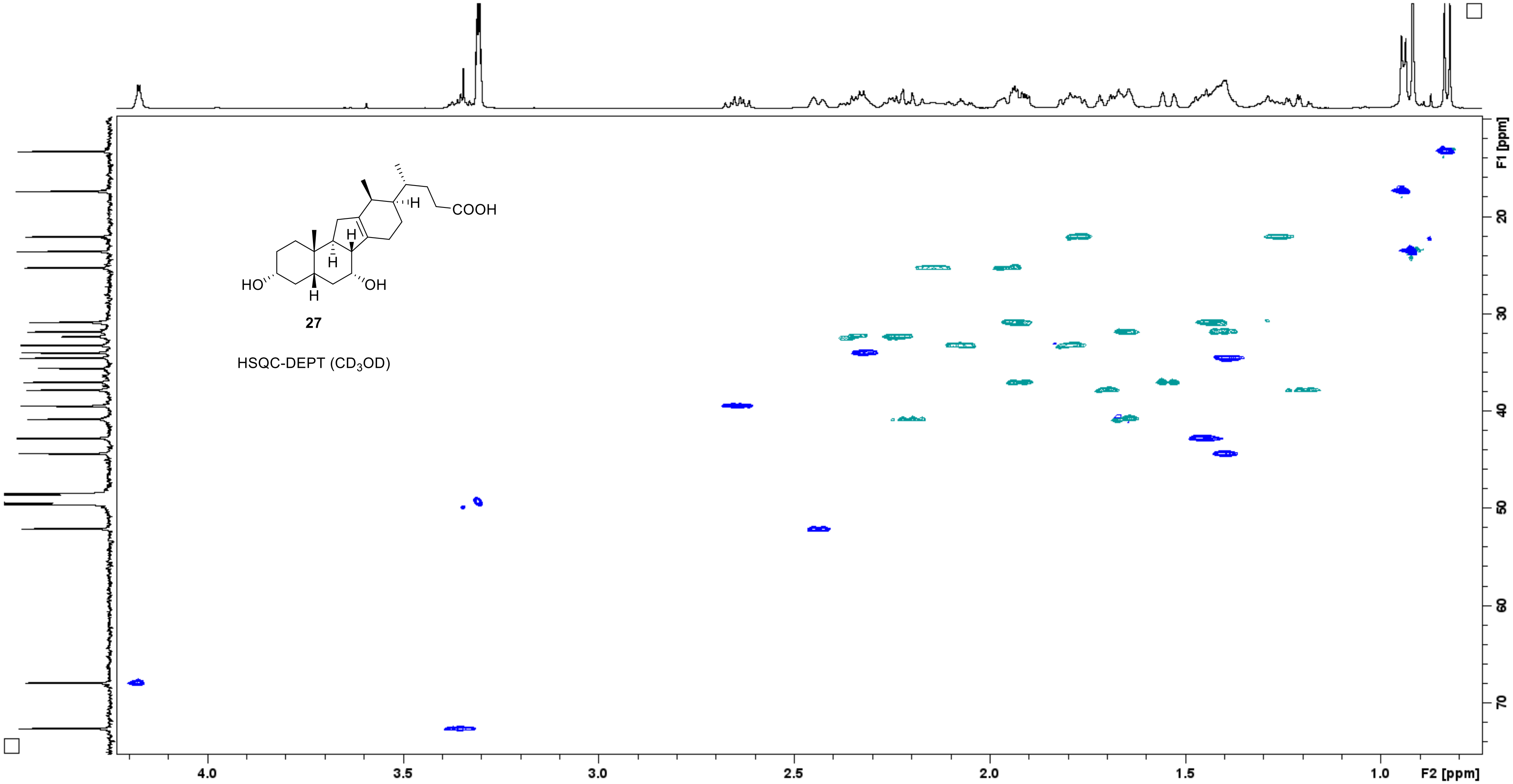
COOH
HO
OH
H
27
HSQC-DEPT (CD3OD)
F1 [ppm]
20
30
40
50
60
70
4.0
3.5
3.0
2.5
2.0
1.5
1.0
F2 [ppm]

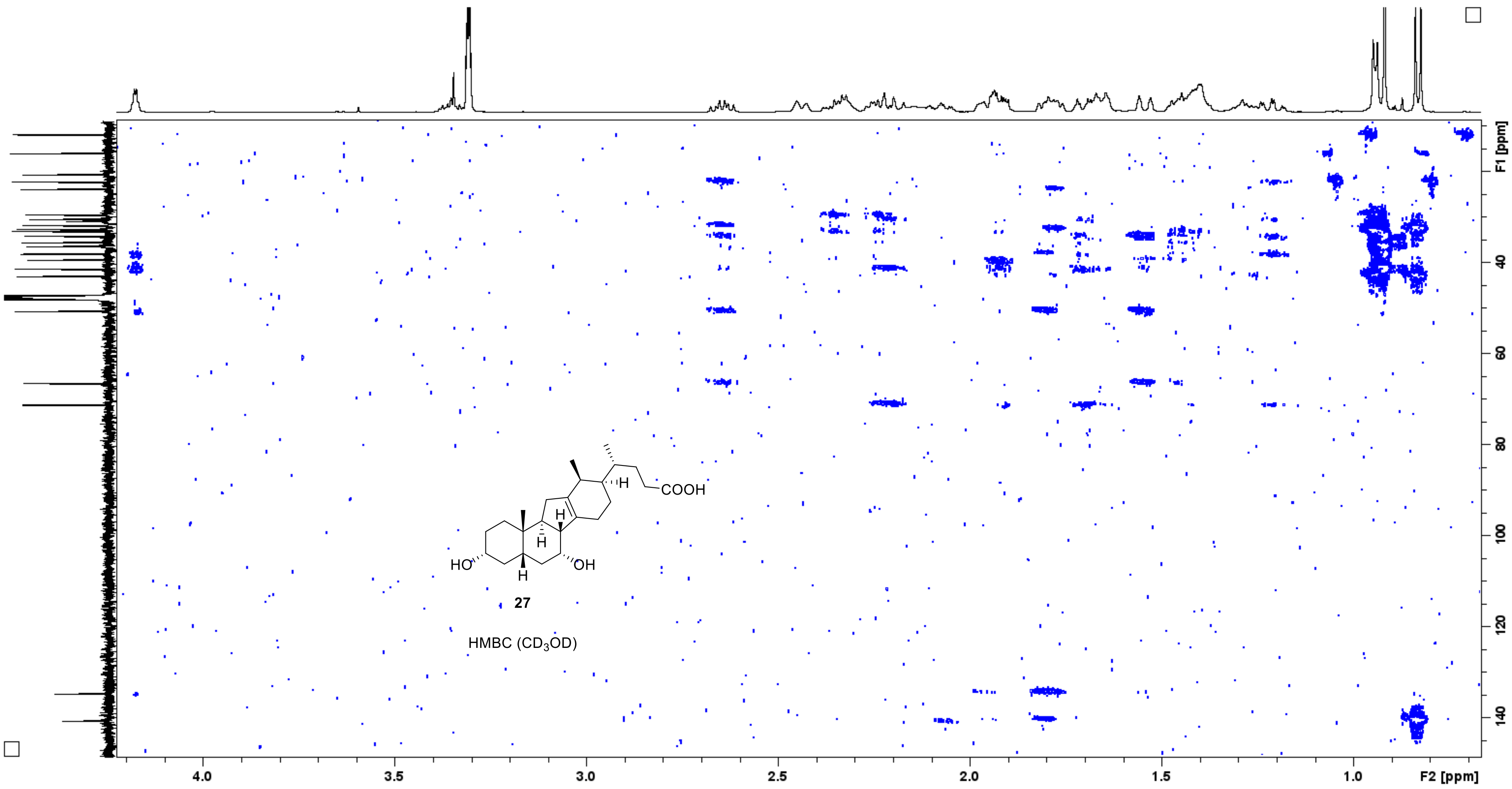
F1 [ppm]
F2 [ppm]
HO
OH
H
COOH
27
HMBC (CD_3OD)

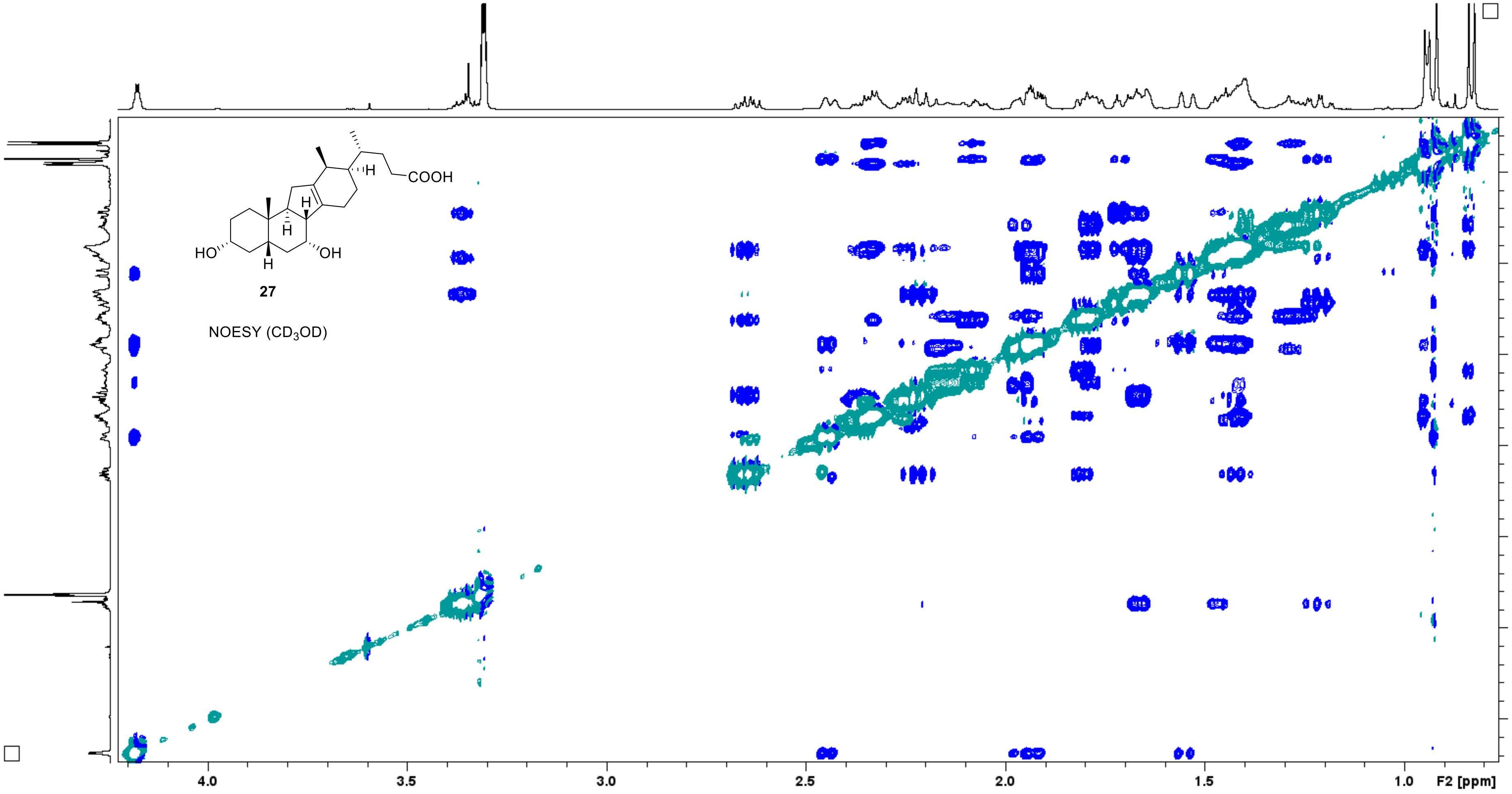
HO
OH
H
COOH
27
NOESY (CD_3OD)
F1 [ppm]
F2 [ppm]
4.0
3.5
3.0
2.5
2.0
1.5
1.0

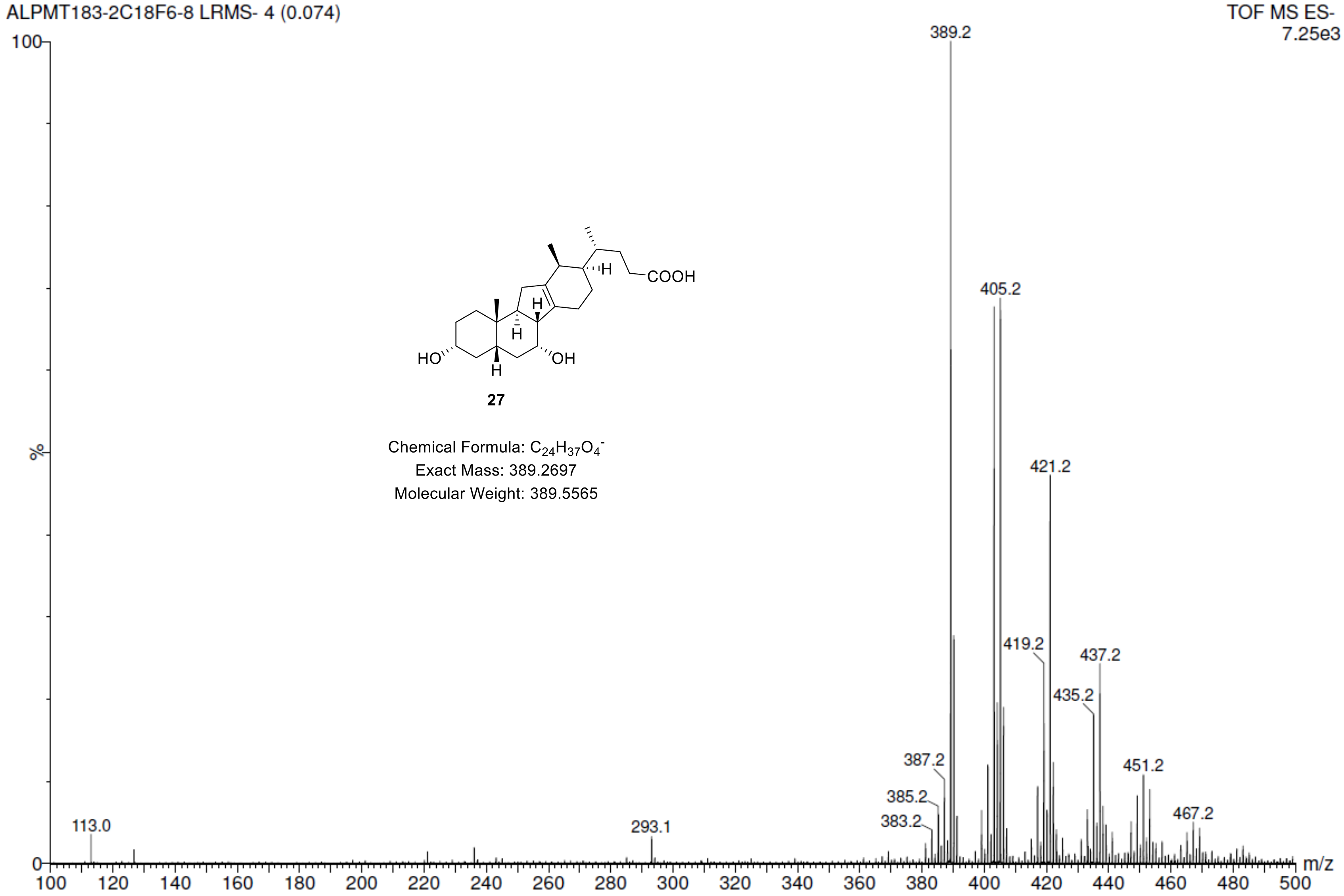

ALPMT183-2C18F6-8 LRMS- 4 (0.074)
TOF MS ES-
7.25e3
389.2
405.2
421.2
419.2
437.2
435.2
387.2
385.2
383.2
451.2
467.2
113.0
293.1
%
m/z
HO
OH
H
COOH
27
Chemical Formula: $C_{24}H_{37}O_4^-$
Exact Mass: 389.2697
Molecular Weight: 389.5565

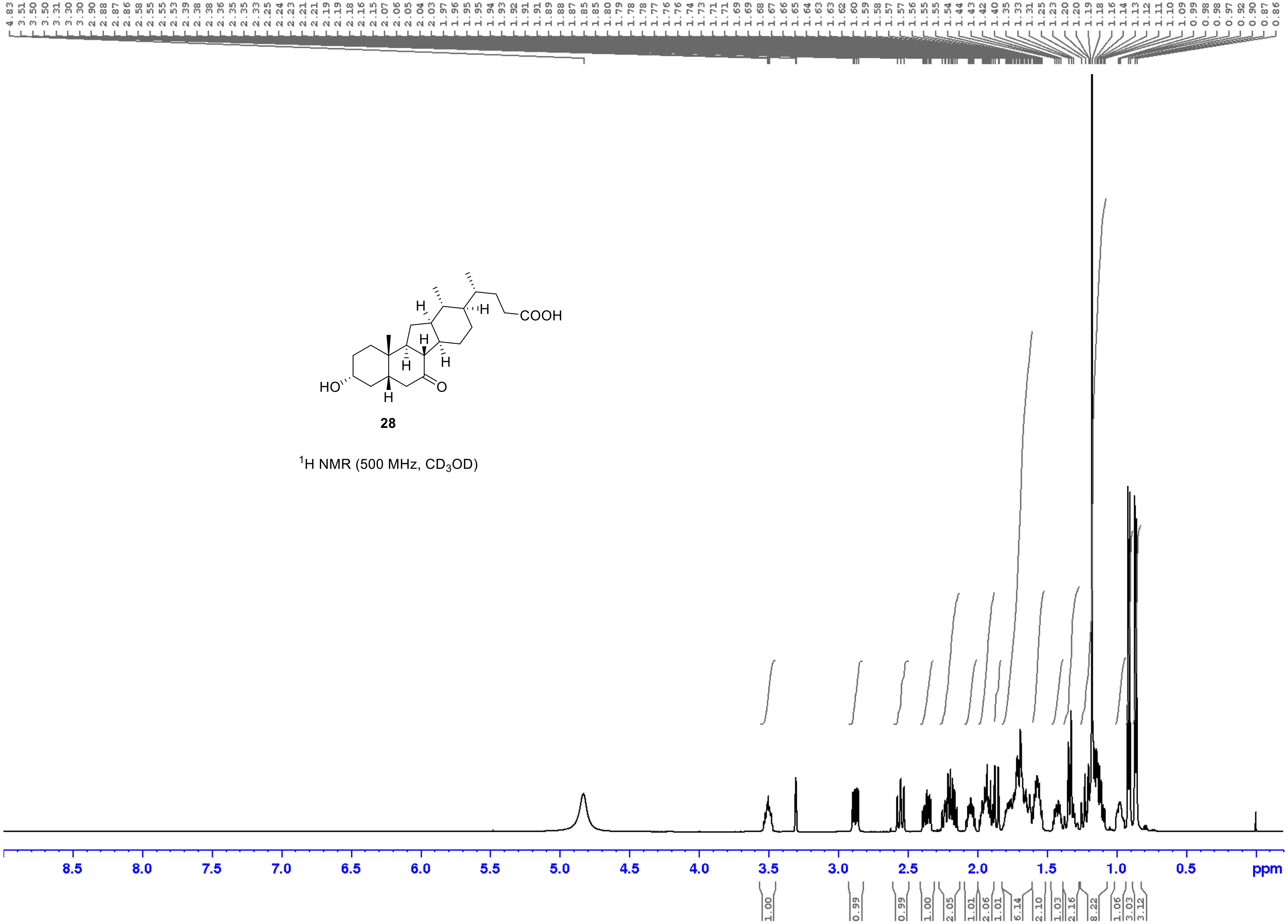

28

^{1}H NMR (500 MHz, CD_3OD)

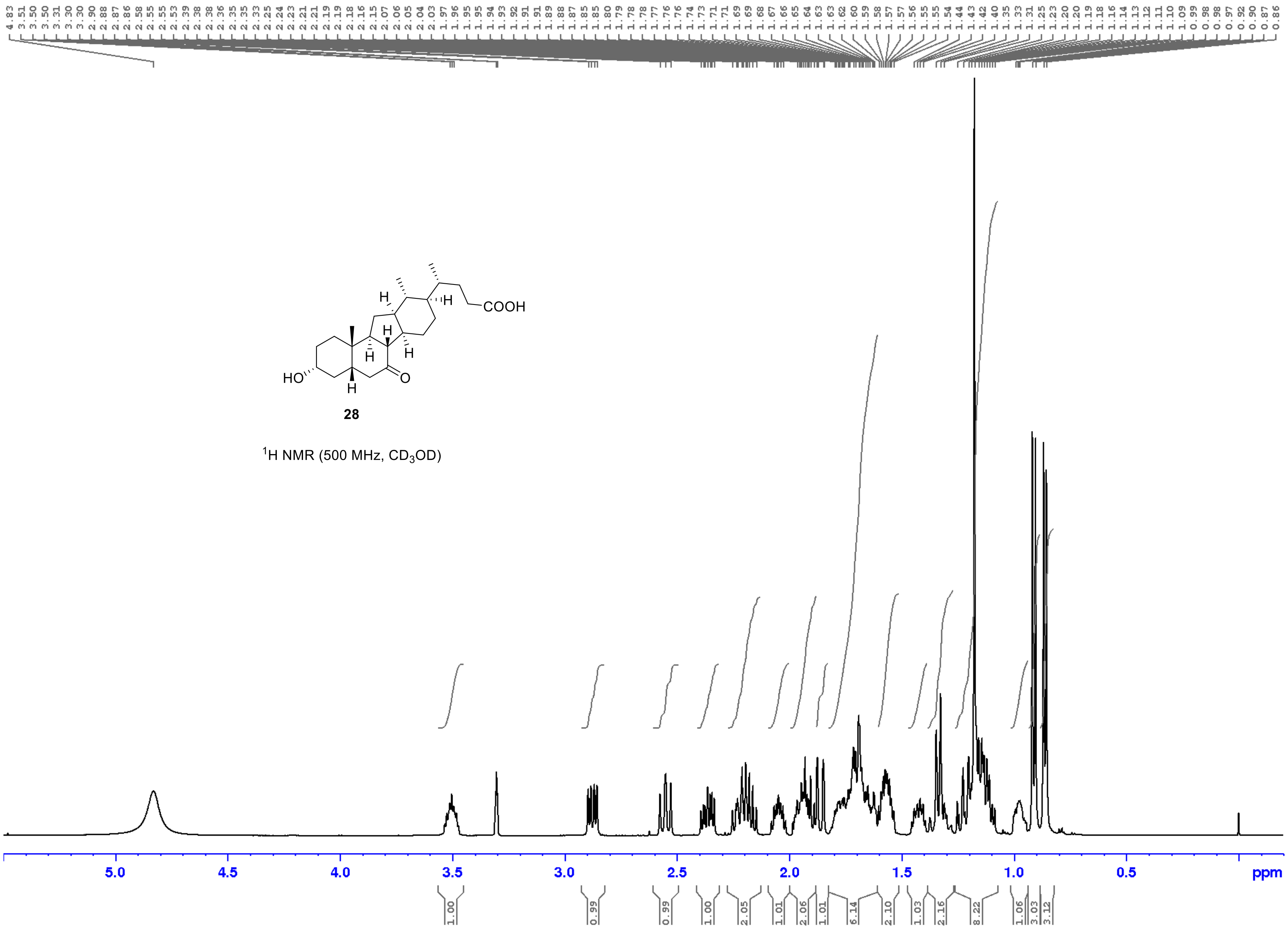

28

^{1}H NMR (500 MHz, CD_3OD)

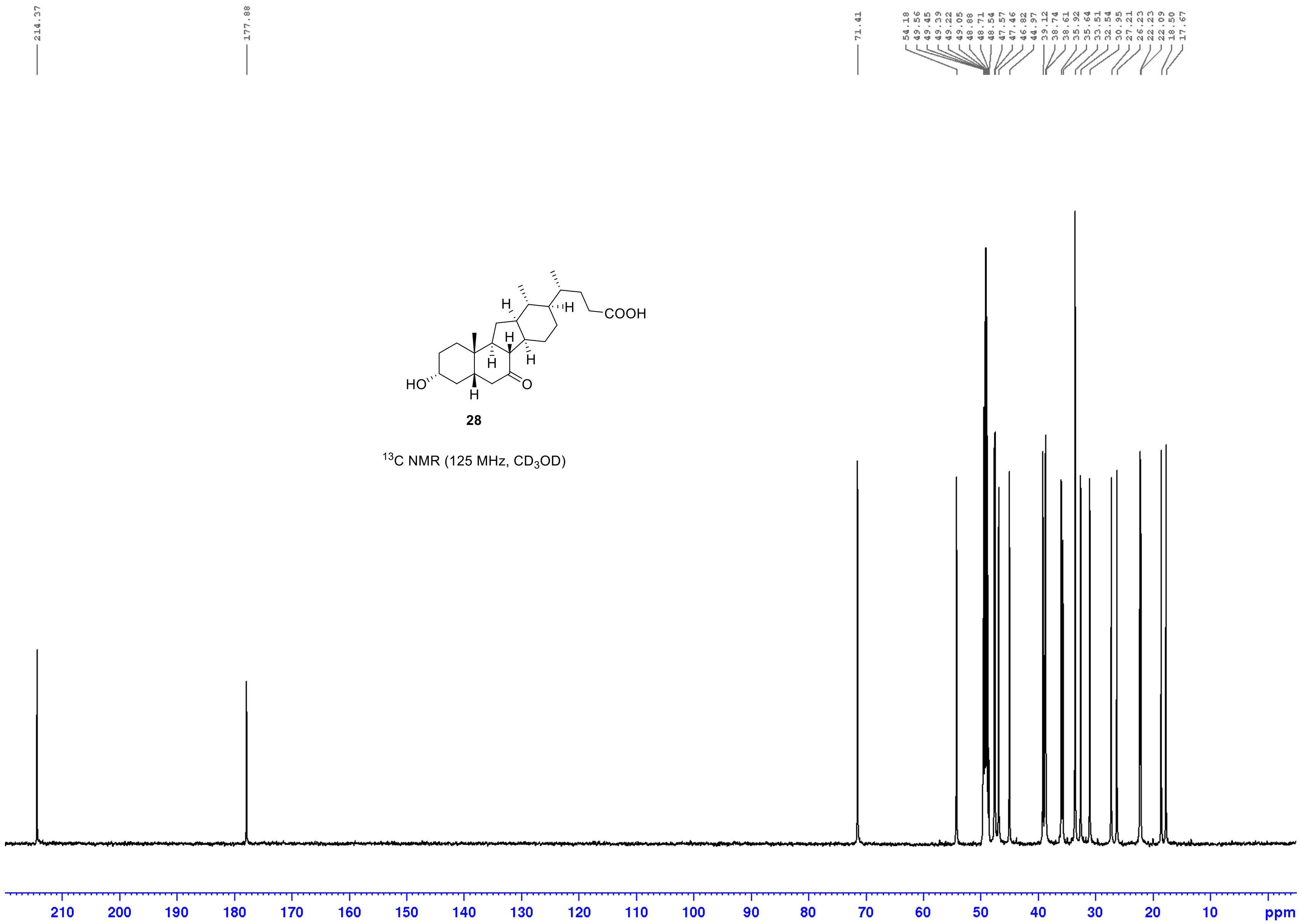

^{13}C NMR (125 MHz, CD_3OD)

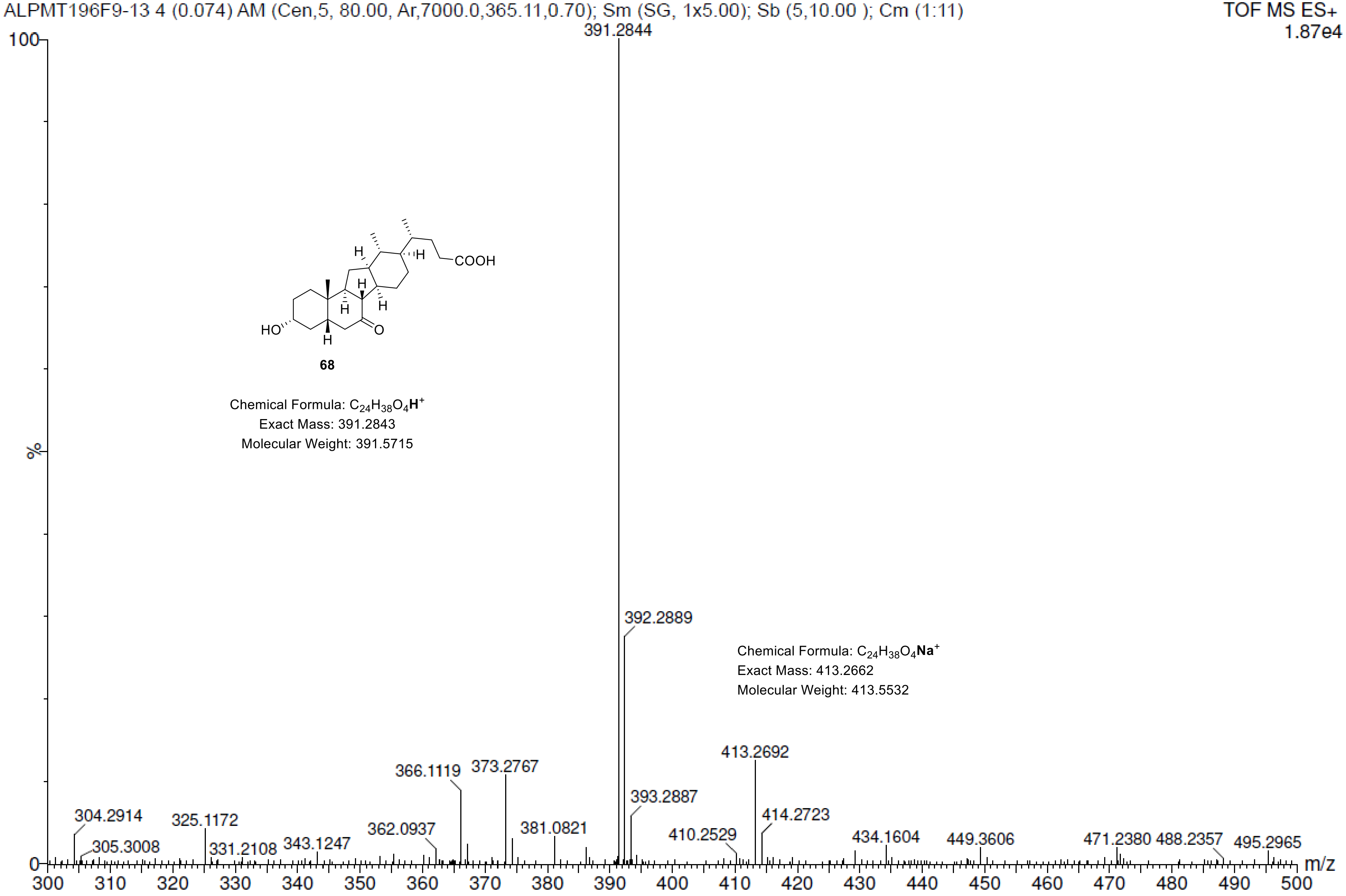
ALPMT196F9-13 4 (0.074) AM (Cen,5, 80.00, Ar,7000.0,365.11,0.70); Sm (SG, 1x5.00); Sb (5,10.00); Cm (1:11)
TOF MS ES+
1.87e4
100
%
0
391.2844
392.2889
393.2887
413.2692
414.2723
410.2529
373.2767
366.1119
362.0937
381.0821
304.2914
305.3008
325.1172
331.2108
343.1247
434.1604
449.3606
471.2380
488.2357
495.2965
300 310 320 330 340 350 360 370 380 390 400 410 420 430 440 450 460 470 480 490 500
m/z
HO
H
O
COOH
68
Chemical Formula: $C_{24}H_{38}O_4H^+$
Exact Mass: 391.2843
Molecular Weight: 391.5715
Chemical Formula: $C_{24}H_{38}O_4Na^+$
Exact Mass: 413.2662
Molecular Weight: 413.5532

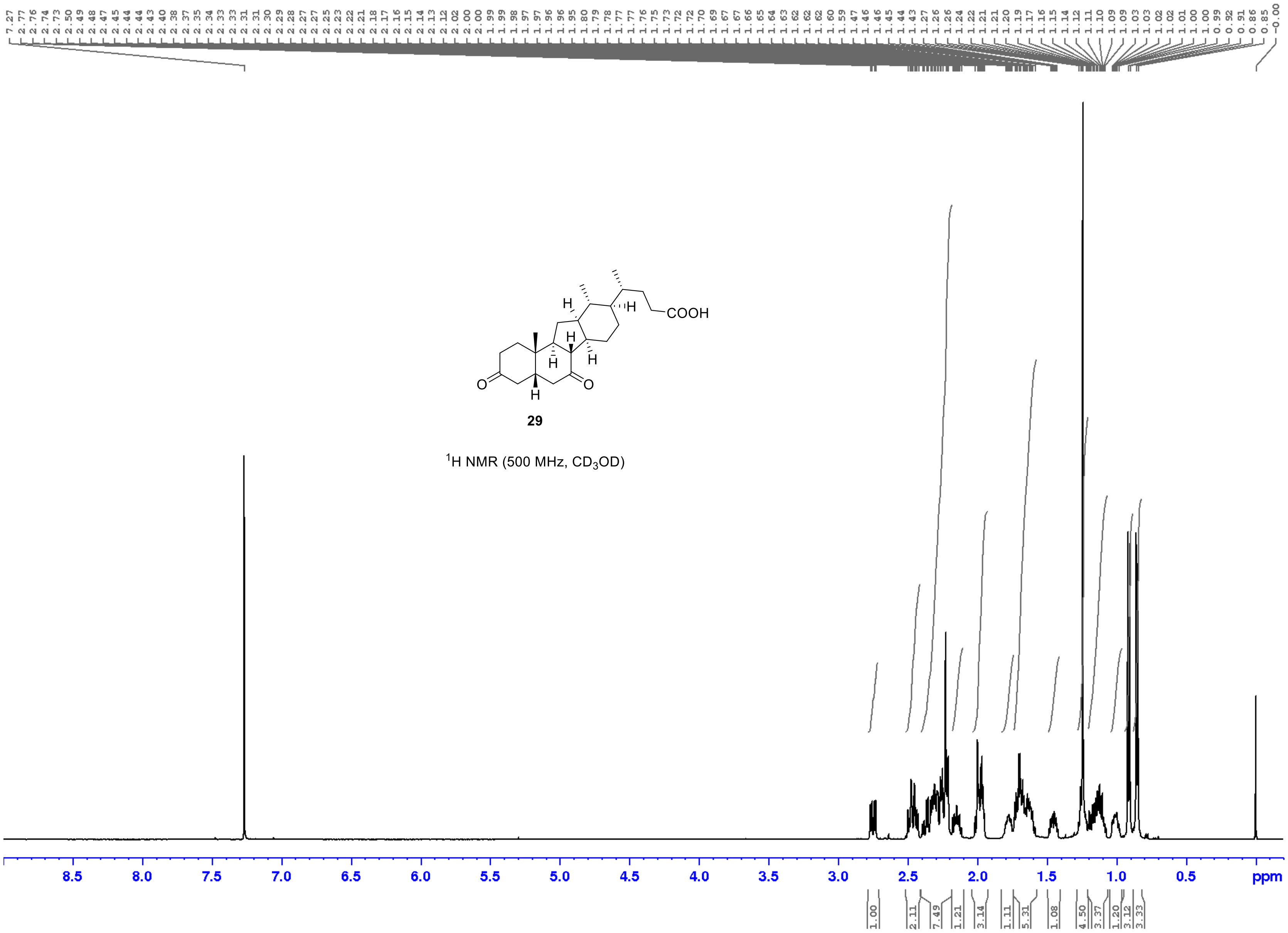

29

^{1}H NMR (500 MHz, CD_3OD)

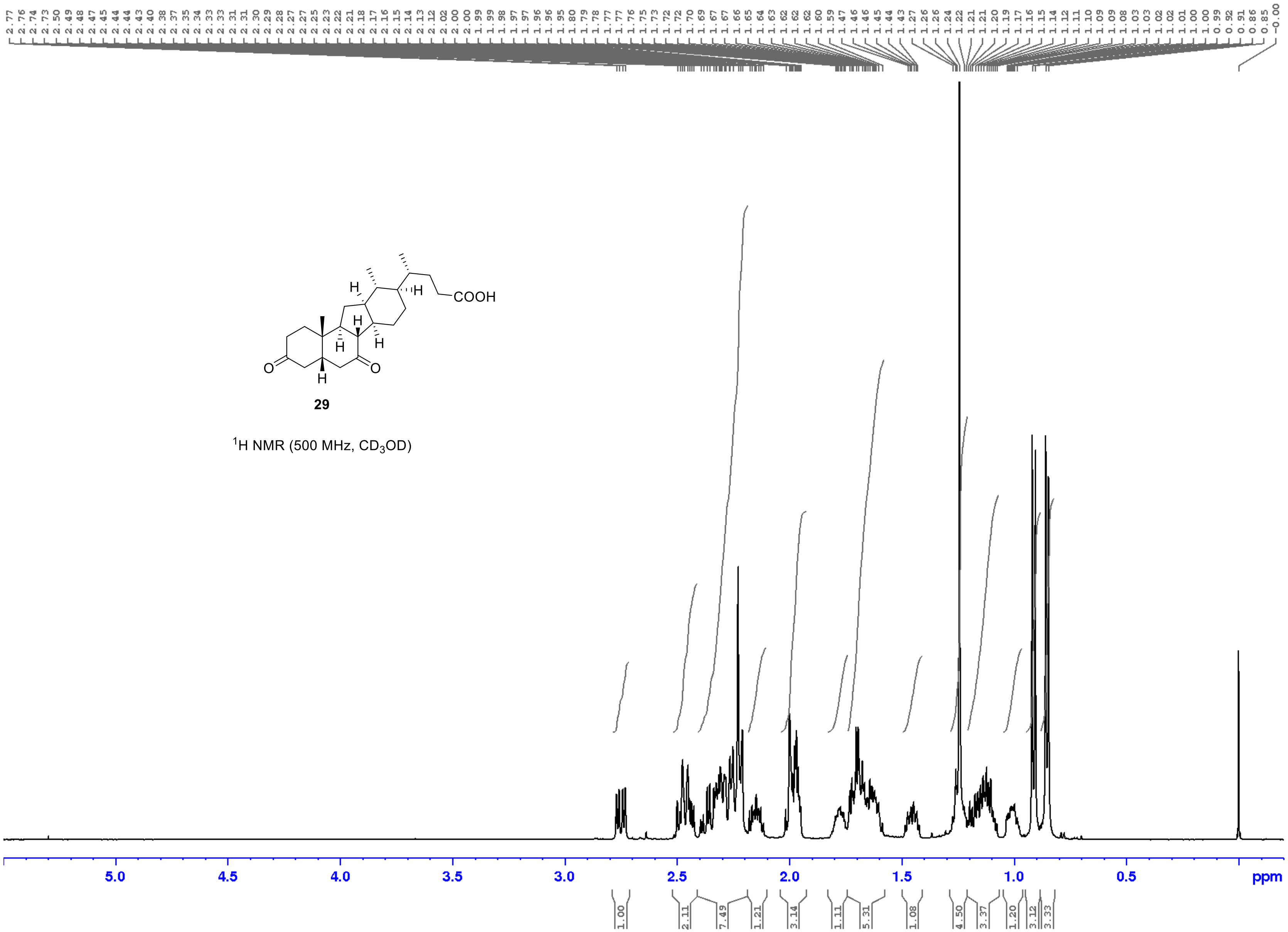

29

^{1}H NMR (500 MHz, CD_3OD)

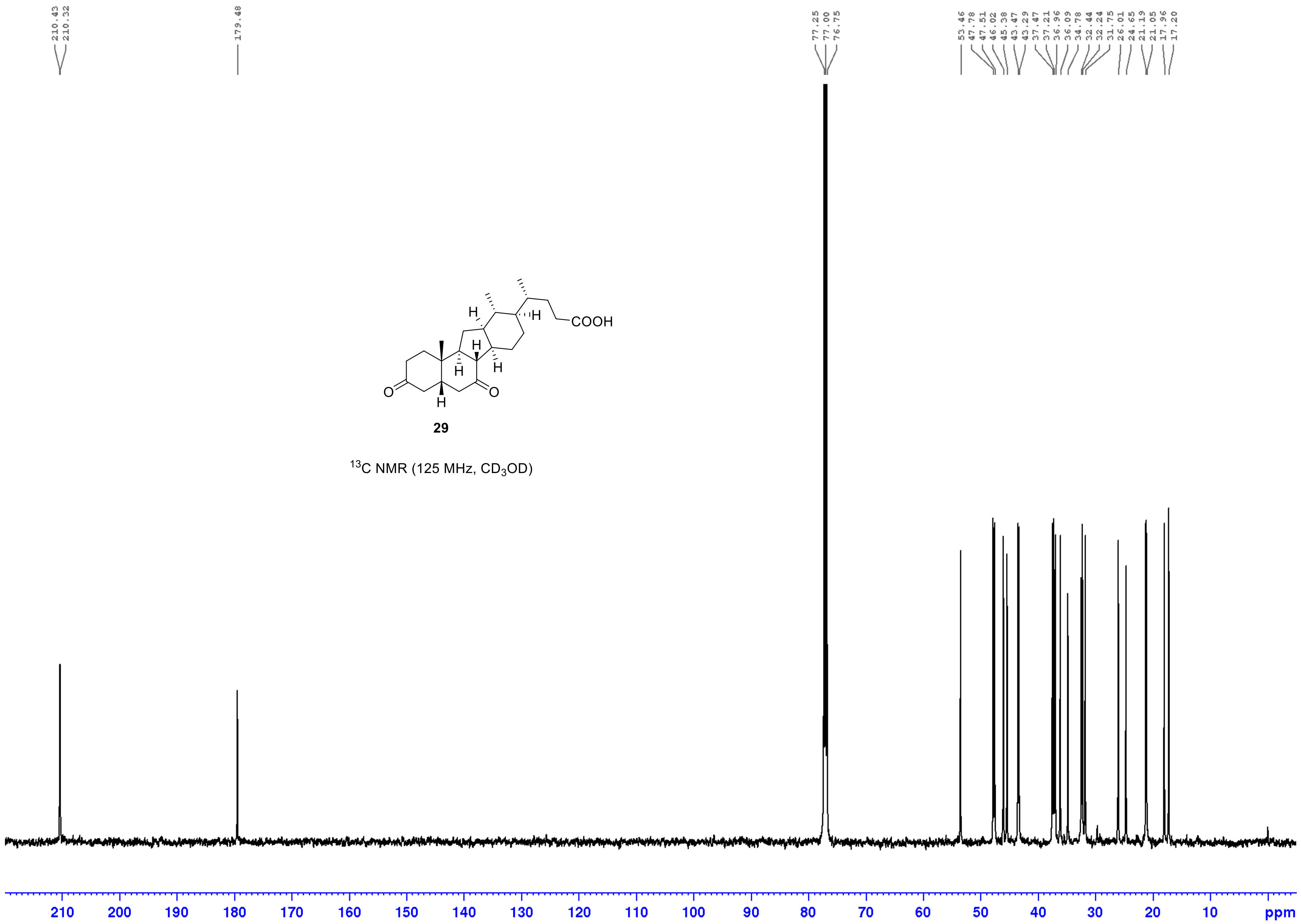

29

^{13}C NMR (125 MHz, CD_3OD)

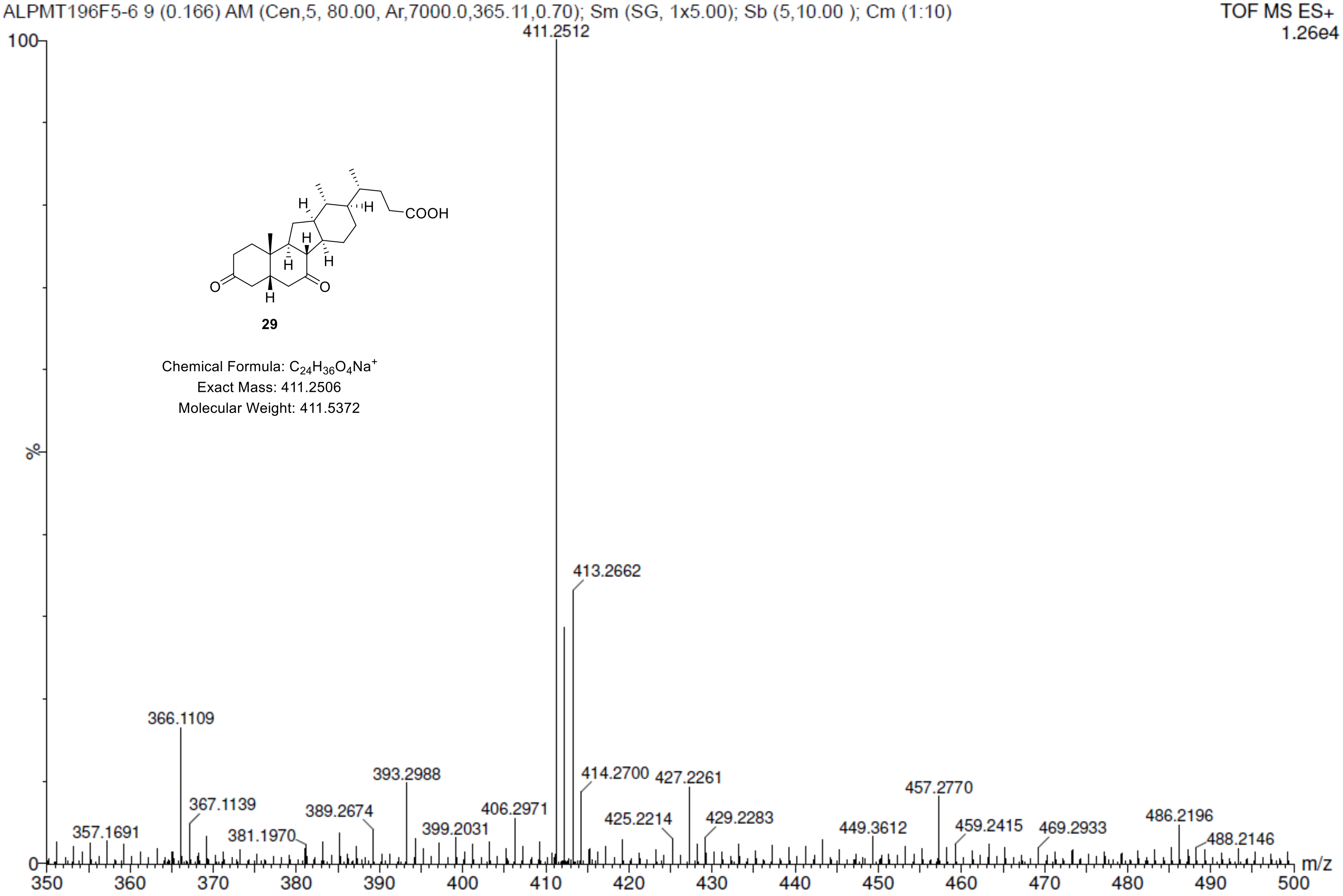
ALPMT196F5-6 9 (0.166) AM (Cen,5, 80.00, Ar,7000.0,365.11,0.70); Sm (SG, 1x5.00); Sb (5,10.00); Cm (1:10)
TOF MS ES+
1.26e4
29
Chemical Formula: $C_{24}H_{36}O_4Na^+$
Exact Mass: 411.2506
Molecular Weight: 411.5372
411.2512
413.2662
366.1109
393.2988
414.2700
427.2261
457.2770
367.1139
389.2674
406.2971
425.2214
429.2283
486.2196
357.1691
381.1970
399.2031
449.3612
459.2415
469.2933
488.2146
100
%
0
m/z

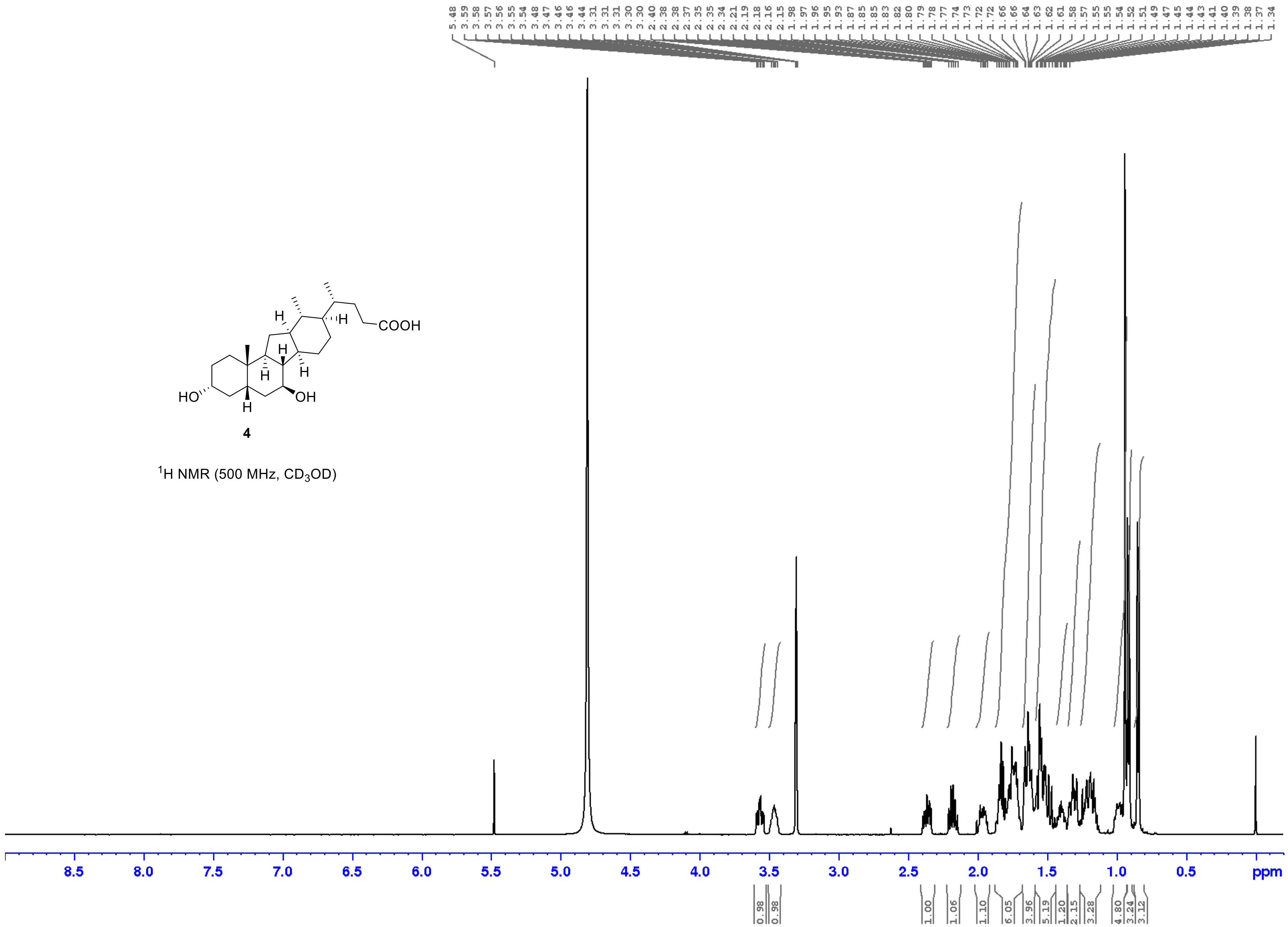

4

^1H NMR (500 MHz, CD_3OD)

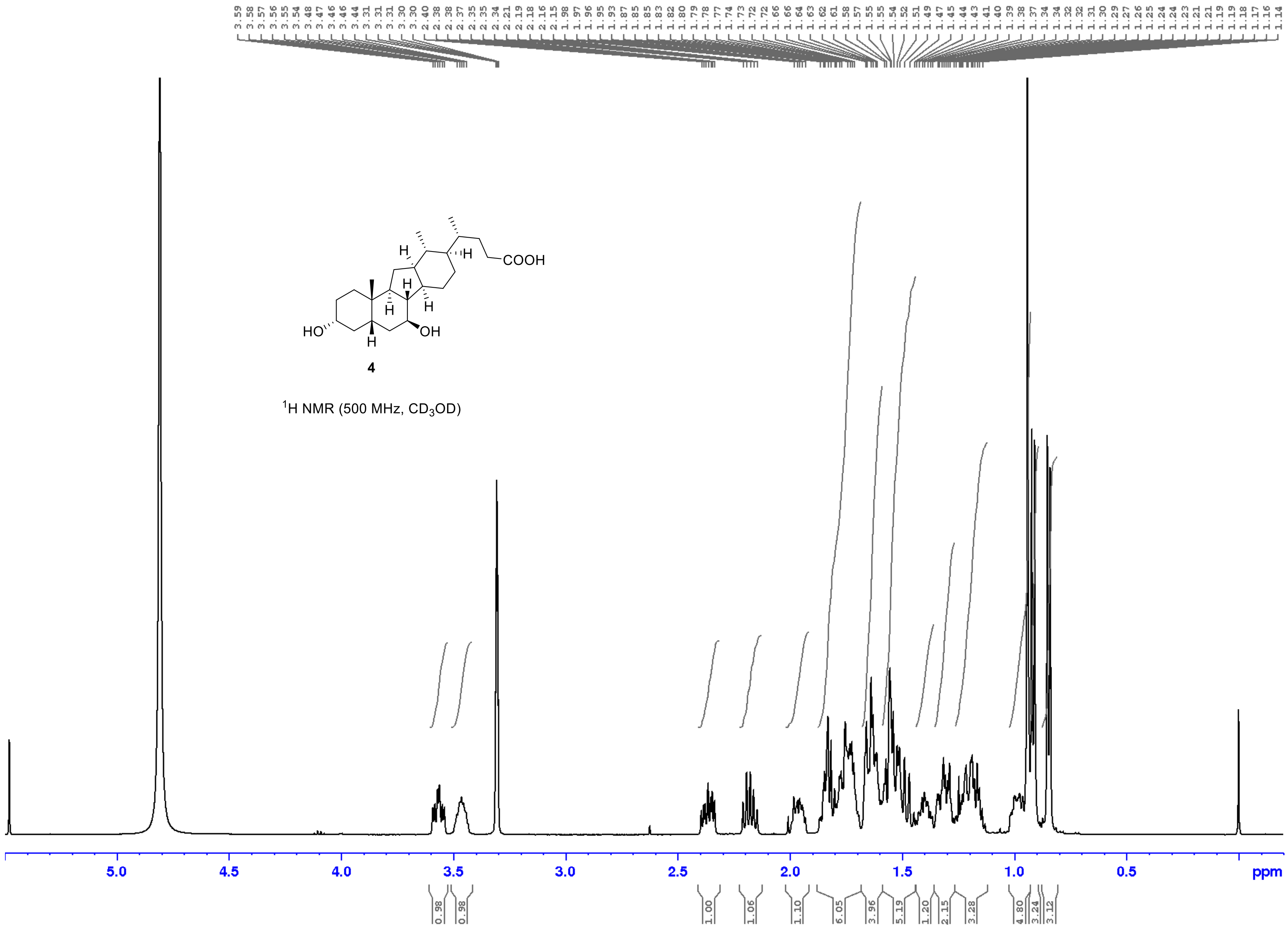
4
^{1}H NMR (500 MHz, CD$_3$OD)
ppm

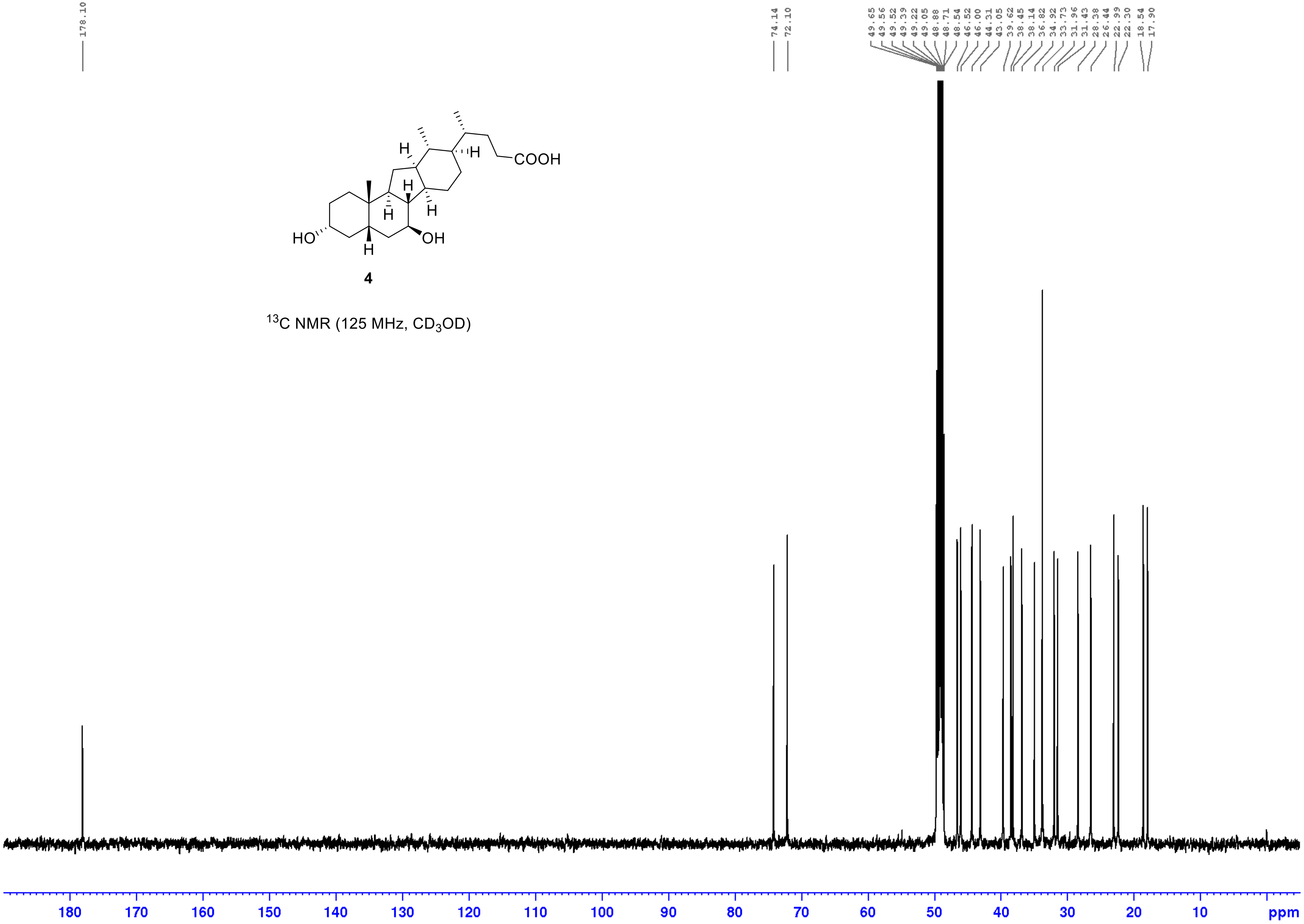
178.10
74.14
72.10
49.65
49.56
49.52
49.39
49.22
49.05
48.88
48.71
48.54
46.52
46.00
44.31
43.05
39.62
38.45
38.14
36.82
34.92
33.73
31.96
31.43
28.38
26.44
22.99
22.30
18.54
17.90
H
H
COOH
H
H
H
HO
H
OH
4
^{13}C NMR (125 MHz, CD_3OD)
180
170
160
150
140
130
120
110
100
90
80
70
60
50
40
30
20
10
ppm

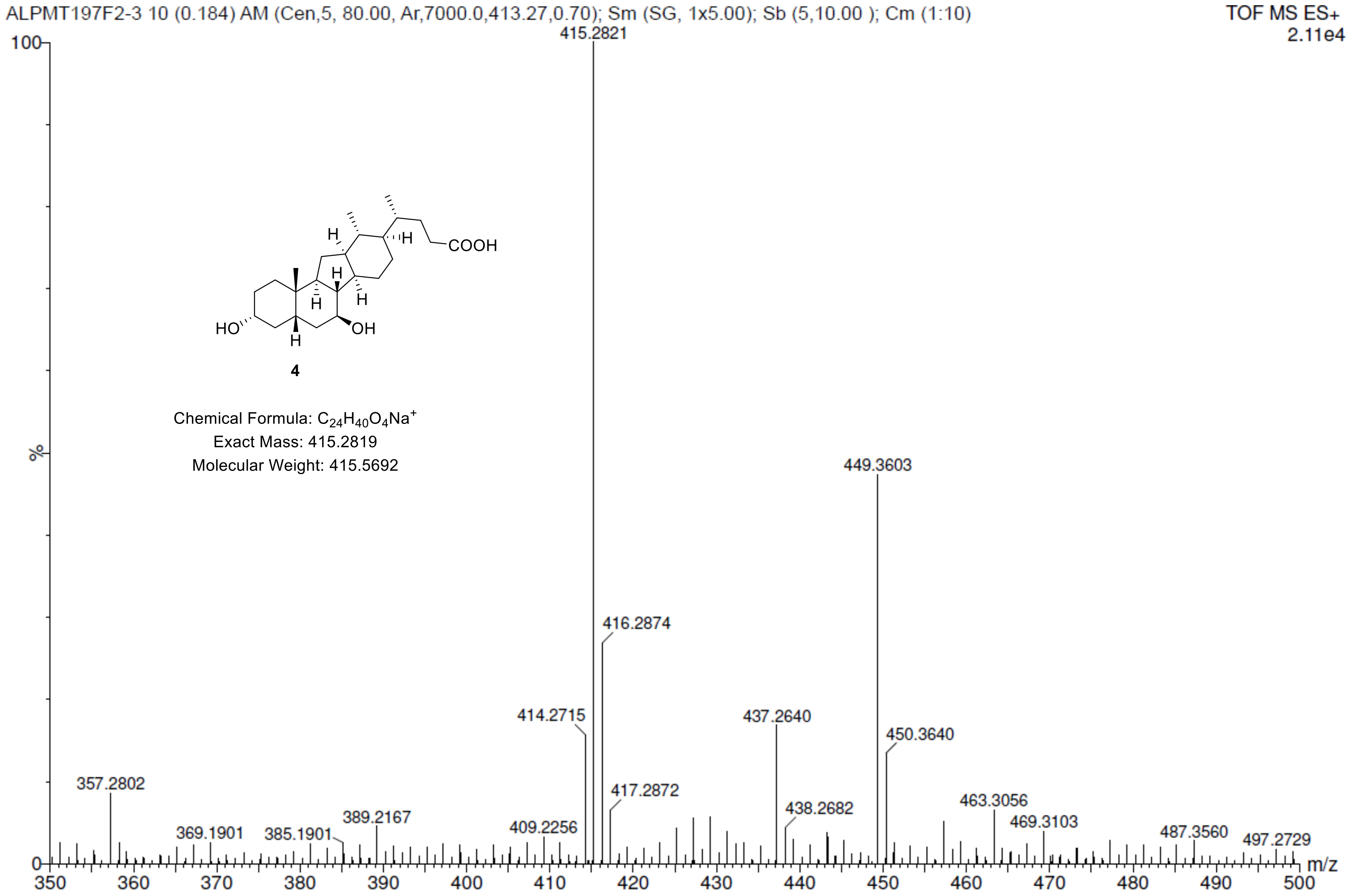
ALPMT197F2-3 10 (0.184) AM (Cen,5, 80.00, Ar,7000.0,413.27,0.70); Sm (SG, 1x5.00); Sb (5,10.00); Cm (1:10)
TOF MS ES+
2.11e4
COOH
HO
OH
H
4
Chemical Formula: $C_{24}H_{40}O_4Na^+$
Exact Mass: 415.2819
Molecular Weight: 415.5692
415.2821
449.3603
416.2874
414.2715
437.2640
450.3640
417.2872
357.2802
438.2682
463.3056
389.2167
469.3103
409.2256
369.1901
385.1901
487.3560
497.2729
100
%
0
350
360
370
380
390
400
410
420
430
440
450
460
470
480
490
500
m/z

4. HPLC chromatograms

4.1. HPLC analysis of 3,7-diacetate-12-oxo methyl ester 17 reductions:

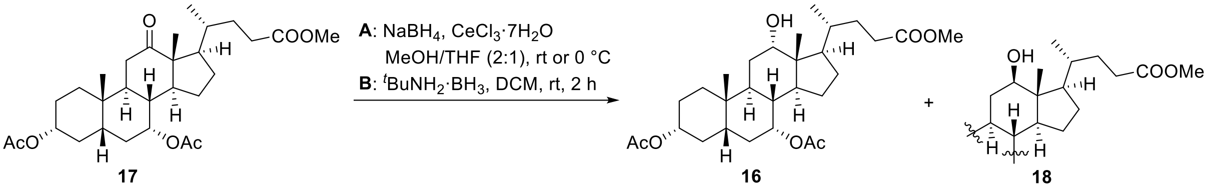

Method conditions:

Column:	Waters Spherisorb ODS2 5 µm 250×4.6 mm		
Guard:	Phenomenex Security Guard C18 RP 4×3 mm		
Mobile Phase:	30:70:0.05 Water/Methanol/Trifluoroacetic acid		
Gradient:	Isocratic;	Flow Rate:	1 mL/min
Sample Solvent:	Methanol;	Detection:	*Refractive index*
Column Temp:	20 °C;	Injection volume:	30 µL

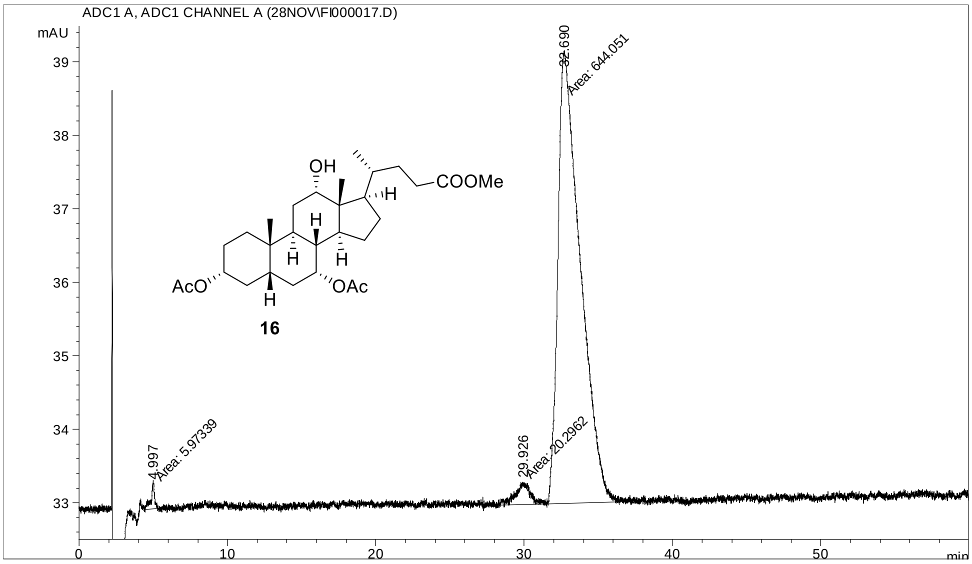

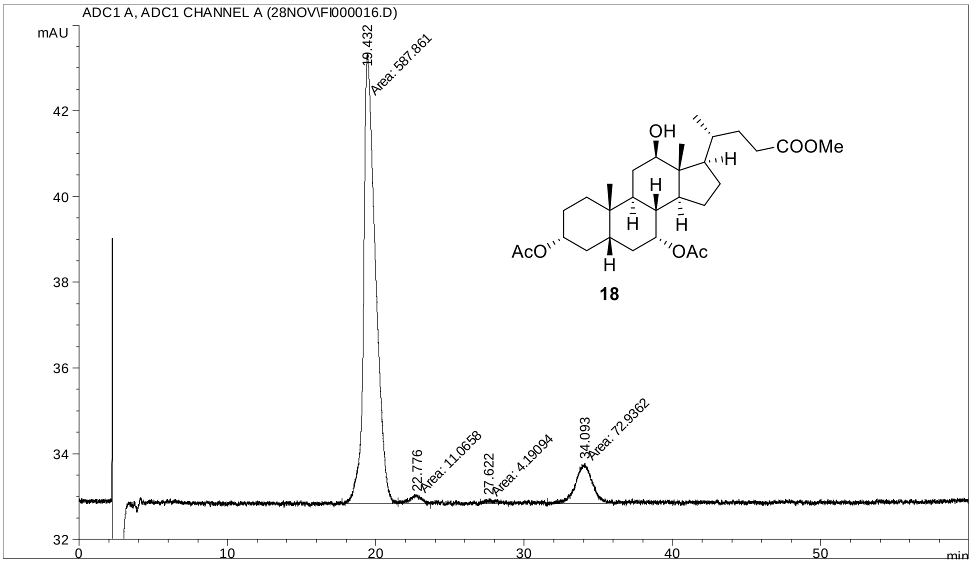

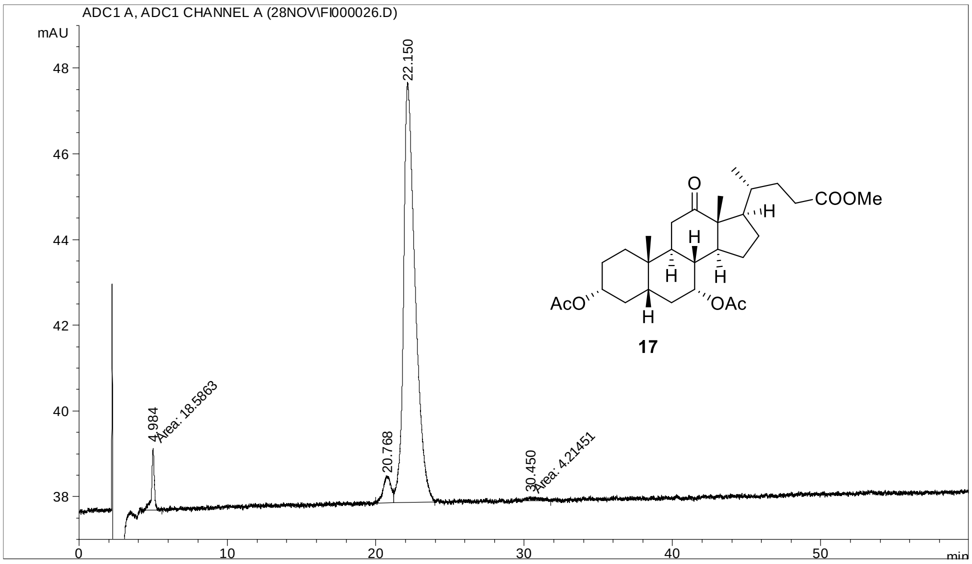
ADC1 A, ADC1 CHANNEL A (28NOV\FI000026.D)
mAU
48
46
44
42
40
38
0
10
20
30
40
50
min
4.984
Area: 18.5863
20.768
22.150
30.450
Area: 4.21451
O
H
COOMe
AcO
OAc
17

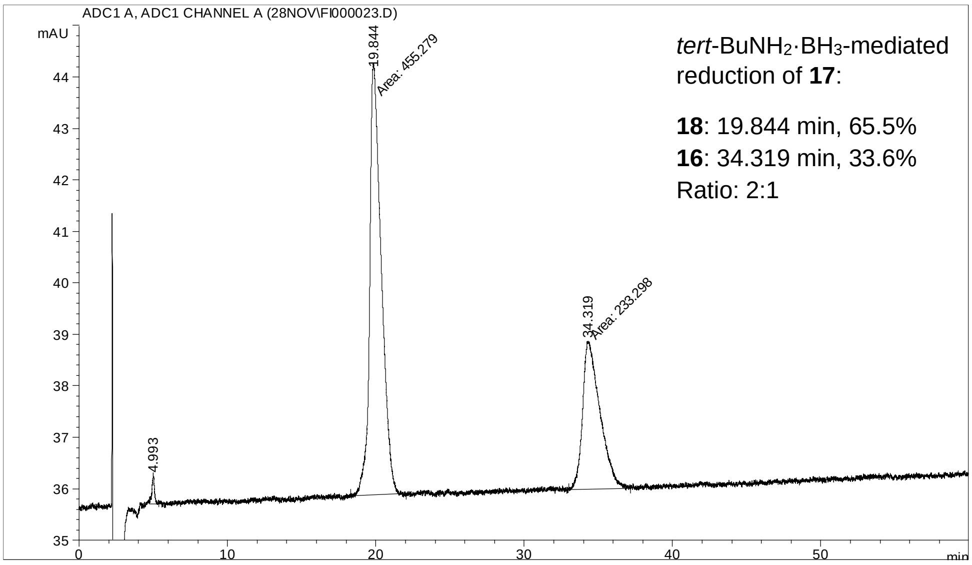
ADC1 A, ADC1 CHANNEL A (28NOV\FI000023.D)
mAU
44
43
42
41
40
39
38
37
36
35
0
10
20
30
40
50
min
4.993
19.844
Area: 455.279
34.319
Area: 233.298
tert-BuNH2·BH3-mediated reduction of **17**:
18: 19.844 min, 65.5%
16: 34.319 min, 33.6%
Ratio: 2:1

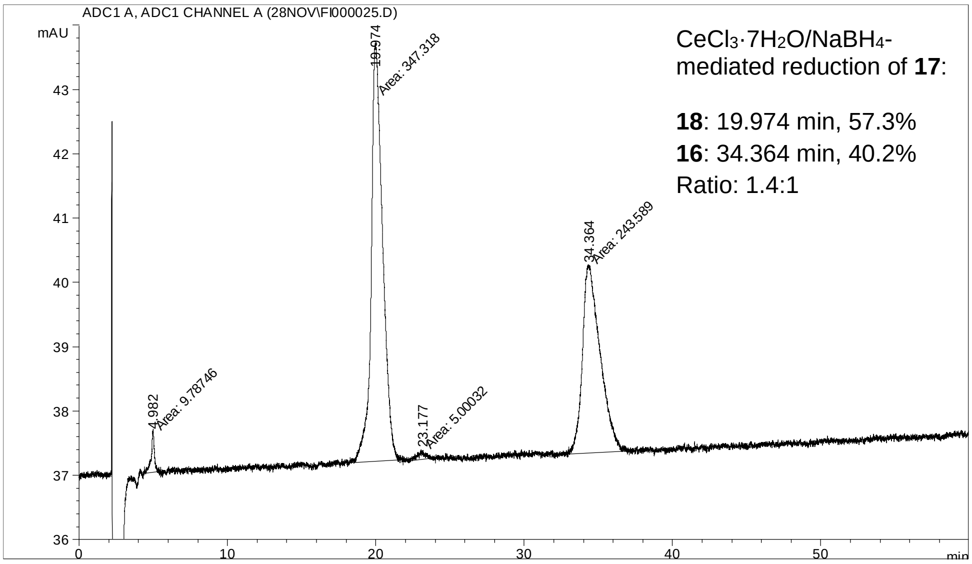
ADC1 A, ADC1 CHANNEL A (28NOV\FI000025.D)
mAU
43
42
41
40
39
38
37
36
0
10
20
30
40
50
min
4.982
Area: 9.78746
19.974
Area: 347.318
23.177
Area: 5.00032
34.364
Area: 243.589
CeCl3·7H2O/NaBH4-mediated reduction of **17**:
18: 19.974 min, 57.3%
16: 34.364 min, 40.2%
Ratio: 1.4:1

4.2. HPLC analysis of Meerwein-Ponndorf-Verley (MPV) reduction of 3,7-diacetate-12-oxo methyl ester 17 reductions:

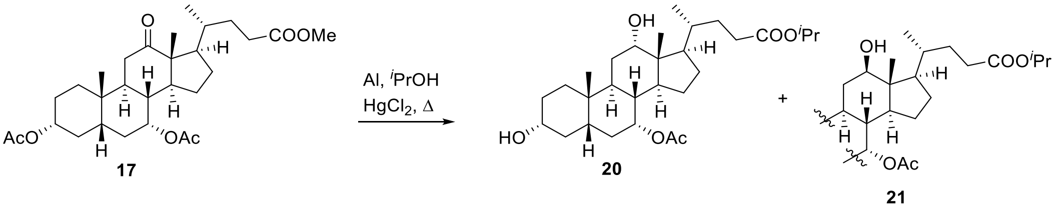

Method conditions:

Column:	Waters Spherisorb ODS2 5 µm 250×4.6 mm		
Guard:	Phenomenex Security Guard C18 RP 4×3 mm		
Mobile Phase:	30:70:0.05 Water/Methanol/Trifluoroacetic acid		
Gradient:	Isocratic;	Flow Rate:	1 mL/min
Sample Solvent:	Methanol;	Detection:	*Refractive index*
Column Temp:	20 °C;	Injection volume:	30 µL

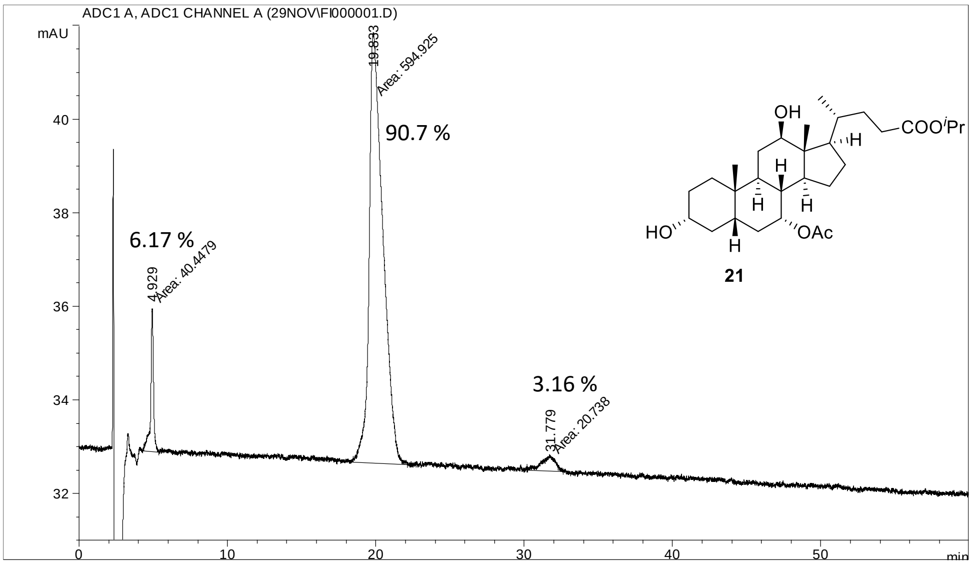

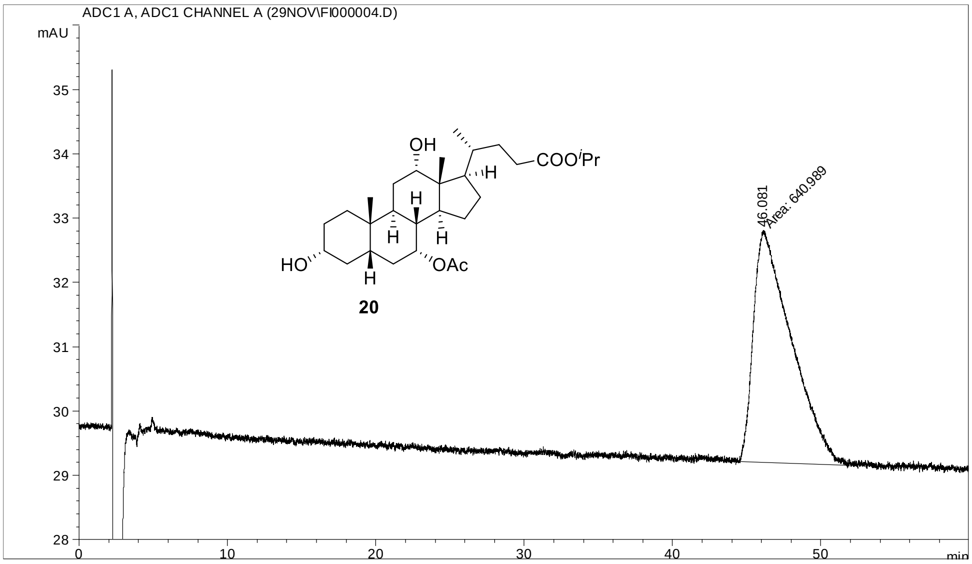

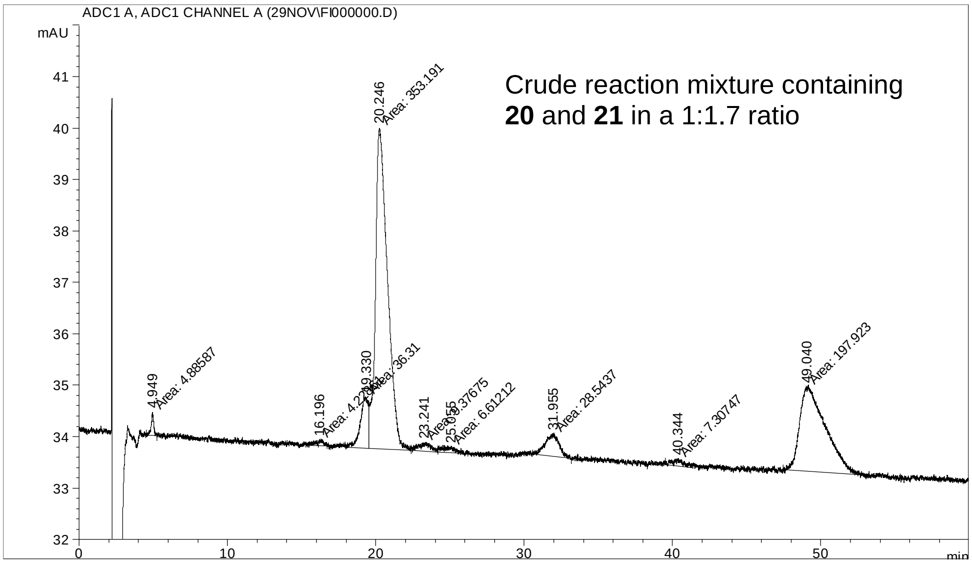

Peak #	RetTime [min]	Type	Width [min]	Area [mAU*s]	Height [mAU]	Area %
1	4.949	MM	0.1838	4.88587	4.42979e-1	0.7536
2	16.196	MM	0.6348	4.22861	1.11023e-1	0.6522
3	19.330	MF	0.6027	36.31005	1.00410	5.6001
4	20.246	FM	0.9425	353.19110	6.24580	54.4730
5	23.241	MF	0.9257	9.37675	1.68830e-1	1.4462
6	25.055	FM	1.0066	6.61212	1.09479e-1	1.0198
7	31.955	MM	1.0994	28.54368	4.32699e-1	4.4023
8	40.344	MM	0.9193	7.30747	1.32488e-1	1.1270
9	49.040	MM	2.0256	197.92293	1.62849	30.5258

Totals : 648.37860 10.27589

4.3. HPLC analysis of crude rearrangement mixture 22/23:

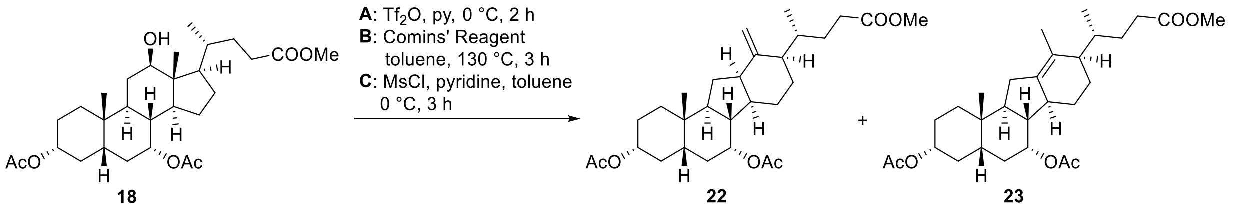

Method conditions:

Column: Phenomenex Kinetex, 2.6 µM C-18, pore size = 100 Å, Dimensions = 50 × 30 mm
Mobile Phase A: Water (+ 0.1% Formic acid)
Mobile Phase B: Methanol
Method (% B): T0=72, T12=72, T13=100, T14=100, T16=72, T18=72
Flow Rate: 0.5 mL/min
Sample Solvent: Methanol; Detection: CAD
Column Temp: 40 °C; Injection volume: 1 µL

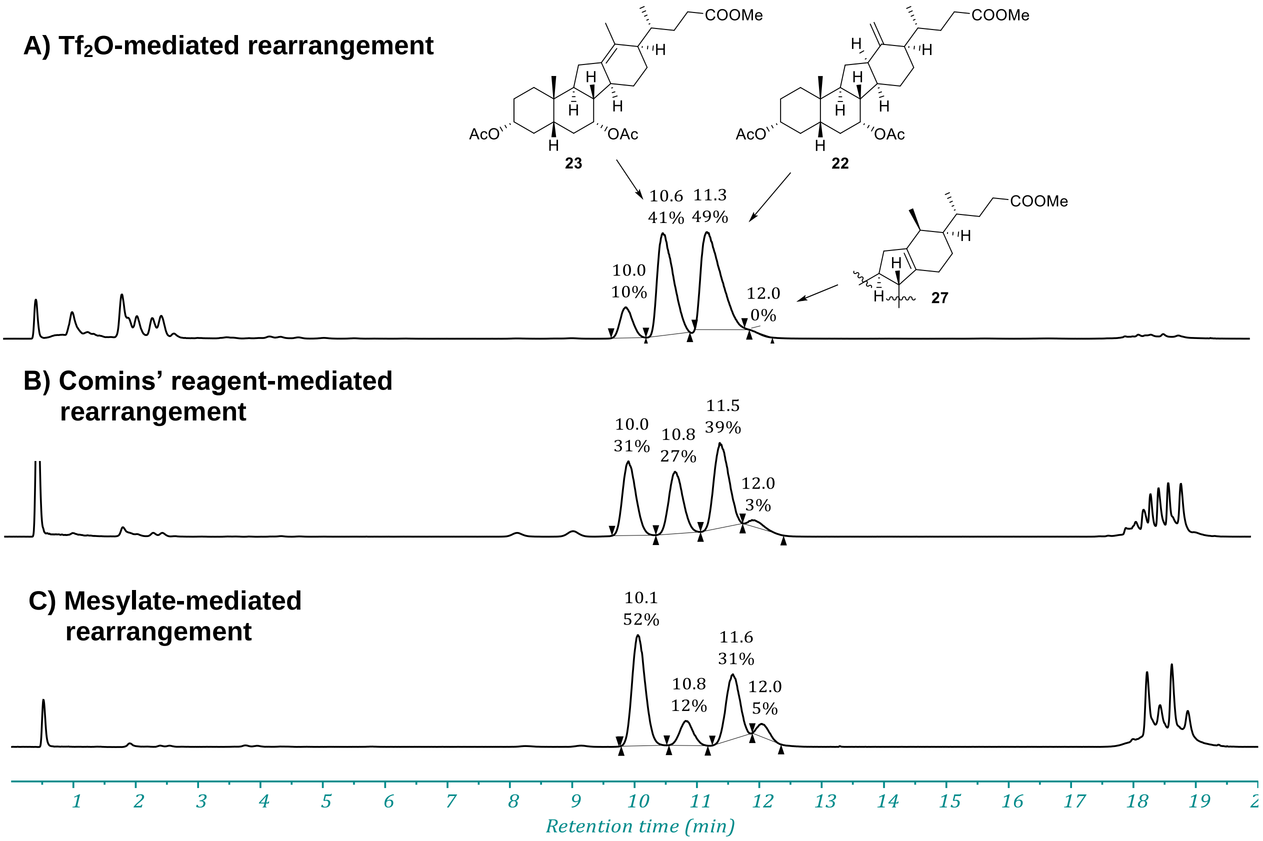

4.4. Individual HPLC traces of compounds 4, 6, 28 and 29:

Method conditions:

Column: Phenomenex Luna C18(2) 5 µm 250×4.6 mm
Guard: Phenomenex Security Guard C18 RP 4×3 mm
Mobile Phase: 30:70:0.05 Water/Methanol/Trifluoroacetic acid
Gradient: Isocratic
Flow Rate: 1 mL/min
Sample Solvent: Methanol
Detection: *Refractive index*
Column Temp: 20 °C
Injection volume: 30 µL

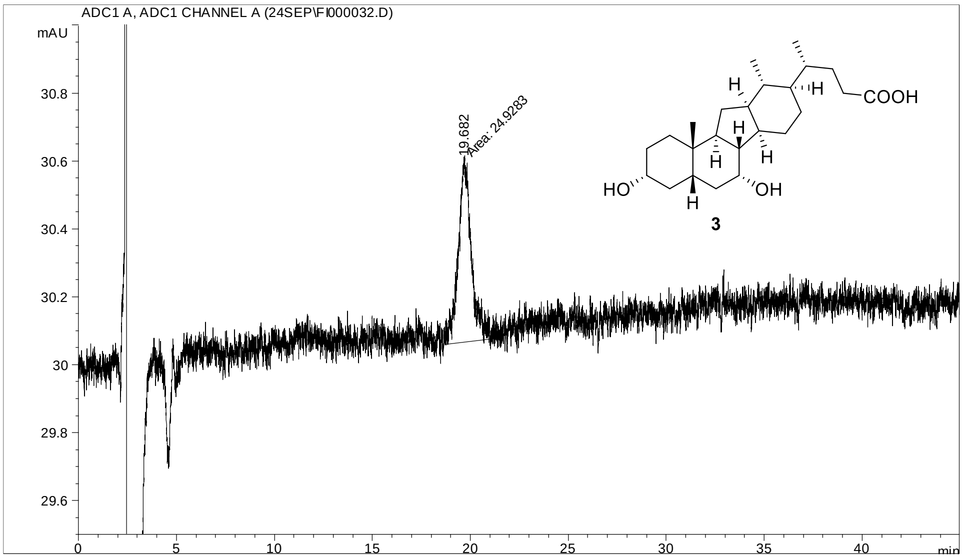

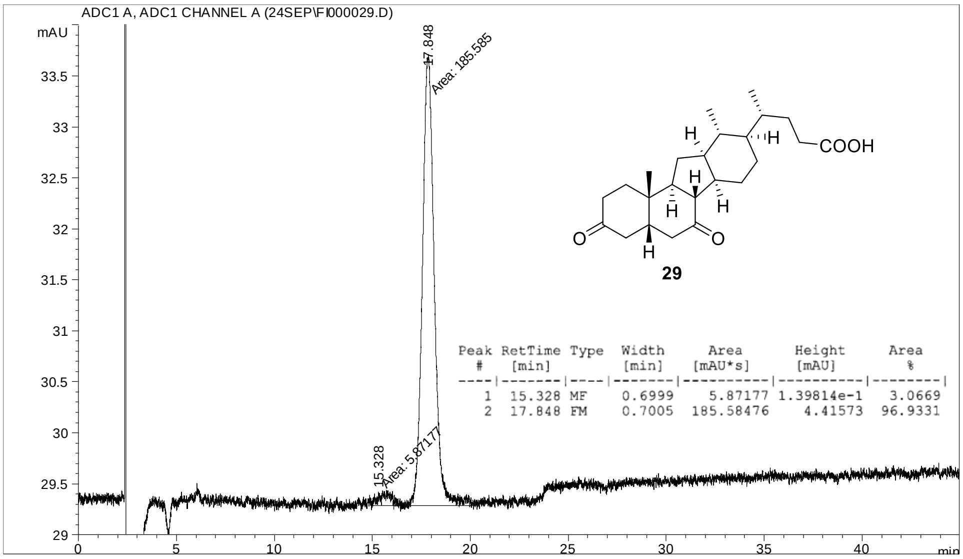

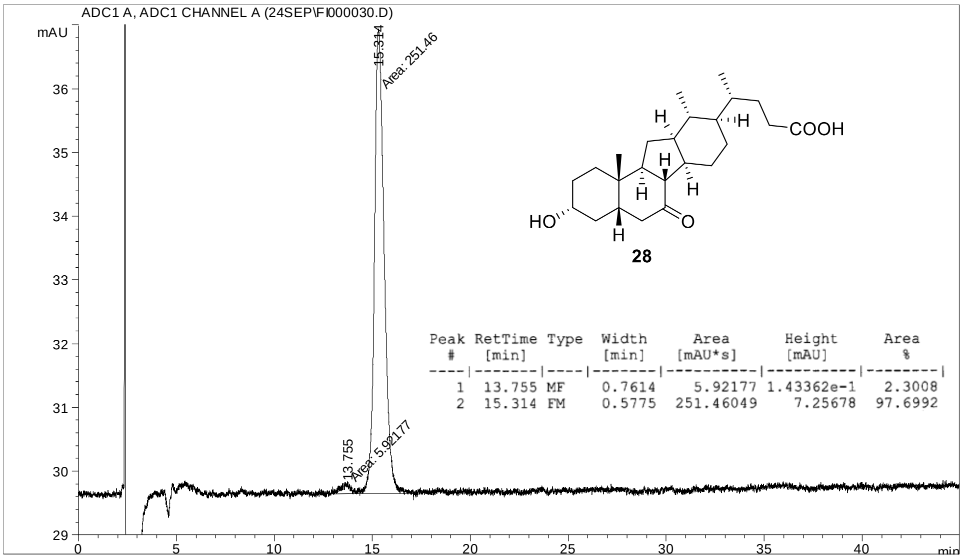

Peak #	RetTime [min]	Type	Width [min]	Area [mAU*s]	Height [mAU]	Area %
1	13.755	MF	0.7614	5.92177	1.43362e-1	2.3008
2	15.314	FM	0.5775	251.46049	7.25678	97.6992

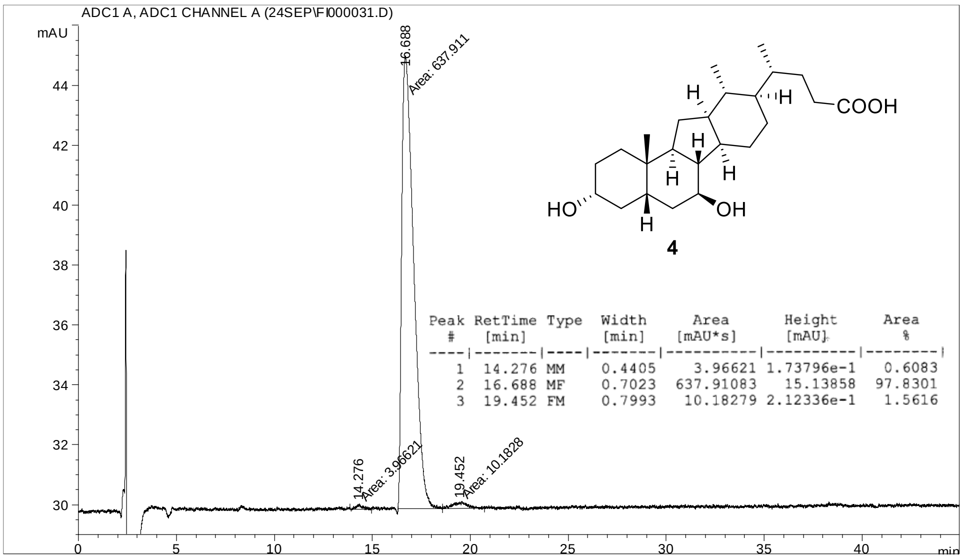

Peak #	RetTime [min]	Type	Width [min]	Area [mAU*s]	Height [mAU]	Area %
1	14.276	MM	0.4405	3.96621	1.73796e-1	0.6083
2	16.688	MF	0.7023	637.91083	15.13858	97.8301
3	19.452	FM	0.7993	10.18279	2.12336e-1	1.5616

Individual HPLC trace of compounds 6:

Method conditions:

Column: Phenomenex Luna C18(2) 5 µm 250×4.6 mm
Guard: Phenomenex Security Guard C18 RP 4×3 mm
Mobile Phase: 40:60:0.05 Water/Methanol/Trifluoroacetic acid
Gradient: Isocratic
Flow Rate: 1 mL/min
Sample Solvent: Methanol
Detection: *Refractive index*
Column Temp: 20 °C
Injection volume: 20 µL

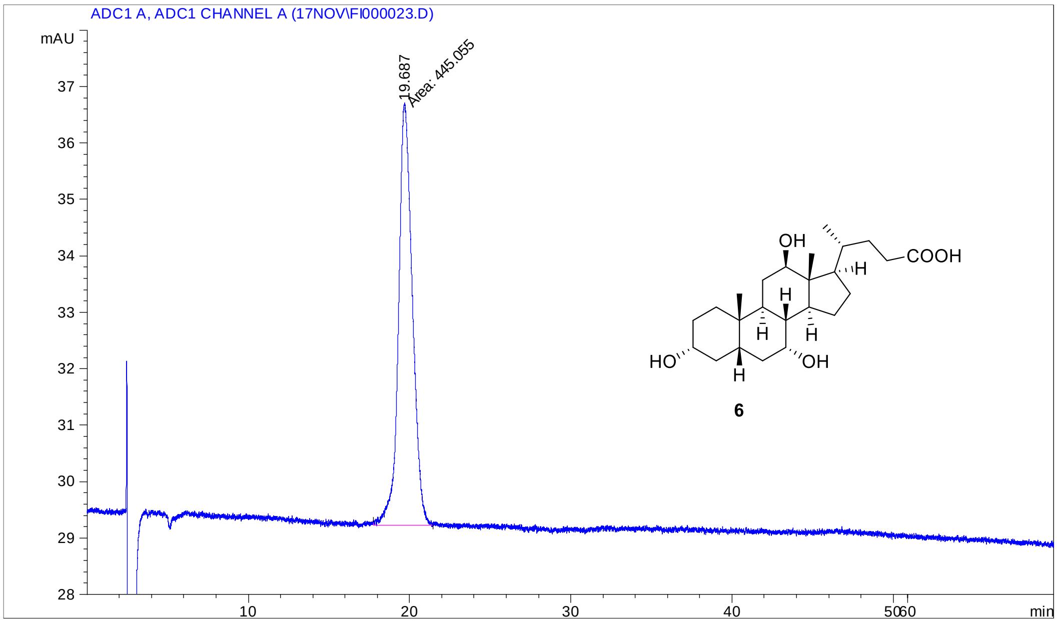

Individual HPLC traces of compounds 25 and 26:

Method conditions:

Column: Phenomenex Lux Amylose-2 5 µm 250×4.6 mm
Guard: Phenomenex Security Guard Amylose-2 4×3 mm
Mobile Phase: 40:60 Water/Acetonitrile
Gradient: Isocratic
Flow Rate: 1 mL/min
Sample Solvent: Acetonitrile
Detection: *Refractive index*
Column Temp: 20 °C
Injection volume: 10 µL

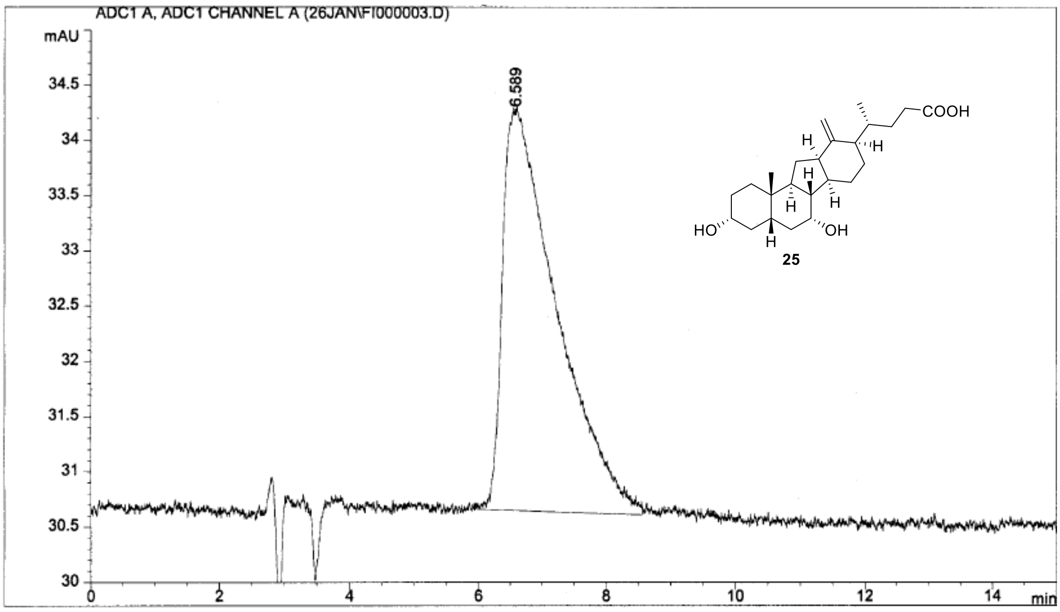

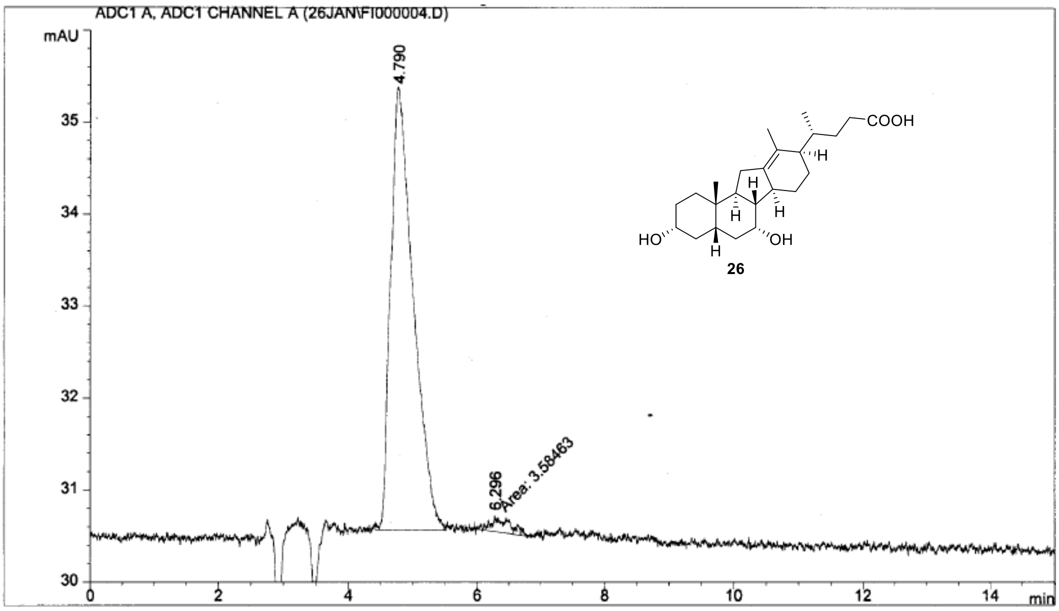

4.6. HPLC trace of 3:

Method conditions:

Column:	Phenomenex Kinetex 2.6 µM C-18, pore size = 100 Å C-18 100 × 30 mm
Column Temp:	40 °C
Detection:	CAD
Flow Rate:	0.5 mL/min
Sample Solvent:	Methanol (1 µL injection)
Mobile Phase A:	Water + 0.1% Formic acid
Mobile Phase B:	Methanol
Gradient: (% B):	T0=30, T7=100, T9=100, T10=30, T12=30

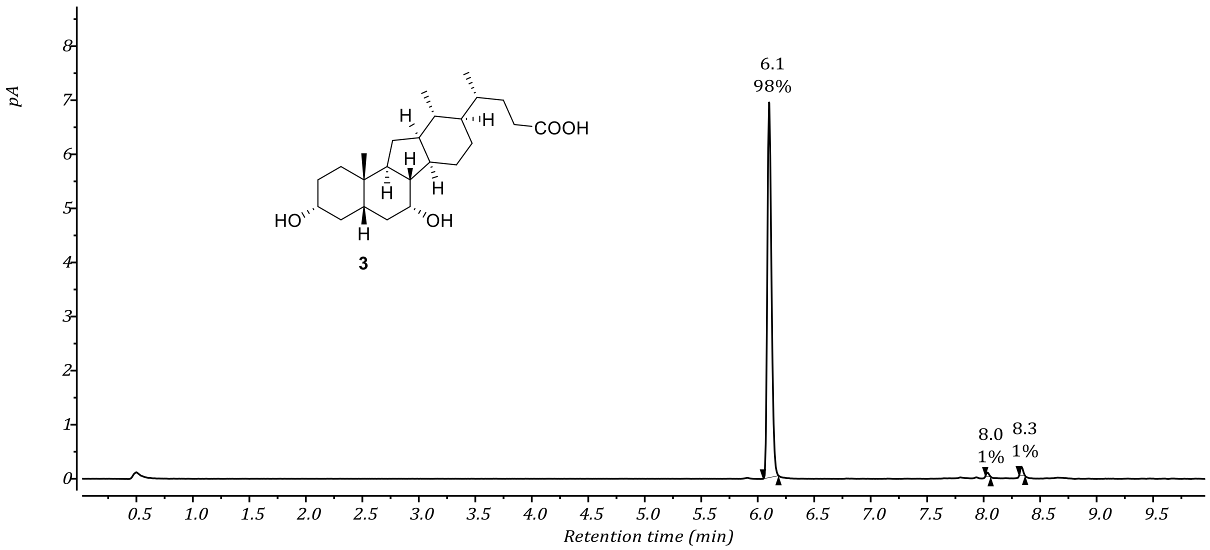

4.7. LCMS trace of compound 24:

Column:	Poroshell 2.6 µM C-18, pore size = 100 Å C-18 100 × 30 mm
Column Temp:	40 °C
Detection:	ELSD (temp 60 °C)
Flow Rate:	1 mL/min
Sample Solvent:	Methanol (1 µL injection)
Mobile Phase A:	5 mM Ammonium formate and 0.012% Formic acid in Water
Mobile Phase B:	5 mM Ammonium formate and 0.012% Formic acid in Methanol
Gradient: (% B):	T0=50, T7=100, T9=100, T9.5=50, T10=50

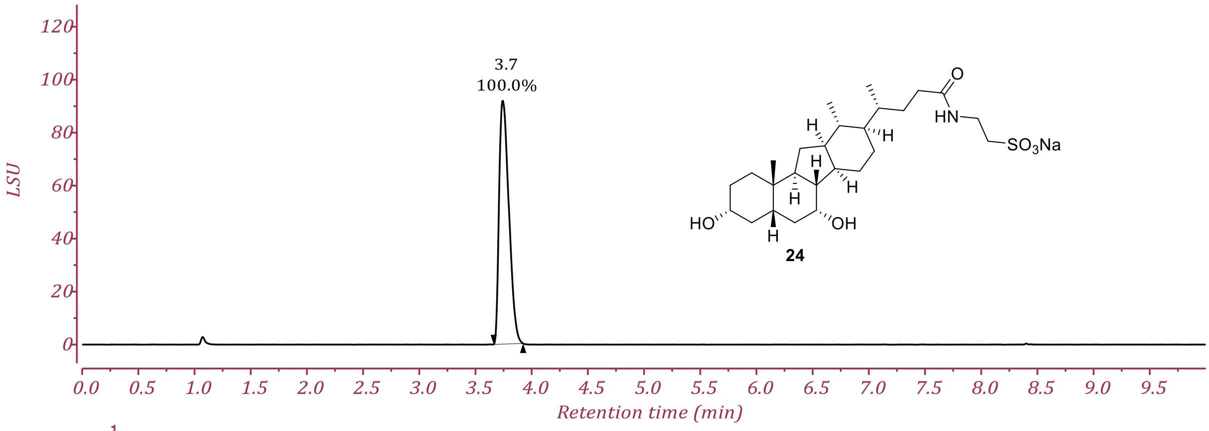

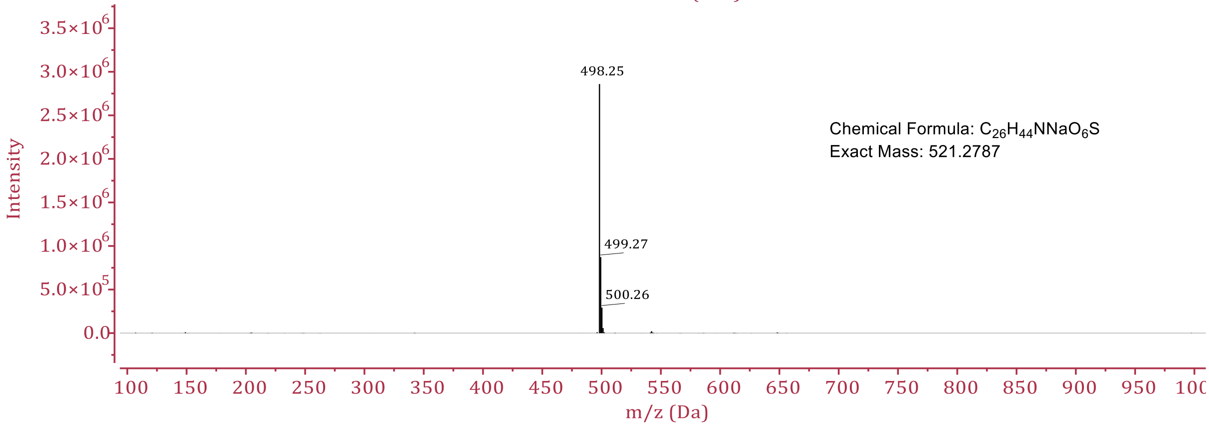

Supplement: Supplementary file 1 [file biomolecules-13-00076-s001.zip › biomolecules-2108852-supplementary.pdf]
